# AMPA receptors in the synapse turnover by monomer diffusion

**DOI:** 10.1038/s41467-019-13229-8

**Published:** 2019-11-20

**Authors:** Jyoji Morise, Kenichi G. N. Suzuki, Ayaka Kitagawa, Yoshihiko Wakazono, Kogo Takamiya, Taka A. Tsunoyama, Yuri L. Nemoto, Hiromu Takematsu, Akihiro Kusumi, Shogo Oka

**Affiliations:** 1 Department of Biological Chemistry, Division of Human Health Sciences, Graduate School of Medicine, Kyoto University, Kyoto, 606-8507 Japan; 2 Center for Highly Advanced Integration of Nano and Life Sciences (G-CHAIN), Gifu University, Gifu, 501-1193 Japan; 3 Institute for Integrated Cell-Material Sciences (WPI-iCeMS), Kyoto University, Kyoto, 606-8507 Japan; 4 Department of Integrative Physiology, Faculty of Medicine, University of Miyazaki, Miyazaki, 889-1692 Japan; 5 Membrane Cooperativity Unit, Okinawa Institute of Science and Technology Graduate University (OIST), Onna-son, Okinawa, 904-0495 Japan; 6 Department of Molecular Cell Biology, Faculty of Medical Technology, Graduate School of Health Sciences, Fujita Health University, Aichi, 470-1192 Japan

**Keywords:** Single-molecule biophysics, Membrane trafficking, Ion channels in the nervous system, Synaptic plasticity, Synaptic transmission

## Abstract

The number and subunit compositions of AMPA receptors (AMPARs), hetero- or homotetramers composed of four subunits GluA1–4, in the synapse is carefully tuned to sustain basic synaptic activity. This enables stimulation-induced synaptic plasticity, which is central to learning and memory. The AMPAR tetramers have been widely believed to be stable from their formation in the endoplasmic reticulum until their proteolytic decomposition. However, by observing GluA1 and GluA2 at the level of single molecules, we find that the homo- and heterotetramers are metastable, instantaneously falling apart into monomers, dimers, or trimers (in 100 and 200 ms, respectively), which readily form tetramers again. In the dendritic plasma membrane, GluA1 and GluA2 monomers and dimers are far more mobile than tetramers and enter and exit from the synaptic regions. We conclude that AMPAR turnover by lateral diffusion, essential for sustaining synaptic function, is largely done by monomers of AMPAR subunits, rather than preformed tetramers.

## Abstract (web summary)

The mechanisms regulating the turnover of the AMPARs in the synapse, which is critically important to sustain basic synaptic activity, remains unclear. In this study, authors used single-molecule imaging techniques to demonstrate that AMPAR tetramers are not stable entities and readily fall apart to dimers and monomers that could reform to tetramers at the synapse, and that rapidly diffusing monomers in the plasma membrane are primarily responsible for the AMPAR turnover in the synapse.

## Introduction

Higher-level brain functions such as learning and memory require synaptic plasticity^1,2^. Among the key players for synaptic plasticity are α-amino-3-hydroxyl-5-methyl-4-isoxazole-propionate (AMPA)-type glutamate receptors (AMPARs), hetero- or homotetramers composed of four subunits GluA1–4, which might further be organized in nanoscale postsynaptic domains^3–5^. For inducing stimulation-induced synaptic plasticity, as well as for sustaining basal synaptic activity to make the synapse ready for enabling synaptic plasticity, the number and the subunit compositions of AMPARs in the synapse are carefully tuned^6–8^. Most previous reports showed that this is achieved by dynamic turnover of AMPARs in the synapse by their lateral diffusion and vesicular trafficking^9–11^. However, we found the lateral diffusion mechanism quite problematic due to the following three contradictory observations.

First, it is widely believed that AMPAR tetramers with various subunit compositions are fully and stably assembled in the endoplasmic reticulum (ER)^12,13^, and then the stable tetramers are carried to the vesicular pool near the plasma membrane (PM), as well as to the PM itself and to the postsynaptic membrane, by vesicular trafficking and lateral diffusion^11,14,15^. In fact, AMPAR tetramers formed in the ER are considered to be stable till their proteolytic decomposition^16^. It follows then that for tuning the number and subunit compositions of AMPAR in the postsynaptic membrane, the tetramers with different subunit compositions must be pooled in the ER or in the PM and carried to the postsynaptic membrane^8,11^, suggesting that the pool must be quite large. Meanwhile, a superresolution study reported that the extrasynaptic pool of AMPARs is small^17^, casting some doubt on the concept of extremely stable AMPAR tetramers.

Second, as one of the mechanisms for regulating the AMPAR number and compositions, AMPAR recruitment by lateral diffusion through the PM was found to be critically important during both the basal activity^9,18^ and synaptic plasticity:^19–22^ AMPARs are continuously recruited from the extrasyanptic PM to the synaptic PM by lateral diffusion, and those in the synapse often escape into the dendritic PM also by diffusion, although vesicular trafficking would play additional key roles^10,23^. This dynamic turnover by lateral diffusion permits neurons to sustain the basal synaptic activity by tuning the numbers and subunit compositions of AMPARs in the synapse^11^.

Third, however, previous observations clearly showed that stable AMPAR tetramers were immobile in the oocyte PM^24^. Namely, stable tetramers would be unable to undergo rapid exchanges between the dendritic PM and the synaptic/spinal PM by lateral diffusion.

Therefore, the three important previous observations, i.e., stable AMPAR tetramers, rapid exchanges of AMPARs between the synaptic/spinal PM and the dendritic-shaft PM by lateral diffusion, and AMPAR tetramer immobility in the PM are inconsistent, creating an enigmatic problem. This study was undertaken to resolve these contradictory observations, and to elucidate the fundamental AMPAR turnover mechanism by which the number and subunit compositions of AMPARs in the synapse are sustained and tuned, particularly for basic synaptic activity.

In the present research, we first examined the possibility that AMPAR tetramers are in dynamic equilibrium with their subunits’ monomers, dimers, and trimers, using single-molecule imaging. In fact, we have previously found that the heterotetramer composition of AMPARs was varied quite readily by their fucosylation in the Golgi^25^, suggesting that the AMPAR heterotetramers might not be as stable as previously assumed. If the monomers–oligomers interconversions are true, neurons could recruit the new subunits into the synapse and change the subunits composition quite readily.

Our single-molecule imaging experiments reveal that, although the GluA1 and GluA2 molecules expressed in the PM of the HEK293-cell line form homo- and heterotetramers, they are metastable and instantaneously (with lifetimes of 98 and 208 ms, respectively) fall apart into monomers, dimers, or trimers, which would again readily form greater oligomers, including tetramers. Likewise, in the dendritic-shaft PM of mouse hippocampal neurons, single-molecule spots of fluorescent GluA1 and GluA2 dynamically merge into greater oligomers and split into smaller oligomers/monomers, on similar time scales to those found in the HEK293-cell PM. In the dendritic-shaft PM, GluA1 and GluA2 monomers and dimers, rather than tetramers, readily enter and exit from the synaptic regions. Therefore, it is concluded that the turnover of AMPARs by lateral diffusion between the postsynaptic membrane and the dendritic-shaft PM, for sustaining basal synaptic activity, is conducted by AMPAR subunit monomers (and dimers), rather than preformed stable tetramers. The monomer diffusions would enable to increase the AMPAR number and change the tetramer compositions quickly in the synapse in the early phase of LTP, suggesting a novel mechanism for synaptic plasticity.

## Results

### GluA1 and GluA2 tagged with ACP or Halo7 are functional

**Fig. 1:**
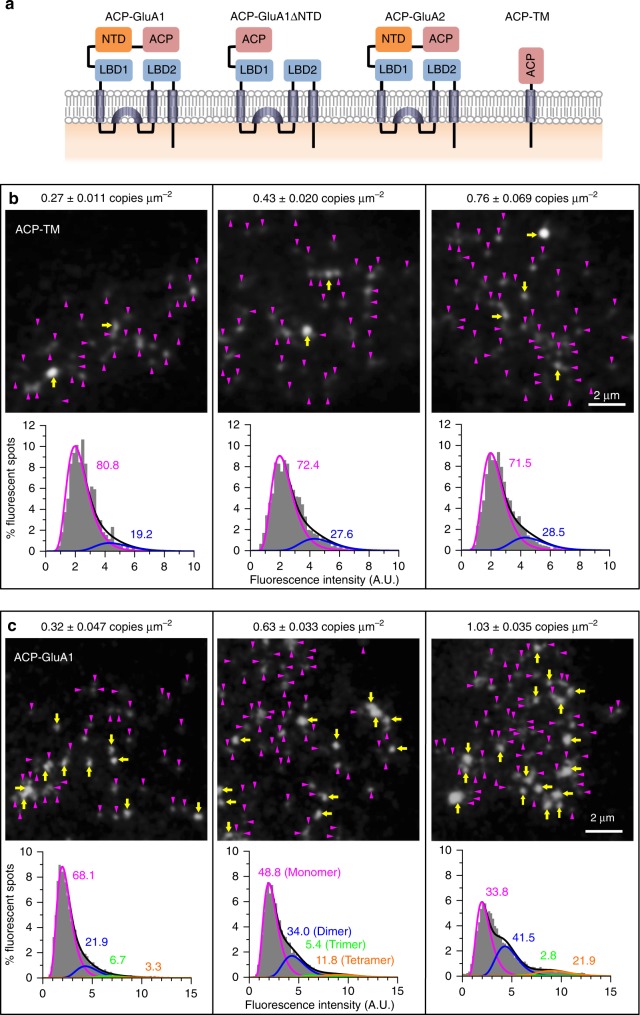
GluA1 existed as monomers, homodimers, and homotrimers as well as homotetramers when expressed in the HEK293-PM. Note that most of the data about GluA2 are shown in the Supplementary Information. **a** Schematic figure showing the structures of ACP-GluA1, ACP-GluA1ΔNTD, ACP-GluA2, and ACP-TM. LBD represents the ligand-binding domain. Representative snapshot images and distributions (histograms) of the signal intensities of individual fluorescent spots of ATTO594-labeled ACP-TM (**b**) and ACP-GluA1 (**c**) expressed in HEK293-PMs. Distributions of the signal intensities of individual fluorescent spots (histograms) were obtained at various expression levels (number densities). The numbers of examined fluorescent spots: 779, 1729, and 2192 spots for ACP-TM (from left to right in **b** and 4305, 7730, and 7107 spots for ACP-GluA1 (from left to right in **c**). Each distribution was fitted with the sum of two (for ACP-TM, **b**) or four (for ACP-GluA1, **c**) lognormal functions, representing the fractions of monomers (magenta), homodimers (blue), homotrimers (green), and homotetramers (orange). Numbers in the figure indicate molecular fractions. Magenta arrowheads and yellow arrows in the images indicate spots with fluorescence intensities of <3.6 and >3.6 arbitrary units (A.U.). This threshold intensity was determined as that at which the ratio of the copy numbers of ACP-TM with higher vs. lower intensities in the experimental histogram became the same as the ratio of apparent dimers vs. monomers determined by the lognormal fitting. Related ACP-GluA2 results are shown in Supplementary Fig. 3b

GluA1 and GluA2, tagged with the acyl carrier protein (ACP) or Halo7 at their N-termini, were expressed in the PM of HEK293 cells (referred to as ACP-GluA1, ACP-GluA2, Halo7-GluA1, and Halo7-GluA2; Fig. 1a; Supplementary Fig. 1a, b). Whole-cell current recordings of the HEK293 cells expressing the tagged GluA1 or GluA2 exhibited glutamate-induced channel activities characteristic of AMPARs, suggesting that these tagged AMPAR subunits are functional (Supplementary Fig. 2). These tagged GluA1 and GluA2 molecules, as well as the monomer reference molecules, the transmembrane (TM) domain of the LDL receptor fused to the ACP or Halo7-tag at its N-terminus (ACP-TM or Halo7-TM), expressed in HEK293 cells, were labeled with fluorescent dyes (ATTO594 or Rhodamine110) at nearly 100%, as determined by single-molecule imaging using total internal reflection fluorescence (TIRF) microscopy (Supplementary Fig. 1c, d).

### GluA1 and GluA2 exist as monomers, dimers, and oligomers

To examine the oligomeric states of ACP-GluA1 and ACP-GluA2, we first observed a monomer reference molecule, ATTO594-labeled ACP-TM, expressed at various number densities in the PM of HEK293 cells (Fig. 1). The image exhibited fluorescent spots with quite uniform signal intensities, when the expression level was 0.13 copies μm^−2^ in the PM (Supplementary Fig. 3a). With an increase in the expression level to 0.76 copies μm^−2^ in the PM, the fluorescent spots exhibiting the signal intensities of apparent dimers were increased, due to the increased incidental encounters of two molecules within the diffraction limit distance of ~240 nm (Fig. 1b). To quantitatively evaluate the fractions of ACP-TM monomers and apparent dimers, the signal intensities of all of the observed individual spots were measured in each image, and the distribution of the signal intensities was fitted by the sum of two lognormal functions, representing the spots with monomeric and apparently-dimeric signal intensities (Fig. 1b bottom).

**Fig. 2:**
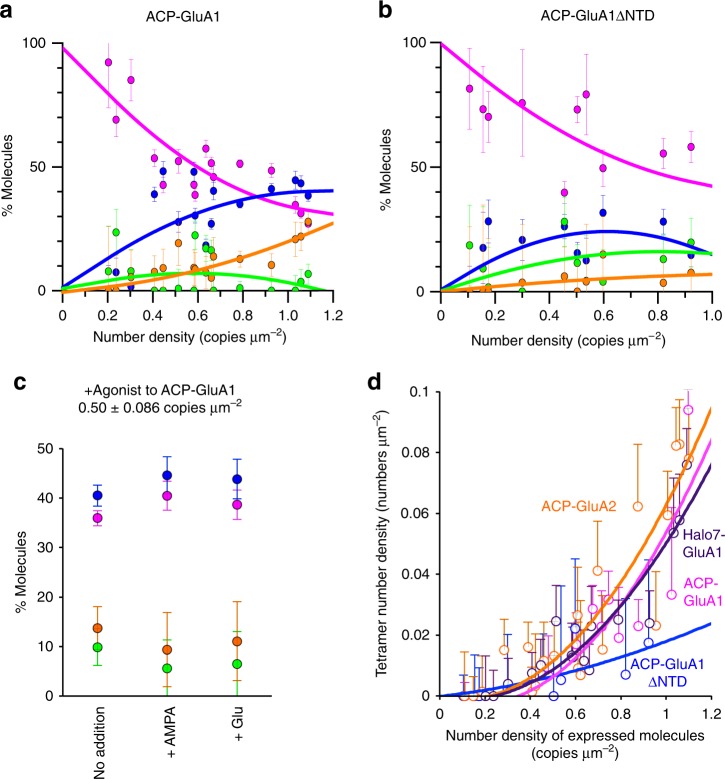
With an increase of the GluA1 expression level, the monomer fraction is decreased whereas the homotetramer fraction is increased. **a** The fractions of ACP-GluA1 molecules that exist as monomers, homodimers, homotrimers, and homotetramers (magenta, blue, green, and orange circles and lines, respectively, which are the same for all of the panels) are plotted as a function of the number density of ACP-GluA1 molecules expressed in the HEK293-PM. Curves are to help the eye (curve fitting with quadratic functions). Error bars in all panels represent standard errors. The numbers of independent experiments conducted to obtain the results shown in this figure are summarized in Supplementary Tables 2 and 3. Related ACP-GluA2 results and Halo7-GluA1 data are shown in Supplementary Figs. 4a and 5a, respectively. **b** The same as **a**, but for ACP-GluA1ΔNTD. **c** The effects of the agonists (0.1 mM AMPA [+AMPA, *n* = 5 cells] and 10 mM l-glutamate [+Glu, *n* = 5 cells]; No addition, *n* = 7 cells) on the fractions of ACP-GluA1 molecules that exist as monomers, homodimers, homotrimers, and homotetramers (ACP-GluA1 expressed at 0.50 ± 0.09 copies μm^−2^). Related ACP-GluA2 results are shown in Supplementary Fig. 4b. **d** Tetramer number densities of ACP-GluA1, ACP-GluA2, Halo7-GluA1, and ACP-GluA1ΔNTD, plotted as a function of the number density of respective molecules expressed in the HEK293-PM. Curves are to help the eye (curve fitting with quadratic functions). The ACP-GluA2 data shown here are based on the data shown in Supplementary Fig. 4a

At variance with the previous expectations, the single-molecule images of ATTO594-labeled ACP-GluA1 and ACP-GluA2 expressed in the HEK293-cell PM revealed the presence of many fluorescent spots with signal intensities comparable to those of monomeric ACP-TM, although spots with greater intensities were also found (Supplementary Movie 1; Fig. 1c; Supplementary Fig. 3b; in the following, when the meaning is clear, we simply state GluA1 and GluA2 rather than ACP-GluA1 and ACP-GluA2). These results clearly showed that majorities of GluA1 and GluA2 molecules exist as monomers. Quantitative evaluations of the signal intensity distributions of individual GluA1 and GluA2 fluorescent spots, using the sum of four lognormal functions (for fluorescent spots representing monomers, and two, three, and four colocalized molecules), demonstrated that considerable fractions of GluA1 and GluA2 molecules were monomers and homodimers, rather than homotetramers (Fig. 1c bottom and Supplementary Fig. 3b bottom; Supplementary Table 1). With an increase in the number densities of GluA1 and GluA2 molecules expressed in the HEK293-cell PM, the fractions of apparent homotetramer spots were increased, whereas that of the apparent monomer fraction was decreased (Fig. 2a; Supplementary Fig. 4a; Supplementary Table 2a, b; the same occurred Halo7-GluA1 as shown in Supplementary Fig. 5a and Supplementary Table 2c). Similar results were obtained with GluA1 and GluA2 conjugated with mGFP at their C-termini (Supplementary Fig. 6).

**Fig. 3:**
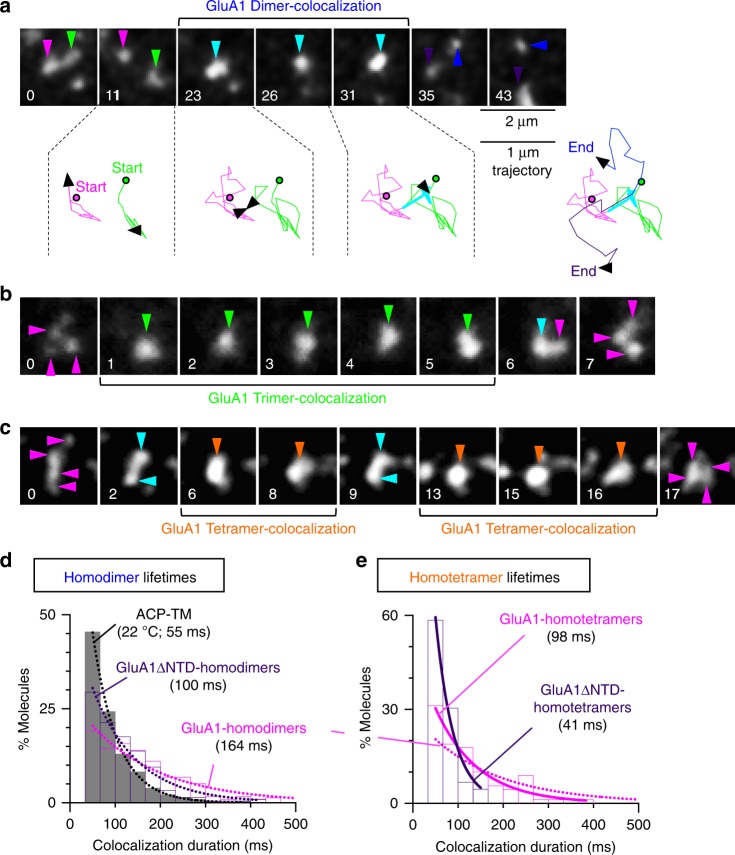
GluA1 monomers form metastable homooligomers with exponential lifetimes of <200 ms, and dissociate into monomers. Typical single-molecule fluorescence image sequences of two, three, and four diffusing ACP-GluA1 molecules undergoing transient dimerization, trimerization, and tetramerization in the HEK293-cell PM (from 236, 54, and 90 independent movies; **a**, **b**, and **c**, respectively). In the image sequence in **a**, two ACP-GluA1 molecules (green and magenta arrowheads) became colocalized in frame 23 (33 ms per frame, normal video rate) and diffused together for the next eight frames (for a 0.30-s total colocalization time; light blue arrowheads), until they became separated and diffused independently (blue and purple arrowheads). Colors of the subtrajectories are the same as those for the arrowheads (the circle and arrow show the locations at the start and at the time in the frame number on top, respectively). Since these experiments were conducted with a single color (ATTO594), after the dimerization event, which molecule corresponds to each one before dimer formation could not be determined (hence, different colors in the trajectory). **b**, **c** The spots with the intensities of monomers, dimers, trimers, and tetramers are indicated by magenta, blue, green, and orange arrowheads. **d** The distributions of the homodimer durations for ACP-GluA1, ACP-GluA1ΔNTD, and ACP-TM, obtained by measuring the durations of all of the observed homo-dimerization events. The distribution for ACP-TM was obtained at 22 °C, a temperature at which ACP-TM’s diffusion coefficient becomes practically the same as those of ACP-GluA1 and ACP-GluA1ΔNTD observed at 37 °C. Each histogram could be fitted well with a single exponential decay function (dotted curves), providing the dimer lifetime (given in parentheses after correction for the photobleaching lifetime of the fluorescent probe). Related data for Halo7-GluA1 and Halo7-TM are shown in Supplementary Fig. 5b. **e** The distributions of the homotetramer durations of ACP-GluA1 and ACP-GluA1ΔNTD. Each histogram could be fitted well with a single exponential decay function (solid curves), providing the tetramer lifetime (dotted curves for GluA1 homodimers). For statistical parameters, see Supplementary Table 4. Related GluA2 results are shown in Supplementary Fig. 8

The deletion of the GluA1′s N-terminal domain (NTD), which was proposed to be involved in the dimerization/oligomerization of AMPARs^12,26,27^, from ACP-GluA1 (ACP-GluA1ΔNTD) increased the fractions of monomer-like fluorescent spots whereas it reduced those of tetramer-like spots, as compared with those of the full-length ACP-GluA1, at all number densities of the expressed molecules in the PM (Fig. 2b; Supplementary Table 2d). This result strongly suggests that the monomer-like and colocalized fluorescent spots of GluA1 and GluA1ΔNTD, and probably GluA2, represent true monomers, dimers, trimers, and tetramers, rather than incidentally encountered (incidentally colocalized) spots, and therefore, hereafter, we simply use the simple terms, monomers, dimers, trimers, and tetramers (further confirmed later by their lifetimes, see Fig. 3).

The results shown here clearly indicate that the deletion of the NTD did not totally block the dimerization/oligomerization of ACP-GluA1ΔNTD, in agreement with the previous observations in which GluA1 dimers are stabilized by, in addition to the homo-interaction of the NTD, the homo-interactions between ligand-binding domains and between TM domains^12^. Furthermore, the increased monomer fraction of GluA1ΔNTD, as compared with GluA1, is consistent with the increased mobility of GluA2ΔNTD relative to that of GluA2 in spines, as reported previously^28^. These results are not due to the presence of large fractions of non-labeled ACP-GluA1, ACP-GluA2, and Halo7-GluA1 molecules, because their labeling efficiencies were almost 100% (Supplementary Fig. 1c, d). The molecular fractions of monomers, homodimers, homotrimers, and homotetramers of GluA1 or GluA2 expressed in the HEK293-cell PM were not altered by the addition of AMPAR agonists, AMPA and l-glutamate (Fig. 2c; Supplementary Fig. 4b; Supplementary Table 3), suggesting that AMPAR agonists do not enhance oligomerization, although they induce the channel activities of AMPAR tetramers.

Since AMPARs should function as tetramers, the number densities of tetramers were plotted as a function of the number densities of GluA1 (both ACP-GluA1 and Halo7-GluA1), GluA1ΔNTD, and ACP-GluA2 expressed in the HEK293-PM (Fig. 2d; Supplementary Table 2). The tetramer density sharply increased with an increase in the number densities of expressed molecules of ACP-GluA1, Halo7-GluA1, and ACP-GluA2, and their plots virtually coincided. Meanwhile, the plot for GluA1ΔNTD exhibited a much lower tendency to form tetramers.

### GluA1 and GluA2 undergo monomer–oligomer interconversions

Next, we examined the single-molecule dynamics of GluA1 and GluA2 in the PM of HEK293 cells at video rate. Virtually all of the fluorescent spots exhibited diffusion; monomers diffused fastest, and dimers and tetramers diffused 3× and 11× slower than monomers (average of GluA1 and GluA2), respectively (Supplementary Fig. 7a–c). The moments at which dimers, trimers, and tetramers were formed were directly captured by single-molecule imaging, and importantly, these dimers and oligomers fell apart quickly into monomers after they formed, and diffused away from each other (Fig. 3a–c; Supplementary Fig. 8a–c). That is to say, virtually all of the homodimers and homooligomers existed transiently, indicating that they were metastable oligomers.

We measured the duration that every oligomer we detected lasted for, and obtained the histograms of the durations of homodimers and homotetramers (Fig. 3d, e; Supplementary Fig. 8d). The histograms could be fitted by single exponential functions, thus providing the lifetimes of oligomers (after correction for the probe photobleaching lifetimes; see “Methods” section). Surprisingly, their lifetimes were in the range of as short as 75–164 ms (Table 1; Supplementary Table 4), and no stable homodimers or larger oligomers were found. Interestingly, the lifetime decreased with an increase of the oligomer size (the homooligomer lifetimes were similar between GluA1 and GluA2), suggesting that the homotrimers/homotetramers found here were not non-specific aggregates of GluA1 or GluA2, because if they had been, then their lifetimes should have been longer than those of dimers.

**Table 1:** Summary of colocalization durations of fluorescently-labeled, tagged molecules of GluA1, GluA1ΔNTD, GluA2, and TM in the HEK293-PM

Molecules	Colocalizations (Oligomers)	Colocalization lifetimes (Mean ± SEM) (ms)^a^	*P* value (Log-rank test)	Probes	Temp. (°C)	Related Figures
ACP-TM (Monomer reference)	Incidental colocalization	55 ± 1^*1 b^	–	ATTO594	22	3, S8d
Halo7-TM (Monomer reference)	Incidental colocalization	49 ± 3^*2^	–	Rho110^c^	22	S5b, S8e
ACP-TM Halo7-TM (Monomer reference)	Incidental colocalization	52 ± 4^*3^	–	ATTO594 Rho110	22	4d, S9a
ACP-GluA1	GluA1 homo-D^d^	164 ± 16^*4,Y1^	2.1 × 10^−18^	ATTO594	37	3d, 3e, 5a
	GluA1 homo-Tri^d^	117 ± 33^*5,Y4^	7.4 × 10^−3^			None
	GluA1 homo-T^d^	98 ± 11^*6,Y4,N5^	^Y4^1.1 × 10^−3^ ^N5^0.92			3e, 4e
ACP-GluA1ΔNTD	GluA1ΔNTD homo-D	100 ± 8^Y4^	2.1 × 10^−7^	ATTO594	37	3d
	GluA1ΔNTD homo-T	41 ± 6^Y6^	8.2 × 10^−10^			3e
Halo7-GluA1	GluA1 homo-D	164 ± 17^Y2,N4^	^Y2^4.7 × 10^−9^ ^N4^0.74	Rho110	37	S5b
ACP-GluA1 Halo7-GluA1	GluA1 homo-D	177 ± 11^*7,Y3^	1.3 × 10^−25^	ATTO594 Rho110	37	4d, S9b
ACP-GluA2	GluA2 homo-D	156 ± 16^*8,Y1,N4^	^Y1^8.2 × 10^−14^ ^N4^0.60	ATTO594	37	S8d, S10a
	GluA2 homo-Tri	91 ± 8^*9,N5,Y8^	^N5^0.54 ^Y8^7.0 × 10^−5^			None
	GluA2 homo-T	75 ± 9^*10,N6,Y8,N9^	^N6^0.32 ^Y8^4.4 × 10^−5^ ^N9^0.57			4e, S8d
Halo7-GluA2	GluA2 homo-D	160 ± 18^*11,Y2,N8^	^Y2^2.7 × 10^−10^ ^N8^0.80	Rho110	37	S8e, S8f
Halo7-GluA2 in CHO-K1 cells^e^	GluA2 homo-D	168 ± 9 ^N11^	0.34	Rho110	37	S8f
ACP-GluA2 Halo7-GluA2	GluA2 homo-D	181 ± 12^*12,Y3^	2.9 × 10^−27^	ATTO594 Rho110	37	4d, S9b
ACP/Halo7-GluA1 Halo7/ACP-GluA2	GluA1/A2 hetero-D^f^	334 ± 34^Y7,Y12^	^Y7^1.0 × 10^−10^ ^Y12^7.3 × 10^−12^	ATTO594 Rho110	37	4d
	GluA1/A2 hetero-T^f^	208 ± 14^Y6,Y10^	^Y6^6.1 × 10^−8^ ^Y10^4.9 × 10^−10^			4e

^a^Colocalization lifetimes after correction for the photobleaching lifetimes

^b^The superscripts *, ^Y^, and ^N^ indicate statistical test results. The distribution selected as the basis for the comparison is shown by the superscript *. Different numbers (1–12) indicate different bases. The superscript ^Y^ or ^N^ indicates that the distribution is or is not significantly different from that of the basis distribution, based on the *p* value of the log-rank test (the next column on the right) being either smaller or greater than 0.05, respectively

^c^Rhodamine110

^d^D, Tri, and T indicate dimers, trimers, and tetramers, respectively

^e^Only the results shown in this line were obtained in CHO-K1 cells, for comparison with the data obtained in HEK293 cells

^f^The average values of the colocalization lifetimes (after correction for the photobleaching lifetimes) of heteropairs of ACP-GluA1 and Halo7-GluA2 and heteropairs of Halo7-GluA1 and ACP-GluA2 (see Supplementary Table 6)

Meanwhile, these lifetimes are substantially longer than those of the incidental colocalization lifetimes found for the monomer reference molecules (55 and 49 ms for ACP-TM and Halo7-TM, respectively, which were evaluated at 22 °C, at which their diffusion coefficients match those of monomeric GluA1 and GluA2 at 37 °C; Supplementary Fig. 7d; Supplementary Table 5; for the treatment of incidental colocalizations, see Supplementary Fig. 8d). Furthermore, the lifetimes of homodimers and homotetramers did not depend on the tag protein or the cell types used (Supplementary Figs. 5b, 7d, and 8e, f; Table 1 and Supplementary Table 5).

The lifetime of the ACP-GluA1ΔNTD homodimers was shorter by a factor of approximately two (100 ms; Fig. 3d; Table 1) than that of the ACP-GluA1 homodimers, but it is still about twice as long as the incidental colocalization lifetime of 55 ms. Likewise, the homotetramer lifetime of ACP-GluA1ΔNTD is about half of that of the full-length GluA1 (Fig. 3e; Table 1). These results are in agreement with the previous observations, which showed that (1) the NTD is involved in dimerization/tetramerization of AMPARs and (2) GluA1 dimers are additionally stabilized by the interactions of the ligand-binding domains and those of the TM domains^12^. While the lifetime of ACP-GluA1ΔNTD tetramers would be very short (Fig. 3e), the tetramer fraction of ACP-GluA1ΔNTD is smaller than that of full-length GluA1 only by factors of 3–4 at the number densities of 1–1.2 copies μm^−2^ in the PM (Fig. 2d; the tetramer number densities include the effect of lifetime variations), suggesting that the tetramers of ACP-GluA1ΔNTD would form quite readily, probably as fast as the full-length GluA1, but they fall apart more quickly due to the absence of the NTD.

These results suggest that monomers, homodimers, and larger homooligomers of GluA1 and GluA2 are in dynamic equilibrium in the PM; i.e., even homotetramers are not stable entities. This conclusion is at variance with previous assumptions, which stated that the AMPARs exist as constitutive stable tetramers in the PM^12,24,29–32^.

### GluA1/A2-heteromers are more stable than respective homomers

**Fig. 4:**
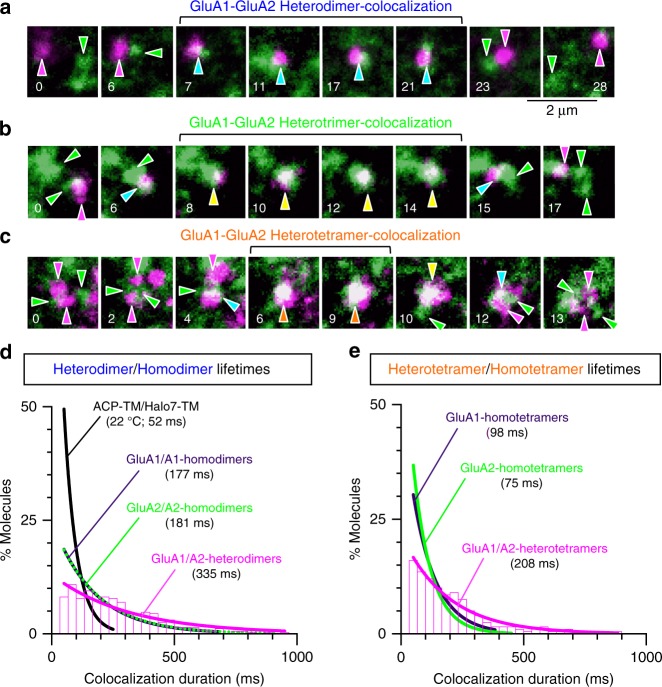
Heteromer lifetimes are longer than those of homomers. Typical single fluorescent-molecule image sequences, showing that ACP-GluA1 (magenta spots) and Halo7-GluA2 (green spots) form transient heterodimers (**a**), heterotrimers (**b**), and heterotetramers (**c**) in the HEK293-PM (typical results from 128 or more independent movies, respectively). Magenta and green arrowheads indicate ACP-GluA1 and Halo7-GluA2 monomers, respectively, cyan arrowheads indicate heterodimers, yellow arrowheads show heterotrimers (**b, c**), and orange arrowheads indicate heterotetramers (**c**). **d** The distribution of the durations of GluA1–GluA2-heterodimers (magenta), shown together with those of homodimers of GluA1–GluA1, GluA2–GluA2, and TM–TM, obtained by simultaneous two-color imaging using ACP- and Halo7-tags. Here, the colocalizations of a protein of interest using different tag proteins (ACP and Halo7 proteins) are called homo (rather than hetero)-dimers, trimers, and tetramers because ACP and Halo7 by themselves did not exhibit any sign of homo and hetero interactions^39^, and in two-color experiments, only homodimers of different colors were included in the colocaization duration histograms. Each histogram could be fitted as those in Fig. 3d, e. Since virtually the same lifetimes were obtained from the histograms for GluA1–GluA2 heterodimers (and heterotetramers in **e**) when the tag proteins were exchanged (Supplementary Fig. 9c, d), the two histograms were averaged in this figure (and also in **e**). To avoid excessive complexity of the figure, only the best-fit functions are shown without histograms for the homodimers of GluA1–GluA1 and GluA2–GluA2 and for the incidental colocalization of ACP-TM and Halo7-TM (see Supplementary Fig. 9a, b for the histograms). The homodimer lifetimes obtained by two-color experiments were virtually the same as those obtained by single-color experiments (Fig. 3d; Supplementary Figs. 5b and 8d, e; Table 1). **e** The distribution of the durations of heterotetramers, including 1:3, 2:2, and 3:1 GluA1/A2 heterotetramers (any heterotetramers detected with two colors), shown together with those of the homotetramers of GluA1 and GluA2. Note that the distributions for the homotetramers were obtained by single-color experiments. For the duration distributions of homotetramers of GluA1 and GluA2, see Fig. 3e and Supplementary Fig. 8d. For statistical parameters, see Supplementary Table 4

Many ACP-GluA1 and Halo7-GluA2 (labeled with ATTO594 and Rhodamine110, respectively) molecules expressed in the HEK293-PM were found to be monomers, but underwent frequent transient colocalizations, as observed by simultaneous dual-color single-molecule imaging (Fig. 4a–c). This result suggests the presence of metastable heterodimers, heterotrimers, and heterotetramers, in addition to dynamic monomers. The exponential lifetimes of the heterodimers and heterotetramers were 335 and 208 ms, respectively, which were substantially longer than those of homodimers (177 and 181 ms for GluA1 and GluA2, respectively) and homotetramers (98 and 75 ms for GluA1 and GluA2, respectively) (Fig. 4d, e; Supplementary Fig. 9; Table 1 and Supplementary Table 6). The higher stabilities of the GluA1–GluA2 heteromers, as compared with those of the homomers of GluA1 or GluA2, are consistent with the previous observation that GluA1–GluA2 heteromers are the major AMPAR tetramer species in the neuron^7,33^.

### TARP2 transiently binds to GluA1 and GluA2

**Fig. 5:**
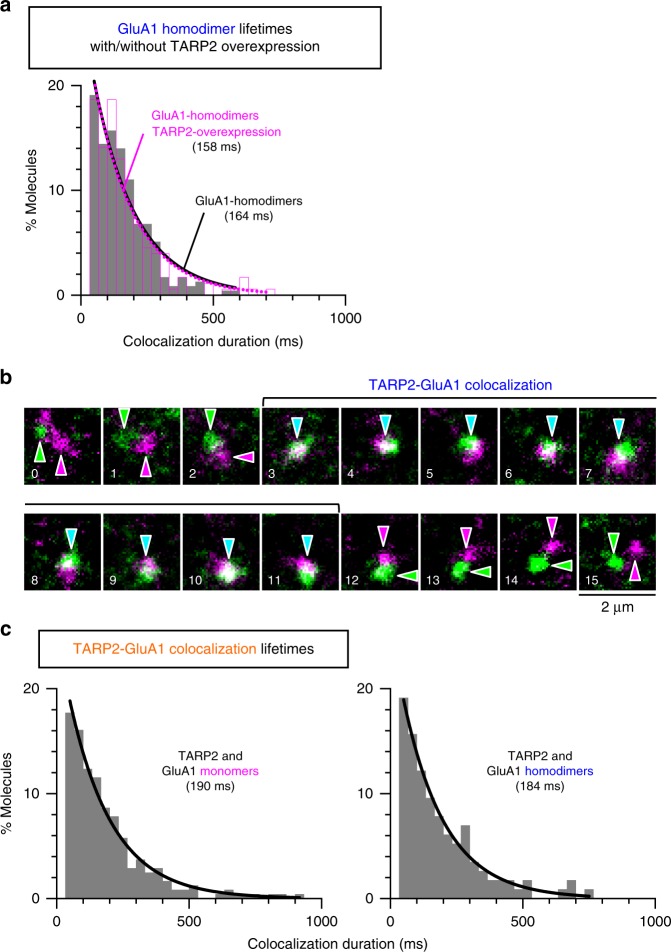
TARP2 dynamically forms metastable complexes with GluA1. **a** The distributions of homodimer durations of ACP-GluA1 in the HEK293-PM with (magenta) and without (gray, see Fig. 3d) TARP2-mGFP overexpression (expression levels 47 (±30) times higher than those of ACP-GluA1; *n* = 13 cells). **b** Typical single fluorescent-molecule image sequences, showing that a TARP2-mGFP monomer (green arrowheads) formed a transient heteromer with an ACP-GluA1 monomer (magenta arrowheads) in the HEK293-PM (a typical result from 358 independent movies). Cyan arrowheads indicate the heteromer. **c** The duration distributions of the heteromers of TARP2-mGFP with ACP-GluA1 monomers (left) and dimers (right) in the HEK293-PM. The histograms could be fitted well with single exponential decay functions, providing the heteromer lifetimes, which are shown in parentheses (after correction for the probe photobleaching lifetimes). Related ACP-GluA2 results are shown in Supplementary Fig. 10. For statistical parameters, see Supplementary Table 7

Transmembrane AMPA receptor regulatory protein 2 (TARP2), an auxiliary TM protein that controls an AMPAR channel activity^34^, has been proposed to form a stable complex with AMPAR tetramers in the ER and/or Golgi^31^. Therefore, we examined the interaction of GluA1 and GluA2 with TARP2 (mGFP was fused to the TARP2 C-terminus; TARP2-mGFP) in the HEK293-cell PM. The fusion of mGFP to TARP2′s C-terminus might inhibit TARP2 binding to PSD95 in neurons^35,36^, but would not block its binding to GluA1 and GluA2^24,37^. First, the effects of TARP2 overexpression on the homodimer lifetimes of GluA1 and GluA2 were examined by expressing TARP2-mGFP in the HEK293-PM at levels 47 (±30) times higher than those of GluA1 and GluA2. The overexpression of TARP2-mGFP did not significantly alter the homodimer lifetimes of GluA1 and GluA2 (Fig. 5a; Supplementary Fig. 10a), suggesting that TARP2 did not stabilize the homodimers of GluA1 and GluA2.

We next examined whether TARP2-mGFP indeed binds to GluA1 and GluA2 (monomers and homodimers). We found that TARP2-mGFP bound to GluA1 and GluA2, but unexpectedly, its binding was quite transient, lasting for fractions of a second. Furthermore, its lifetime of binding to homodimers was virtually the same as that to monomers for both GluA1 and GluA2 (~200 ms for all of the combinations; Fig. 5b, c; Supplementary Fig. 10b, c; Supplementary Table 7).

In summary, TARP2 neither formed stable complexes with monomers or homodimers of GluA1 and GluA2, nor induced stabilizations of GluA1 and GluA2 homodimers (and probably homotetramers; see the caption to Supplementary Fig. 10). TARP2 only formed transient complexes with both monomers and homodimers (and probably homotetramers) of GluA1 and GluA2, and the off rates of TARP2 from the homodimers of GluA1 and GluA2 were not significantly different from those from their respective monomers (approximately inverse of ~200 ms).

The (apparent) stable complexes of TARP2 with AMPAR subunits, which have been suggested to exist in synapses, might occur due to the higher concentrations of TARP2 bound to (and perhaps immobilized on) PSD95 clusters in the synapse. This could induce the continual rebinding of AMPAR subunits to the concentrated TARP2 on PSD clusters as soon as the AMPAR subunits dissociate from TARP2, making the AMPARs virtually immobile and confining them in the PSD95-concentrated synaptic regions (no long-range lateral diffusion). Such a dynamic mechanism for molecular assemblies might be generally important, in addition to synaptic molecular assemblies (such as focal contacts and adhesions and immunological synapses), for inducing and maintaining molecular assemblies that can be regulated and disintegrated as needed.

### Transient GluA1 homotetramers function as ion channels

**Fig. 6:**
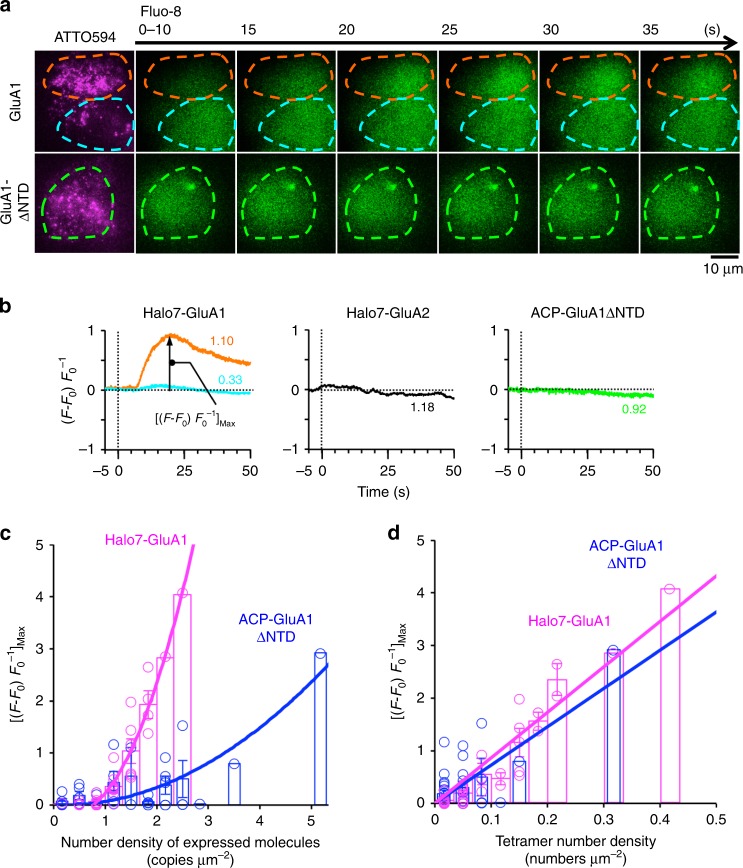
Transient GluA1 homotetramers form Ca^2+^ channels. **a** Typical time series of images of the Ca^2+^-sensitive dye Fluo-8 incorporated in the cytoplasm of HEK293 cells after l-glutamate stimulation (green images), with the images of ATTO594-labeled Halo7-GluA1 and ACP-GluA1ΔNTD expressed in the same cells (magenta images). (Top row) Two cells expressing Halo7-GluA1 at high (1.10 copies μm^−2^) and low (0.33 copies μm^−2^) number densities exhibited large or virtually no Ca^2+^ responses, respectively (typical results from 36 independent time series). (Bottom row) A cell expressing ACP-GluA1ΔNTD at a high number density (0.92 copies μm^−2^), exhibiting no changes in the cytoplasmic Ca^2+^-concentration (among 38 independent time series). Since we were unable to express ACP-GluA1 at number densities of 1.10 copies μm^−2^ or higher, for these experiments, we employed Halo7-GluA1, which could be expressed up to 2.44 copies μm^−2^. Dashed lines indicate the cell peripheries. **b** Time courses of the %increases in the Fluo-8 fluorescence intensity ([*F*-*F*_0_] *F*_0_^−1^, where *F* and *F*_0_ represent the fluorescence intensities in the cytoplasm after background subtraction, measured at times *t* and 0, respectively) after stimulation (time 0, vertical dotted line). The values in the graph represent the number densities of molecules expressed in the PM (copies μm^−2^). (left) The time courses for the cells on the top (orange) and bottom (cyan) shown in **a**. (middle) The time course for cells expressing Halo7-GluA2 at a number density of 1.18 copies μm^−2^ (among 3 independent time series). (right) The time course of the cells expressing ACP-GluA1ΔNTD, shown in the bottom row in **a**. **c** [(*F*-*F*_0_) *F*_0_^−1^]_Max_ (see the graph in **b**; mean ± SEM) plotted as a function of the number density of Halo7-GluA1 and ACP-GluA1ΔNTD expressed in the PM (graph with a bin size; 0.333 copies μm^−2^). Curves are to help the eye (fitting with quadratic functions). For the numbers of observed cells, see the circles (Supplementary Table 8). **d** [(*F*-*F*_0_) *F*_0_^−1^]_Max_ plotted as a function of the number density of homotetramers in the PM for Halo7-GluA1 and ACP-GluA1ΔNTD. Each graph could be fitted with a linear function

We further examined whether transient GluA1 homotetramers with a ~100-ms lifetime function as calcium channels in the HEK293-PM. The l-glutamate-induced influx of Ca^2+^ ion was examined using a Ca^2+^ indicator, Fluo-8, incorporated in the cytoplasm of HEK293 cells expressing various number densities of GluA1, GluA1ΔNTD, or GluA2 on the cell surface (Fig. 6a, b). The Ca^2+^ influx increased with an increase in the number density of GluA1 in the PM (Fig. 6c; Supplementary Table 8). Meanwhile, in the case of GluA1ΔNTD, much higher number densities in the PM were required to induce a Ca^2+^ influx (Fig. 6c; Supplementary Table 8). However, the l-glutamate-induced Ca^2+^ influx was proportional to the number density of tetramers of GluA1 or GluA1ΔNTD existing at any moment, and the slopes of the linear-fit functions for GluA1 and GluA1ΔNTD were quite similar to each other (Fig. 6d; Supplementary Table 8). This result suggests that both GluA1 tetramers and GluA1ΔNTD tetramers, even though they are transient tetramers (Fig. 3e), form Ca^2+^ channels. Namely, the Ca^2+^ inflow is proportional to the number of tetramers that existed at any moment, regardless of whether they are composed of GluA1ΔNTD or the full-length GluA1 (GluA1ΔNTD tetramers function as effectively as the full-length GluA1 as a Ca^2+^ channel). Meanwhile, the cells expressing GluA2 did not exhibit the glutamate-induced influx of Ca^2+^ ion, as expected^38^ (Fig. 6b).

### GluA1 and GluA2 exist as monomers in the dendritic-shaft PM

To examine how GluA1 and GluA2 behave in the dendritic-shaft PM in the neuron, we used a primary culture of mouse hippocampal neurons. First, we found that the molecules in the PM of the dendritic shaft (marked by GAP-43-Venus expression) diffused at slower rates than those in the HEK293-PM by a factor of ~1.56. This was determined by using two monomeric reference molecules labeled with fluorescent tags^39,40^; Cy3-DOPE, a fluorescent (non-raft) phospholipid analog, and Halo7-CD47, a TM protein with five TM domains (Supplementary Fig. 11a; Supplementary Table 9).

**Fig. 7:**
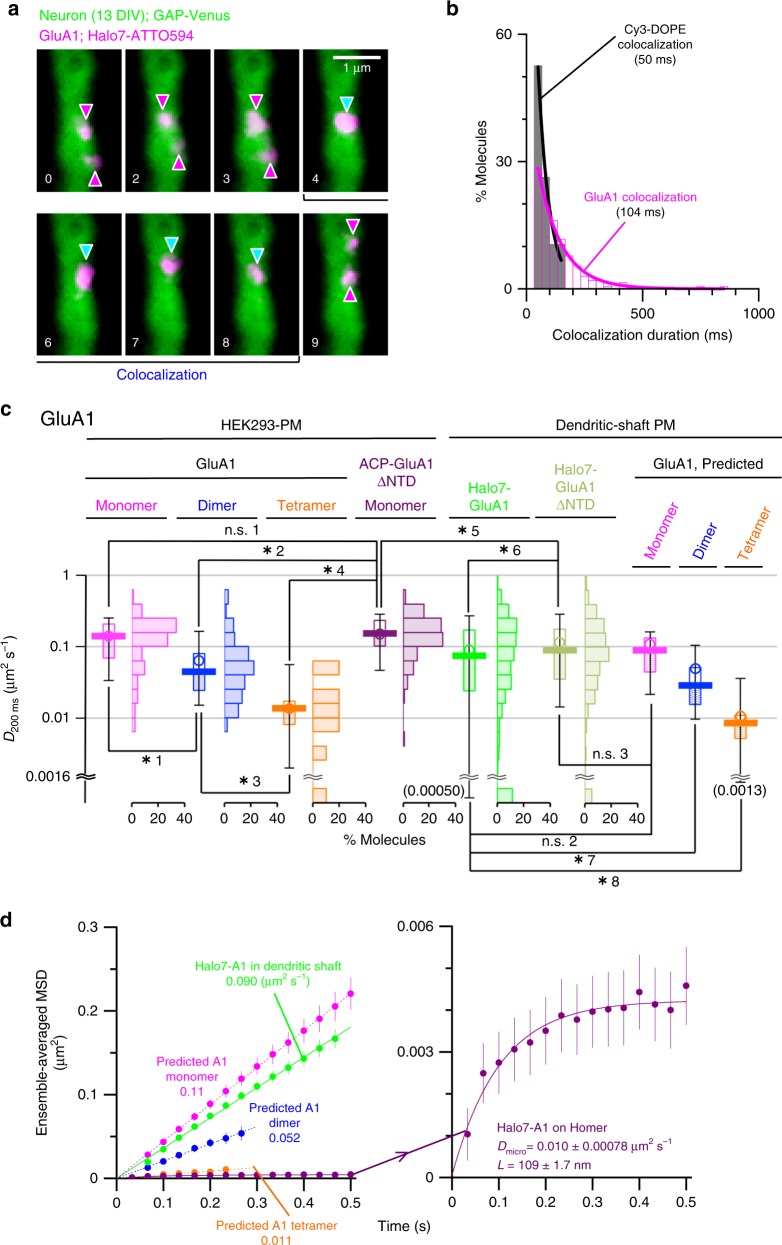
Many GluA1 molecules exist as monomers and undergo intermittent transient dimerization/oligomerization in the dendritic-shaft PM. **a** Representative image sequence of two diffusing Halo7-GluA1 fluorescent spots (labeled with ATTO594; magenta arrowheads) in a dendritic-shaft PM (13 DIV), exhibiting transient colocalization–codiffusion (cyan arrowheads; lasting for 132 ms) (representative results from 205 independent video clips). **b** The distributions of the colocalization durations of Halo7-GluA1 and Cy3-DOPE (*n* = 205 and 19 events, respectively) in the dendritic-shaft PM. Each histogram could be fitted as those in Fig. 3d, e. **c** The distributions of the diffusion coefficients (*D*_200ms_) of ACP-GluA1 monomers, homodimers, and homotetramers and ACP-GluA1ΔNTD monomers in the HEK293-PM (left), those of Halo7-GluA1 and Halo7-GluA1ΔNTD in the dendritic-shaft PM (middle), and those of presumed GluA1 monomers, dimers, and tetramers in the dendritic-shaft PM (right; Supplementary Fig. 11a, b). For the breaks at a *D*_200ms_ of 0.0016 μm^2^ s^−1^, see the caption to Supplementary Fig. 7d. Bars, circles, boxes, and whiskers indicate the median values, mean values, interquartile range (25–75%), and 10–90% range, respectively. For whiskers exhibiting diffusion coefficients smaller than 0.0016 μm^2^ s^−1^, the 10% values are shown in parentheses. Asterisks and n.s. indicate *p* < and > 0.05, respectively, using the Brunner–Munzel test (*p* values: n.s. 1, 0.23; n.s. 2, 0.23; n.s. 3, 0.54; *1, 6.7 × 10^−12^; *2, <2.2 × 10^−16^; *3, 1.6 × 10^−3^; *4, 8.6 × 10^−14^; *5, 2.9 × 10^−12^; *6, 1.7 × 10^−3^; *7, 6.2 × 10^−13^; *8, 4.4 × 10^−7^). **d** The ensemble-averaged MSD plotted against time Δ*t* (see the caption to Supplementary Fig. 7b, c). The plot is linear showing that Halo7-GluA1 underwent simple-Brownian diffusion in the dendritic-shaft PM at a mean diffusion rate between those of hypothetical ACP-GluA1 monomers and dimers (left). Meanwhile it exhibited a saturation, indicating confined diffusion within a confinement domain of 109 nm in the Homer1b-EGFP region (right; see the caption to Supplementary Fig. 12d). For the statistical parameters, see Supplementary Table 10. Related GluA2 data are shown in Supplementary Fig. 12

We next expressed Halo7-GluA1 and Halo7-GluA2 (ACP-fused molecules could not be expressed) in the PM of cultured hippocampal neurons, and after labeling with the ATTO594-conjugated Halo7-ligand, we observed their behaviors (Supplementary Movie 2; Fig. 7a; Supplementary Fig. 12a). Both Halo7-GluA1 and Halo7-GluA2 underwent thermal diffusion, and also exhibited frequent, transient colocalization–codiffusion events lasting for 104 and 95 ms, respectively, followed by separation (Fig. 7b; Supplementary Fig. 12b), just like the GluA1 and GluA2 molecules expressed in the HEK293-PM. However, since this colocalization–codiffusion may involve non-labeled endogenous AMPAR subunits, we could not determine the exact oligomeric states of the colocalized molecules. Furthermore, the individual Halo7-GluA1 and Halo7-GluA2 spots might represent AMPAR tetramers, consisting of one Halo7-labeled molecule and three non-labeled endogenous molecules, undergoing dynamic association and dissociation (of two tetramers).

To examine the latter possibility, we next monitored the diffusion of Halo7-GluA1 and Halo7-GluA2 in the dendritic-shaft PM. These molecules diffused faster in the dendritic-shaft PM than their dimers in the HEK293-PM (Fig. 7c, d and Supplementary Fig. 12c, d; Supplementary Table 10). This occurred despite the slower diffusion of the monomer reference molecules, Cy3-DOPE and Halo7-CD47, in the dendritic-shaft PM than in the HEK293-PM by a factor of 1.56 and that Halo7-GluA1 and Halo7-GluA2 formed dimers and greater multimers with the endogenous AMPAR subunits in the dendritic-shaft PM, which would further slow their diffusion. Therefore, this result suggests that Halo7-GluA1 and Halo7-GluA2 in the dendritic-shaft PM mainly existed as monomers and dimers, and thus diffused rapidly. For simplicity, we compared the diffusion coefficients of Halo7-GluA1 and Halo7-GluA2 directly evaluated in the dendritic-shaft PM with the diffusion coefficients of the hypothetical monomers, dimers, and tetramers of GluA1 and GluA2 in the dendritic-shaft PM, which were calculated from their diffusion coefficients in the HEK293-cell PM (by dividing the values by a factor of 1.56) (Fig. 7c; Supplementary Figs. 11b, c and 12c). The diffusion coefficient of Halo7-GluA1 (Halo7-GluA2) was only slightly smaller than the predicted value for its hypothetical monomers (Fig. 7c; Supplementary Fig. 12c), but significantly greater than the predicted values for their hypothetical dimers and tetramers (Fig. 7c; Supplementary Fig. 12c). This also suggested that the movements of the fluorescent spots of Halo7-GluA1 and Halo7-GluA2 in the dendritic-shaft PM, which might have represented tetrameric AMPARs, indeed represented their monomers and dimers with non-labeled endogenous AMPAR subunits, and hardly represented tetramers (with three molecules of non-labeled endogenous AMPAR subunits). Therefore, we concluded that the tetrameric AMPAR would be present only transiently and rarely in the dendritic-shaft PM, and many GluA1 and GluA2 molecules would exist as monomers and possibly dimers there, diffusing rapidly.

We further examined the diffusion of Halo7-GluA1ΔNTD in both the HEK293-PM and dendritic-shaft PM (Fig. 7c; Supplementary Tables 5 and 10). Halo7-GluA1ΔNTD expressed in the dendritic-shaft PM diffused slower than its monomers expressed in the HEK293-cell PM, by a factor of 1.73 in terms of the diffusion coefficient (*p* = 2.9 × 10^−12^; Fig. 7c). This ratio of 1.73 was quite close to the ratio of 1.56 for the monomer reference molecules Cy3-DOPE and Halo7-CD47, suggesting that Halo7-GluA1ΔNTD most of the time existed as monomers without forming complexes with the endogenous AMPAR subunits. Indeed, the median diffusion coefficient of Halo7-GluA1ΔNTD was virtually the same as that predicted for hypothetical GluA1 monomers (*p* = 0.54; Fig. 7c).

Meanwhile, Halo7-GluA1ΔNTD diffused faster than Halo7-GluA1 in the dendritic-shaft PM (*p* = 1.7 × 10^−3^; Fig. 7c; Supplementary Table 10). In the comparison of the median diffusion coefficient of Halo7-GluA1 with that of hypothetical GluA1 monomers, we could not detect a statistically significant difference (*p* = 0.23; Fig. 7c), and thus concluded that Halo7-GluA1 mostly existed as monomers. However, since we found the faster diffusion of Halo7-GluA1ΔNTD as compared with Halo7-GluA1, we concluded that although Halo7-GluA1 existed mostly as monomers, it more readily formed dimers and tetramers with other endogenous AMPAR subunits, as compared with Halo7-GluA1ΔNTD. These results are consistent with our direct detection of the transient homo-colocalization–codiffusion of Halo7-GluA1 and Halo7-GluA2, lasting for ~100 ms (average of the two molecules), in the dendritic-shaft PM. Taken together, we concluded that GluA1 and GluA2 mainly exist as monomers in the dendritic-shaft PM, undergoing rapid thermal diffusion and intermittently forming transient dimers, and possibly tetramers. The distributions of the step sizes (the distance that a molecule travels between two consecutive image frames [33 ms]), which is useful as a measure of the diffusion rates on short time scales; e.g., when a molecule exists as a part of a homodimer or homotetramer (with lifetimes of ~181 and ~98 ms, respectively; Table 1) further supported this conclusion (Supplementary Figs. 13 and 14).

The dynamics and interactions of Halo7-GluA1 and Halo7-GluA2 might be different from those of the endogenous molecules (although their electrophysiological behaviors were very similar to those of endogenous GluA1 and GluA2; Supplementary Fig. 2). Therefore, we further examined the diffusion and colocalization behaviors of endogenous GluA1 and GluA2 in the neuronal PM, by tagging these molecules with their specific monoclonal antibodies conjugated with the fluorescent probe, ATTO594. Virtually all of the observations made with Halo7-GluA1 and Halo7-GluA2 were reproduced (Supplementary Figs. 15 and 16**;** Supplementary Table 10).

### GluA1 and GluA2 in the synapse turnover by monomer diffusion

**Fig. 8:**
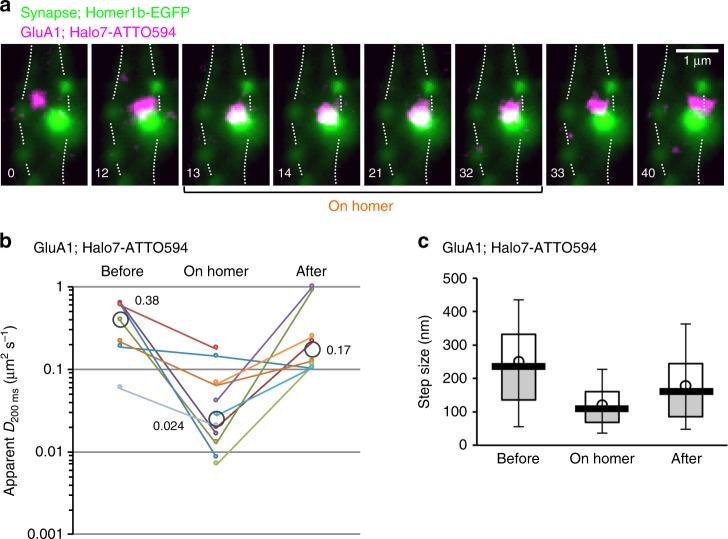
Fast diffusing GluA1 monomers and dimers enter and exit from the synaptic region. **a** A typical image sequence of a fluorescent spot of Halo7-GluA1 (labeled with ATTO594; magenta spot; representative results from 13 independent video clips), entering a synaptic region (marked by Homer1b-EGFP, green spot) from the dendritic-shaft PM and exiting from the synaptic region into the dendritic-shaft PM in cultured mouse hippocampal neurons (13 DIV). Dotted lines indicate the peripheries of the dendritic shaft. **b** The changes of apparent *D*_200ms_ for each fluorescent Halo7-GluA1 spot when it entered and/or exited from the synaptic region marked by Homer1b-EGFP (shown by small closed circles; median values are shown by larger gray open circles). The apparent *D*_200 ms_ for each fluorescent spot was simply evaluated from the increment of the MSD during 200 ms, due to the limited signal-to-noise ratios for observing single molecules in the synaptic region. (Before) In the dendritic-shaft PM right before entering the synaptic region. (On Homer) During the period in the synaptic region. (After) In the dendritic-shaft PM right after exiting from the synaptic region. Lines (and closed small circles with the same color) indicate the same fluorescent spots. **c** The changes of the step sizes of Halo7-GluA1 spots when they entered and/or exited from the synaptic region. Bars, circles, boxes, and whiskers indicate the median values, mean values, interquartile range (25–75%), and 10–90% range, respectively

We occasionally found that GluA1 and GluA2 entered the synaptic region, marked by Homer1b-EGFP (Supplementary Movie 3; Fig. 8a; Supplementary Fig. 17a). The molecules that entered the synaptic region are often those diffusing rapidly in the dendritic-shaft PM, suggesting that monomers (and some dimers) more readily enter the synaptic regions. It would be natural that the molecules with more mobility in the dendritic-shaft PM exhibit enhanced access to the spine neck.

Even after entering the synaptic region, large majorities of GluA1 molecules kept diffusing quite rapidly within the synaptic region and some readily exited from the synaptic region (Fig. 8b, c; Supplementary Fig. 17b, c). These results, as well as the observed diffusion coefficients, are consistent with the GluA2 data reported previously^41^.

In contrast, the molecules that resided in the synaptic region from the beginning of the observation were much less mobile during the entire observation period of 16 s (this is probably because only small fractions of GluA1 and GluA2 that entered the synaptic region became immobilized for longer durations), consistent with the previous observation^17^. A quantitative analysis of the trajectories suggested that Halo7-GluA1 and Halo7-GluA2 were confined within ~100-nm regions (average values of 109 and 90 nm for Halo7-GluA1 and Halo7-GluA2, respectively) for at least 16 s (Fig. 7d; Supplementary Fig. 12d). These results suggested that very limited fractions of molecules that entered the synaptic region became trapped in a domain of ~100 nm, which might be a substructure within the synapse, and that once these molecules were trapped there, they probably stayed there for more than several 10 s of seconds.

## Discussion

**Fig. 9:**
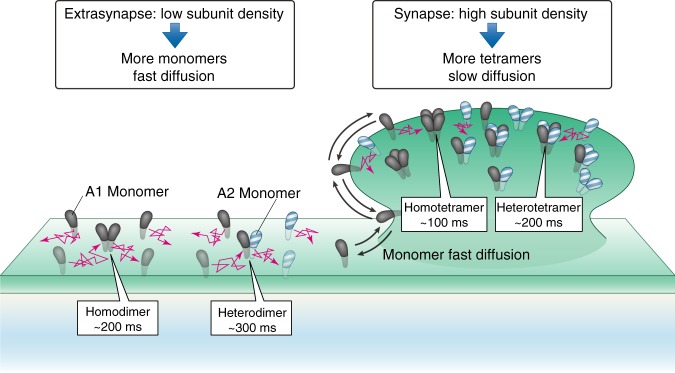
AMPAR subunits diffuse as monomers and dimers between the dendritic shaft and the synaptic region. Schematic figure showing that the AMPAR subunits GluA1 and GluA2 predominantly exist as monomers, and occasionally form transient dimers (and less frequently transient trimers and tetramers) in their reserve pools in the dendritic-shaft PM, where the subunit concentrations are low. Monomers and dimers diffuse quite freely in the dendritic-shaft PM, and enter the spines. In the synaptic regions in the spines, where the AMPAR subunit concentrations are high due to their binding to (plus their transient associations with) scaffolding protein complexes, including PSD95, GRIP, and SAP97^54^, by way of TARP2, tetramers would be the predominant species, which work as l-glutamate-evoked channels. However, the tetramer lifetimes are quite short (a lifetime of 208 ms for the heterotetramers of GluA1 and GluA2, which could be prolonged due to their binding to scaffolding protein complexes). Namely, even in the synaptic regions, tetramers become dissociated readily, whereas monomers turn into tetramers extremely quickly. In this manner, AMPARs with different compositions could be formed readily. Meanwhile, the AMPAR subunit monomers can exit from the synaptic regions quickly, further enhancing the AMPAR composition changes when they are required. This would be useful after LTP induction. As such, the associations and dissociations of AMPAR subunits would play critical roles in regulating the AMPAR functions in the synaptic membrane

Our present observations suggest that many GluA1 and GluA2 molecules exist as dynamic monomers and dimers in the PMs of neuronal dendritic shafts and undergo fast diffusion (Fig. 9; see Fig. 7; Supplementary Figs. 12, 15, and 16). They form larger transient oligomers, which fall apart readily (95–163 ms). It is likely that in the dendritic-shaft PM, monomers, (homo- and hetero-) dimers, trimers, and tetramers of GluA1 and GluA2 are in dynamic equilibrium, forming and dispersing continually, probably on the order of 100 and 200 ms for homo- and heterotetramers and 200 and 300 ms for homo- and heterodimers, respectively, as found in the HEK293-PM (Fig. 9; Table 1). The density-dependent formation of tetramers due to dynamic equilibrium could explain the apparent contradiction between the present results and the previous data. The cryo-EM single-particle analysis^33,42,43^, the AFM imaging^44^, and the X-ray crystallography^43,45^ were all performed at high AMPAR concentrations, which would increase the tetramer fractions. Of course, our results do not exclude the possibility that tetramerization is required for the AMPAR subunits to exit from the ER^12^. However, our results indicate that such AMPAR tetramers will be dispersed right after they arrive in the PM, or possibly in the Golgi^25^.

The transient tetramers would be responsible for the l-glutamate-evoked current (Supplementary Fig. 2): although the homo- and heterotetramer lifetimes are quite limited (75–208 ms), they would be sufficiently long to support the channel functions, because they are longer than the open times (0.17–7.4 ms) and burst durations (0.14–63 ms) of a single AMPAR channel^46–48^. They are even longer than the duration of the l-glutamate-evoked whole-cell current found here (~10 ms lifetime; Supplementary Fig. 2a, c). Therefore, the present results are not inconsistent with the previous electrophysiological data. We also showed that transient GluA1 homotetramers, but not monomers or smaller homooligomers, worked as calcium ion channels after l-glutamate stimulation in the HEK293-PM (Fig. 6). The agonist addition did not change the monomer–homooligomer equilibrium of GluA1 or GluA2 in the HEK293-PM (Fig. 2c; Supplementary Fig. 4b), suggesting that agonists might induce channel opening after tetramer formation, without enhancing tetramer formation.

In the dendritic-shaft PM, since many GluA1 and GluA2 molecules are in the monomeric and dimeric forms, they diffuse rapidly and thus enter the synaptic regions quite readily (Fig. 9; see Fig. 7; Supplementary Figs. 12, 15, and 16). In contrast, the tetramers were immobile or diffused slowly in the dendritic-shaft PM, consistent with the immobility of tetramers in oocytes^24^. Therefore, tetramers are not the predominant species that moves in and out between the dendritic-shaft PM and the synaptic PM by lateral diffusion. The slowed diffusion or immobilization of oligomers could be explained by the mechanism termed oligomerization-induced trapping, in which the oligomerization of membrane molecules induces extended trapping within the actin-based membrane skeleton mesh and/or elongated tethering to the actin membrane skeleton upon oligomerization^49–52^. Therefore, for the trafficking of AMPAR tetramers into and out of the synapse, vesicular transport would be responsible.

Previously, the lateral diffusion of AMPAR in the reserve pool in the dendritic-shaft PM was reported to be essential for recruiting AMPAR into the synapse for sustaining the basal synaptic activity^9,11,18^. Taken together, our results indicate that the mobile AMPARs entering and exiting from the synapses by lateral diffusion in the PM observed previously would largely represent AMPAR monomers (and dimers) and that the movement of AMPAR monomers (and dimers) into and out of the synaptic PM are indeed the key mechanism for AMPAR turnover in the synapse for sustaining the basal synaptic activity.

When the AMPAR subunit molecules entered the synapse, some formed dynamic tetramers due to the presence of much higher number densities of GluA1 and GluA2 molecules in the synapse (~5000×)^53^ and became immobilized, whereas other molecules, as well as the molecules dissociated from tetramers in the synapse, diffused out of the synapse readily (Fig. 9). Therefore, our results are consistent with previous observations that GluA1 and GluA2 mostly exist as tetramers in synapses. Note that, although the tetramer fraction would be high in the synapse, its lifetime (off-rate) would stay the same; i.e., the tetramers dissociate into smaller oligomers and/or monomers with lifetimes of 100–200 ms. However, upon dissociation, they would quickly form tetramers again, perhaps with different partners, due to the high concentrations of AMPAR subunits in the synapse. In this manner, the tetramer fraction could be kept high in synapses. Note that due to the presence of molecules that associate with AMPAR, such as GRIP and SAP97 on the PM cytoplasmic surface of the spine^54^ and TARPs in the PM^55^, the equilibrium of the oligomeric states of AMPAR subunits might not be quite as simple as that found in the HEK293-PM. For example, tetramers bound by TARP2, due to their multivalency, might bind to scaffolding proteins with affinities higher than those of monomers, leading to further slowing of their diffusion and enhanced immobilization. However, although these AMPAR-associated molecules might quantitatively alter the dynamic equilibrium of AMPAR subunits, we believe that qualitatively, the dynamic interconversions among monomers, homo- and heterooligomers, including homo- and heterotetramers of GluA1 and GluA2, dominate their behaviors.

Furthermore, we concluded that TARP2 forms transient complexes with monomers and homodimers (and probably homotetramers) of GluA1 and GluA2, with similar binding lifetimes of ~200 ms, and thus that TARP2 does not form stable complexes with monomers or multimers of GluA1 and GluA2. As in the cases of the AMPAR subunits, TARP2 might form dynamic complexes with AMPAR subunits and PSD95, becoming concentrated in the synapse and thus forming apparently stably molecular complexes with these molecules.

For inducing LTP and hippocampal learning, the AMPAR numbers must be increased and the subunit compositions have to be modulated in the synapse^20,22,56^. These could be done quite readily by recruiting monomers of the preferred subunit species to the synapse by lateral diffusion and inducing AMPAR tetramers there (Fig. 9). If the tetramers were stable as previously assumed, then the subunit composition modulations would require much longer than normally found in the LTP induction.

## Methods

### cDNA constructions

Schematic structures of the cDNA constructs used in the present study, including multiple cloning sites, signal sequences, inserted tag proteins, linkers, and the target proteins, are shown in Supplementary Fig. 18. The expression vectors Halo7-TM/pEGFP-N1, mGFP-TM/pOStet15T3, GAP-43-Venus/pCAGGs, and Halo7-CD47/pOStet15T3 were used in the present study^39,40,57^. The Homer1b-EGFP/pCAGplay vector was a kind gift from Yoshiaki Tagawa of Kagoshima University and Tomoo Hirano of Kyoto University^58^. Other plasmids were constructed using the pcDNA3.1B vector (Invitrogen). To construct the ACP-TM/pcDNA3.1B vector, the cDNA encoding the CD8 signal peptide fused to ACP in the CD8SPACP-CALR/pOStet15T3 vector^39^ was amplified by PCR, using the primer pairs 5′-tttaagcttatggccttaccagtgaccgc-3′ (aagctt = *Hind*III) and 5′-aaaactagtactccctccgccaccagacg-3′ (actagt = *Spe*I), and the cDNA encoding the TM domain of the LDL receptor in the ACP-TM/pOStet15T3 vector^39^ was amplified by PCR using the primer pairs 5′-aaaactagtagtagcgtgagggctctgtcc-3′ (actagt = *Spe*I) and 5′-tttaagcttttatcagttgatgctgttgat-3′ (aagctt = *Hind*III), and then these cDNAs were cloned into the *Hind*III site of the pcDNA3.1B vector.

Alternative splice variants, the flip and flop sequences, of GluA1 and GluA2 exist in neurons^59^, and in the present study, the flip version of mouse GluA1 cloned into pcDNA3.1B (GluA1/pcDNA3.1B)^60^ and the flop form of GluA2 cloned into pcDNA3.1B (GluA2/pcDNA3.1B)^57^ were employed because their combination provides the largest responses to the stimulations with both kainate and l-glutamate^61^. The ACP-GluA1, ACP-GluA2, Halo7-GluA1, and Halo7-GluA2 expression vectors were constructed as follows. First, a unique *Nhe*I restriction enzyme site was introduced into the GluA1/pcDNA3.1B or GluA2/pcDNA3.1B plasmid, 12 base pairs downstream from the signal peptide, using PCR mutagenesis. To construct the ACP-GluA1/pcDNA3.1B and ACP-GluA2/pcDNA3.1B expression vectors, the cDNA encoding the CD8 signal peptide fused to ACP in the CD8SPACP-CALR/pOStet15T3 vector was amplified by PCR, using the primer pairs 5′-tttaagcttatggccttaccagtgaccgc-3′ (aagctt = *Hind*III) and 5′-aaaactagtactccctccgccaccagacg-3′ (actagt = *Spe*I), and then cloned into the *Hind*III and *Nhe*I sites of GluA1/pcDNA3.1B or GluA2/pcDNA3.1B, thus replacing the signal peptide of the subunit with the CD8 signal peptide. The CD8 signal peptide promoted the expression of the proteins encoded by these constructs. To construct the Halo7-GluA1/pcDNA3.1B and Halo7-GluA2/pcDNA3.1B expression vectors, the cDNA encoding the Halo7-tag in the μ-opioid-Halo7/pEGFP-N1 vector^62^ was amplified by PCR, using the primer pairs 5′-gatgctagcggatccgaaatcggtactgg-3′ and 5′-ggcgctagcaccggaaatctccagagtag-3′ (gctagc = *Nhe*I), and then cloned into the unique *Nhe*I site introduced into the GluA1/pcDNA3.1B or GluA2/pcDNA3.1B vector.

The GluA1-mGFP/pcDNA3.1B and GluA2-mGFP/pcDNA3.1B expression vectors were constructed as follows. The stop codons of GluA1/pcDNA3.1B and GluA2/pcDNA3.1B were eliminated by introducing *Xba*I sites into the stop codons, using a QuikChange Lightning Site-Directed Mutagenesis Kit (Agilent Technologies) with the primer pairs 5′-ccttgggagccacaggattgtctagagagcagacaggaaacccttg-3′ and 5′-caagggtttcctgtctgctctctagacaatcctgtggctcccaagg-3′ for GluA1-mGFP and with the primer pairs 5′-gcatcgagagtgttaaaatttctagaatgaccttgagcgctgccac-3′ and 5′-gtggcagcgctcaaggtcattctagaaattttaacactctcgatgc-3′ for GluA2-mGFP. The cDNA encoding mGFP in the mGFP-TM/pOStet15T3 vector^41^ was amplified by PCR using the primer pairs 5′-aaattctagaatggtgagcaagggcgaggag-3′ and 5′-ctttctagattacttgtacagctcgtccatgccgag-3′ (tctaga = *Xba*I), and then cloned into the *Xba*I sites of GluA1/pcDNA3.1B and GluA2/pcDNA3.1B.

To construct the ACP-GluA1ΔNTD/pcDNA3.1B expression vector, ACP-GluA1/pcDNA3.1B was amplified by PCR, using the primer pairs 5′-tttttggtaccaagattggttactggaatgaag-3′ and 5′-tttttggtaccactccctccgccaccagacg-3′ (ggtacc = *Kpn*I), and then the PCR amplified product was self-ligated at the *Kpn*I site, resulting in the deletion of the GluA1 sequence from amino acid (a.a.) 22 to 377. The linker amino acid sequence between ACP and GluA1ΔNTD (a.a. 378–907) was thus GT.

To construct the TARP2-mGFP/pcDNA3.1B expression vector, TARP2 (without a stop codon) was amplified from mouse cDNA by PCR, using the primer pairs 5′-tttaagcttatggggctgtttgatcgagg-3′ (aagctt = *Hin*dIII) and 5′-tttgctagctacgggcgtggtccggcggttg-3′ (gctagc = *Nhe*I), and cloned into the *Hin*dIII-*Xba*I sites of pcDNA3.1B. The fragment encoding mGFP-TM/pOStet15T3 was then amplified using the primer pairs 5′-tttccgcgggagtgagcaagggcgaggag-3′ (ccgcgg = *Sac*II) and 5′-cttaccggtttacttgtacagctcgtccatgccgag-3′ (accggt = *Age*I), and cloned into the *Sac*II-*Age*I sites of pcDNA3.1B. The linker amino acid sequence between TARP2 and mGFP was thus ARGPAG.

ACP-GluA1, ACP-GluA2, Halo7-GluA1, Halo7-GluA2, and ACP-GluA1ΔNTD could not be expressed in primary cultured neurons, using the pcDNA3.1B expression vector. Therefore, we used the pCAGplay vector, in which the cDNAs of Halo7-GluA1, Halo7-GluA2, and Halo7-GluA1ΔNTD were introduced (we were unable to express ACP-GluA1 and GluA2 in neurons, and therefore, we only used Halo7-tagged molecules in the neuronal experiments). Briefly, the *Asc*I site was introduced between the *EcoR*I and *Not*I sites of the pCAGplay vector by inserting the following annealed primers, 5′‐aattggcgcgccaccggtactagtcaagcttgtcgacgaattcctgcaggatatctctagagc-3′ and 5′‐ggccgctctagagatatcctgcaggaattcgtcgacaagcttgactagtaccggtggcgcgcc-3′, which generated the sticky ends for *EcoR*I and *Not*I. The sequence encoding the signal peptide derived from interleukin-6 (MNSFSTSAFGPVAFSLGLLLVLPAAFPAP) linked to the Halo7-tag was then ligated into the *Asc*I and *Not*I sites. Finally, the cDNAs encoding GluA1 and GluA2 in the pcDNA3.1B vector (without the signal peptides of GluA1 and GluA2) were amplified by PCR, using the primer pairs 5′-tttaagcttcggccaatttccccaacaatatc-3′ and 5′-aaagcggccgcttacaatcctgtggctcccaagg-3′ for GluA1 and 5′-tttaagcttcggtctcttctaacagcatacag-3′ and 5′-aaagcggccgcctaaattttaacactctcgatg-3′ for GluA2 (aagctt = *Hin*dIII; gcggccgc = *Not*I), respectively. The fragments thus generated were then cloned into the *Hin*dIII and *Not*I sites in the pCAGplay vector, containing the cDNA sequence encoding the interleukin-6 signal peptide and Halo7-tag protein (see Supplementary Fig. 19). To construct the Halo7-GluA1ΔNTD/pCAGplay expression vector, Halo7-GluA1/pCAGplay was amplified by PCR, using the primer pairs 5′-tttgctagcaagattggttactggaatgaag-3′ and 5′-tttgctagccgaagcttgagctcgagatc-3′ (gctagc = *Nhe*I), and then the PCR amplified product was self-ligated at the *Nhe*I site.

### Cell culture, cDNA transfection, and fluorescence labeling

HEK293 cells and CHO-K1 cells (no endogenous expression of GluA1 and GluA2 in these cell lines) were purchased from American Type Culture Collection and Japanese Collection of Research Bioresources, respectively. The HEK293-cell line was authenticated by PowerPlex16 STR (contract to Promega). The CHO-K1 cells were not authenticized. Cells were proved to be mycoplasma free by using MycoAlert (Lonza). HEK293 cells and CHO-K1 cells were cultured in D-MEM (Nacalai Tesque) and α-MEM medium (Nacalai Tesque) supplemented with 10% fetal bovine serum (FBS; Sigma-Aldrich), respectively. Cells were transfected with cDNAs using Lipofectamine2000 (Invitrogen), according to the manufacturer’s instructions. The cells were seeded in glass-base dishes (35-mm diameter with a window diameter of 12 mm, 0.15-mm-thick glass; Iwaki) coated with 0.005–0.02% poly-l-lysine (Sigma-Aldrich), and cultured for 1 day before observation.

For the fluorescence labeling of Halo7-tagged proteins expressed in these cell lines and primary neurons, cells expressing Halo7-tagged molecules were incubated with 50 nM rhodamine110-conjugated Halo7-ligand (Promega) in Ham’s F12 medium (Invitrogen), supplemented with 10% FBS, at 37 °C for 15 min, and then washed with Hanks’ balanced salt solution (Nissui) buffered with 2 mM PIPES (P-HBSS, pH 7.2). Cells expressing ACP-tagged molecules were incubated with 50 nM ATTO594-Coenzyme A (Covalys Biosciences), 1 μM ACP-synthase (phosphopantetheine transferase) (New England Biolabs), and 10 mM MgCl_2_ in Ham’s F12 medium supplemented with 10% FBS, for 15 min at 37 °C and washed with P-HBSS. Cy3-DOPE in Hanks’ balanced salt solution (10 ng ml^−1^) was incorporated in the plasma membrane, by incubation at 37 °C for 15 min^39^.

All the animal experiments were conducted according to the Fundamental Guidelines for Proper Conduct of Animal Experiments and Related Activities in Academic Research Institutions under the jurisdiction of the Ministry of Education, Culture, Sports, Science and Technology of Japan and approved by the Committees on Animal Experimentation of Kyoto University. Primary hippocampal neurons were prepared from the brains of embryonic 16-day-old C57BL/6J strain mice (Shimizu Laboratory Supplies, Kyoto, Japan). Excised hippocampi were trypsinized at 37 °C for 10 min. The dispersed cells were immediately transfected with the cDNA, using an Amaxa 4D-Nucleofector system and a P3 Primary Cell 4D-Nucleofector X kit (LONZA) according to the manufacturer’s instructions, and then plated in glass-base dishes (3.0 × 10^5^ cells per dish; the dishes were treated with poly-l-lysine in the same way as those used to culture HEK293 and CHO-K1 cells), containing Neurobasal medium (Invitrogen) supplemented with 2% B27 (Invitrogen) and 500 μM l-glutamine (Gibco).

Endogenous GluA1 and GluA2 expressed in the PM of cultured neurons were tagged with their respective mAbs conjugated with the fluorescent probe ATTO594. For the fluorescent labeling of antibodies, 6.7 μM anti GluA1 mAb (a kind gift from Prof. Richard L. Huganir, Johns Hopkins University) or anti GluA2 mAb (Millipore) was incubated with 67 μM ATTO594-NHS-ester (Sigma-Aldrich) in phosphate buffered saline (PBS) at 22 °C for 2 h, and then the unreacted dye molecules were removed by dialysis against PBS using Slide-A-Lyzer Dialysis Cassettes, 10k MWCO (Thermo Fisher Scientific). For labeling GluA1 or GluA2 on the neuronal PM, 0.1 μl fluorescent antibody solutions in P-HBSS were added to cells at a 10 nM final concentration just before observation.

### Determining the cell surface expression of GluA1 and GluA2

First, HEK293 cells transfected with the cDNA encoding GluA1 or the cDNA encoding GluA2 (or those encoding the proteins fused with ACP and Halo7-tags), cultured in a 60-mm dish for 2 days, were incubated with membrane-impermeable EZ-Link sulfo-NHS-SS-biotin (1 mg ml^−1^ final concentration, Thermo Fisher Scientific) in PBS at 4 °C for 30 min. The biotin-labeled cells were lysed with 0.2 ml PBS containing 1% Triton X-100 and 0.5% Nonident P-40, and then the biotinylated proteins were captured using the streptavidin immobilized on the agarose resin (30 μl; Thermo Fisher Scientific). The agarose resin was washed with PBS containing 0.1% Triton X-100 by three rounds of centrifugation and resuspension. The proteins attached to the streptavidin agarose resin were analyzed by western blotting using anti GluA1 or anti GluA2 polyclonal antibodies (Enzo Life Sciences)^60^. Briefly, the antibody-treated blots on the nitrocellulose membrane were incubated with HRP-conjugated goat anti rabbit-IgG (Invitrogen), and then with Super Signal West Pico (Thermo Fisher Scientific), and the protein bands on the nitrocellulose membrane were imaged using a Luminoimage Analyzer LAS-3000 (Fuji Film).

### Whole-cell current recording and analysis

HEK293 cells were plated at a low density (1.5 × 10^5^ cells per well) in a well (35 mm in diameter) containing a glass coverslip (No. 1, 25 mm × 25 mm; Deckgläser) coated with collagen and gelatin. HEK293 cells were simultaneously transfected with the plasmid encoding EGFP and the plasmid(s) encoding various GluA1 and/or GluA2 fusion proteins 1 day after plating, using polyethylenimine (Polysciences). Whole-cell patch-clamp recordings were performed 1.5–2 days after transfection, under a fluorescence microscope using HEK293 cells expressing GFP. Recording electrodes were pulled from thick-walled borosilicate glass capillaries (World Precision Instruments), using a micropipette puller (Sutter Instruments) and then fire polished. The electrode resistance was 3–6 MΩ when filled with an internal solution containing 120 mM CsF, 3 mM MgCl_2_, 5 mM EGTA, and 10 mM HEPES (pH adjusted to 7.2 with CsOH). Cells were bathed continuously with a recording solution containing 145 mM NaCl, 3 mM KCl, 2 mM CaCl_2_, 1 mM MgCl_2_, 10 mM HEPES, and 10 mM glucose (pH adjusted to 7.4 with NaOH). The whole-cell current, amplified with an Axoclamp 1D amplifier (Molecular Devices) with a cutoff frequency at 5 kHz using a built-in, 4-pole low-pass Bessel filter, was digitized at a sampling frequency of 10 kHz using a Digidata 1440 A digitizer, and then recorded using the pCLAMP10 software (Molecular Devices). The l-glutamate-evoked responses were induced by a 50-ms puff application of 1 mM l-glutamate (Sigma-Aldrich) in the recording solution at a holding potential in the range between –110 and 40 mV, and the current-voltage relationships were obtained. Recordings with access resistances (Ra) greater than 20 MΩ were discarded. All recordings were performed at room temperature (23–25 °C).

### Fluoromicroscopic assay of cytoplasmic Ca^2+^ concentrations

To fluorescently label Halo7-tagged molecules expressed in HEK293 cells, the cells were incubated with 50 nM ATTO594-conjugated Halo7-linker (Promega) in Ham’s F12 medium, supplemented with 10% FBS, for 15 min and then washed with the same medium without the fluorescent linker. To observe the intracellular Ca^2+^ concentration, the labeled cells were loaded with Fluo-8AM by an incubation in P-HBSS containing 5 μM Quest Fluo-8AM (AAT Bioquest) and 0.02% Pluronic F-127 (ANASPEC) at 37 °C for 20 min, and then washed with a solution containing 140 mM NaCl, 3 mM KCl, 10 mM HEPES, 10 mM glucose, 2 mM CaCl_2_, and 1 mM MgCl_2_ (pH 7.1). Before observing the intracellular Ca^2+^ concentration, the total ATTO594 signal intensity of ATTO594-conjugated Halo7-tagged GluA1, GluA1ΔNTD, or GluA2 expressed in the bottom PM was evaluated by measuring and adding the signal intensities of all of the individual fluorescent spots. Then, using the same TIRF microscope, the Fluo-8 images of the same cell were obtained. This would measure the intracellular Ca^2+^ concentration near the bottom PM, but due to the fast diffusion of Ca^2+^ in the cytosol, we assumed that the observed Ca^2+^ concentration largely represents the bulk Ca^2+^ concentration in the cytoplasm. Glutamate stimulation was performed by adding the l-glutamate solution to the medium at a final concentration of 1 mM. The Fluo-8 image sequences were analyzed using the Image J software.

### Tracking single fluorescent molecules

Single fluorescent-molecule imaging was performed using a home-built, objective lens-type, TIRF microscope built on the basis of an Olympus IX-70 inverted microscope^50^. The precisions of the position determinations for stationary single fluorescent molecules were estimated from the standard deviations of the determined coordinates of the probes fixed on coverslips; they were ±12 nm for ATTO594 and ±17 nm for rhodamine110. All measurements of signal intensities of single individual fluorescent spots were performed only for those spots located within the central region (20 μm in diameter) of the excitation-laser illuminated area, where non-uniformities were small.

In the experiments to determine the relationship between the expression level of Halo7-GluA1, ACP-GluA1ΔNTD, or Halo7-GluA2 with the glutamate-evoked rise in the intracellular Ca^2+^ concentration, the number densities of the fluorescent spots over 1.2 copies μm^−2^ in the PM had to be measured. With these higher number densities of expressed molecules, due to the overlaps of fluorescent spots, the number densities of the expressed molecules in the PM were difficult to determine by single-molecule observations. To circumvent this problem, the total fluorescence intensity in the region of interest (ROI) in the bottom PM was measured, and after subtraction of the background (determined on the glass surface without any cells), the number density of the expressed molecules in the ROI was determined, by dividing the subtracted value by the average fluorescence intensity of single molecules (after the correction for the actual intensifier gain changes).

### Determining the fractions of monomers and oligomers

The signal intensities of all of the individual fluorescent spots detected in the initial ten frames in the single fluorescent-molecule imaging movies were determined, and each histogram was fitted by the sum of four lognormal functions^39,63,64^ using OriginPro. The signal intensity distribution for single molecules (monomers) was obtained by using the monomer reference molecules ACP-TM, Halo7-TM, and mGFP-TM expressed at low concentrations, with the fitting using the sum of two lognormal functions (the second lognormal function represents the spots due to incidental encounters of two molecules).

In our assay of the fractions of molecules that existed as monomers, dimers, trimers, and tetramers, we did not distinguish between incidental colocalizations and true molecular assemblies (therefore, when necessary, we used terms like two, three, and four colocalized molecules and apparent tetramers), due to the following two reasons. First, since the monomer reference molecules ACP-TM and mGFP-TM exhibited no incidental colocalizations that resemble trimers and tetramers in the range of the number densities of molecules employed here (Fig. 1b**;** Supplementary Figs. 3a and 6d, e), we did not need to make any corrections for the fractions of apparent trimers and tetramers. Second, although the non-distinction of true and apparent dimers in this method would cause slight overestimation of true dimers and slight underestimation of monomers, since the most important point we make in this report is the presence of considerable fractions of monomers, underestimating the monomer fraction is acceptable for the conclusions of this paper. Therefore, for the sake of simplicity, we employed the molecular fractions directly determined for fluorescently-labeled GluA1 and GluA2 molecules.

### Evaluating colocalization durations

Colocalization of two fluorescent molecules was defined as the event where two fluorescent spots, representing these molecules, become localized within 240 nm of each other^51,65^. Briefly, in single-color experiments using ATTO594, a cross-correlation analysis^39,66^ was employed. When two fluorescent spots, each representing a single molecule of ATTO594, are located close together, the probability that this method can discriminate between the two spots increases with an increase in the distance between the two ATTO594 molecules. The threshold distance for detecting one or two spots was found to occur at 240 nm in the present experimental set up (determined by the S/N of the instrument, as well as the optical diffraction limit). Using this definition, colocalized trajectories were obtained and colocalization durations were estimated.

In two-color simultaneous single fluorescent-molecule tracking experiments, using the dye pair of ATTO594 and rhodamine110, the distance between the two molecules was directly measured from the locations (*x*, *y*-positions) of each molecule (with different colors). Even when examining pairs of different-colored molecules that are known to be truly associated, the probability of scoring the two molecules as associated is limited by the localization accuracies of each molecule and the accuracies of superimposing the two images. Based on the accuracies determined here, we found that, for truly associated molecules, the probability of scoring the two molecules as associated increases to 99% when using the criterion that the molecules lie within 240 nm of each other. Therefore, we used this criterion as the definition of colocalization in simultaneous two-color single-molecule observations. This distance of 240 nm coincided with the definition of colocalization in single-color experiments. Due to this coincidence, in the present research, we defined the colocalization of two fluorescent molecules as the event where the two fluorescent spots representing these molecules become localized within 240 nm of each other.

Each time we found a colocalization event in single-molecule movies, we measured its duration, and by measuring the durations of all of the colocalization events, we obtained a histogram of colocalization durations. We found that such a histogram could be fitted by a single exponential decay function, using nonlinear least-squares fitting by the Levenberg–Marquardt algorithm provided in the OriginPro software. The colocalization lifetimes were then corrected for the photobleaching lifetimes of the probes (ATTO594, 10.2 s; rhodamine110, 8.7 s; Cy3, 5.4 s; mGFP, 1.3 s)^39^. For each fitting of the exponential decay function, a 68.3% confidence limit was obtained as the fitting error for the decay time (standard error of the mean = SEM). Statistical analysis for these distributions was performed using the log-rank test (statistical survival analysis).

We did not distinguish simple colocalizations and true molecular assemblies in the measurements of colocalization lifetimes. This is mainly due to the limitations of the time resolution and the signal-to-noise ratios obtainable for the specimens used with our instrument^39,67^. In principle, the histogram of colocalization durations of two molecules (including both apparent and true dimer cases) could be quite complex (for ease of explanation, we consider the case of dimers). Even in the case where no specific interactions between two molecules occur (incidental colocalization), the distribution of incidental colocalization durations (histogram) could be described by a complex function (*h*_incidental_). Furthermore, in the case where a specific interaction between two molecules takes place (where true dimers are formed), the two molecules first have to make close encounters within the diffraction-limited distance (this marks the beginning of the colocalization), undergo specific interactions (forming true dimers), and then after dissociation, become separated by more than the diffraction-limited distance of 240 nm (marking the end of the colocalization). Namely, the duration distribution (*h*_composite_) would be the sum of the distributions of the following three durations: the distribution from the encounter of two molecules within the distance of 240 nm until the binding of two molecules (*h*_before_), the duration of the actual binding of two molecules (*h*_true-dimers_), and the duration from the dissociation of two molecules until they become separated over the distance of 240 nm (*h*_after_). Since we did not distinguish these two cases (where the actual molecular binding does and does not occur, represented by the functions *h*_incidental_ and *h*_composite_, respectively), the function describing the observed duration distribution would be the sum of these two complex functions with the proper weight *c*; i.e., *h*_observed_ = [1 − *c*]*h*_incidental_ + *ch*_composite_, where *c* is a real number satisfying 0 ≤ *c* *≤* 1 (representing a measure for the fraction of molecules that became located within the 240-nm range and formed a dimer).

However, due to the insufficient time resolution (33 ms per frame) and limited signal-to-noise ratios in our single-molecule imaging movies, the histogram *h*_observed_ would not allow determinations of *c*, *h*_incidental_, and *h*_composite_, but appeared like a single exponential function, characterized by a single exponential decay constant between 164 and 335 ms (Figs. 3d and 4d, Supplementary Fig. 5b). The lifetime obtained in this way (for *h*_observed_) was considered sufficiently long as compared with the lifetime obtained by approximating *h*_incidental_ as a single exponential function (49 and 55 ms; Figs. 3d and 4d, Supplementary Fig. 5b; again probably due to the lack of sufficient time resolutions and limited signal-to-noise ratios of our single-molecule imaging movies to determine *h*_incidental_). Furthermore, *h*_observed_ would not depend on the number densities of AMPAR subunits: *h*_incidental_ and *h*_composite_ would not be affected by the number density, and *c* would also be independent of the number density because the true binding of two molecules only occurs after they enter the colocalization range of 240 nm.

Since the exponential decay constants for *h*_observed_ (between 164 and 335 ms) were considered sufficiently long as compared with those for *h*_incidental_ (between 49 and 55 ms), we did not attempt to make any corrections. In addition, at the ranges of the number densities of molecules employed here, the monomer reference molecules did not exhibit apparent trimers and tetramers, and therefore, we did not have to consider *h*_incidental_ (c ≈ 1).

### Estimation of diffusion coefficients and step sizes

Diffusion coefficients for individual fluorescent spots were obtained as follows^68,69^. Briefly, the single-molecule mean-square displacement (MSD for the time interval Δ*t*; i.e., Δ*r*(Δ*t*)^2^) is defined as follows. For a single-molecule trajectory consisting of *N* determined coordinates (*x*-, *y*-positions) in a two-dimensional plane, all of the [*N* − *n* + 1] partial trajectories of *n* consecutive positions (*n* ≤ *N*) were extracted. The MSD(*N*, *n*) was then calculated by averaging the square displacements of *n* steps for all of these [*N* − *n* + 1] partial trajectories and, by varying *n*, the plot of MSD (Δ*t*_n_ = *nδt*) vs. *nδt* (*δt* = the duration of each image frame) was obtained. Namely, the MSD for every time interval was calculated according to the following formula:

$${\mathrm{MSD}}(\Delta t_{\mathrm{n}}) = {\mathrm{MSD}}(n\Delta t) = {\mathrm{MSD}}_X(n\Delta t) + {\mathrm{MSD}}_Y(n\Delta t)\\ = \frac{1}{{N - n + 1}}\mathop {\sum}\limits{_{j = 1}^{N - n + 1}} {\left\{ {\left[ {x\left( {j\delta t + n\delta t} \right) - x(j\delta t)} \right]^2 \, + \,\left[ {y\left( {jdt + ndt} \right) - x(jdt)} \right]^2} \right\}}$$

where *δt* is the frame time, *x*(*jδt* *+* *nδt*) and *y*(*jδt* *+* *nδt*) describe the particle position following a time interval, Δ*t*_n_ = *nδt*, after starting at position (*x*(*jδt*), *y*(*jδt*)), *N* is the total number of frames in the recording sequence, and *n* and *j* are positive integers (*n* determines the time increment). The diffusion coefficient of a particle in the time scale of 200 ms (*D*_200ms_) was obtained by linearly fitting its single-molecule MSD-Δ*t* plot at times 167, 200, and 233 ms (the slope divided by 4 gives the diffusion coefficient). The step-size of a fluorescence particle was obtained as the distance traveled by the particle between two consecutive frames. It is useful when the trajectory of a particle (for example, transient dimers and tetramers) is so short that the error for estimating *D*_200 ms_ of the particle can be quite large.

### Reporting summary

Further information on research design is available in the Nature Research Reporting Summary linked to this article.

## Supplementary information

Supplementary Information

Peer Review

Reporting Summary

Description of Additional Supplementary Files

Supplementary Movie 1

Supplementary Movie 2

Supplementary Movie 3

## Data Availability

The data that support the findings of this study are available from the corresponding authors upon reasonable request.

## Code availability

Custom-written computer codes for data collection and analysis are available from the corresponding authors on request.
