# Supplementary material for: AMPA receptors in the synapse turnover by monomer diffusion
Source: Nat Commun. 2019 Nov 20;10:5245. doi: 10.1038/s41467-019-13229-8 (PMC6868016; doi:10.1038/s41467-019-13229-8)
Supplement: Supplementary file 1 — Supplementary Information [file 41467_2019_13229_MOESM1_ESM.pdf]

AMPA receptors in the synapse turnover by monomer diffusion

Jyoji Morise, Kenichi G.N. Suzuki, Ayaka Kitagawa, Yoshihiko Wakazono, Kogo Takamiya, Taka A. Tsunoyama, Yuri L. Nemoto, Hiromu Takematsu, Akihiro Kusumi, and Shogo Oka

Supplementary Information

- Supplementary Tables 2
- Supplementary Figures 16
- Supplementary References 53

Supplementary Tables

Supplementary Table 1. Detailed data supplementing the results shown in Fig. 1b, c and Supplementary Fig. 3b, listing the fractions of monomers, dimers, trimers, and tetramers of ACP-TM, GluA1, and GluA2, as well as the statistical parameters.

Molecule	Density ± SE (copies μm^{-2})	Fraction ± SE (%)				No. of spots	No. of cells	Degrees of freedom	Fig.
		Monomer	Dimer	Trimer	Tetramer				
ACP-TM	0.27 ± 0.011	80.8 ± 3.6	19.2 ± 3.6	-	-	779	4	25	1b
	0.43 ± 0.020	72.4 ± 2.2	27.6 ± 2.2	-	-	1,729	5	40	
	0.76 ± 0.069	71.5 ± 2.5	28.5 ± 2.5	-	-	2,192	4	41	
ACP-GluA1	0.32 ± 0.047	68.1 ± 3.2	21.9 ± 2.1	6.7 ± 4.1	3.3 ± 5.2	4,305	5	80	1c
	0.63 ± 0.033	48.8 ± 1.3	34.0 ± 1.3	5.4 ± 2.4	11.8 ± 2.9	7,730	7	77	
	1.03 ± 0.035	33.8 ± 1.4	41.5 ± 2.1	2.8 ± 3.5	21.9 ± 4.0	7,107	4	89	
ACP-GluA2	0.18 ± 0.038	56.2 ± 9.7	25.6 ± 7.8	11.6 ± 15.2	6.6 ± 18.8	1,256	4	66	S3b
	0.54 ± 0.039	42.4 ± 3.3	27.9 ± 3.5	19.2 ± 6.8	10.5 ± 8.2	6,697	10	66	
	1.01 ± 0.033	28.5 ± 1.5	30.8 ± 2.2	14.4 ± 4.2	26.3 ± 4.6	4,865	6	64	

Supplementary Table 2. Detailed data supplementing the results shown in Fig. 2a, b, d and Supplementary Figs. 4a and 5a, listing the fractions of monomers, dimers, trimers, and tetramers and the tetramer number densities of ACP-GluA1, ACP-GluA2, Halo7-GluA1, and ACP-GluA1ΔNTD, as well as the statistical parameters.

(a) ACP-GluA1

Molecule	Number density (copies μm^{-2})	Fraction ± SE (%)				Tetramer density (number μm^{-2})± SE	No. of spots	Degrees of freedom	Fig.
		Monomer	Dimer	Trimer	Tetramer				
ACP-GluA1	0.20	92.2 ± 18.4	0 ± 10.2	7.8 ± 18.4	0 ± 26.0	0 ± 0.013	802	39	
	0.24	69.1 ± 6.8	7.4 ± 5.8	23.5 ± 9.2	0 ± 14.6	0 ± 0.0086	399	37	
	0.30	85.1 ± 8.4	1.7 ± 4.4	7.8 ± 8.8	5.4 ± 11.0	0.0041 ± 0.0083	1,149	74	
	0.41	53.5 ± 3.3	38.9 ± 3.0	0 ± 5.5	7.6 ± 6.5	0.0077 ± 0.0066	991	73	
	0.45	42.7 ± 3.1	48.2 ± 4.0	0 ± 6.3	9.1 ± 7.7	0.010 ± 0.0086	964	81	
	0.51	52.2 ± 4.7	27.8 ± 4.1	0.8 ± 7.9	19.2 ± 9.1	0.025 ± 0.012	665	52	
	0.58	42.8 ± 4.1	48.0 ± 3.9	0 ± 6.2	9.2 ± 7.2	0.013 ± 0.011	1,415	66	
	0.59	38.7 ± 2.5	30.3 ± 2.9	22.4 ± 5.7	8.6 ± 6.8	0.013 ± 0.010	775	69	2a
	0.64	57.3 ± 3.4	18.3 ± 2.6	17.1 ± 5.3	7.3 ± 6.5	0.012 ± 0.010	999	63	2d
	0.66	51.5 ± 2.4	27.0 ± 2.1	16.3 ± 4.2	5.2 ± 5.2	0.0086 ± 0.0085	1,035	49	
	0.67	45.9 ± 4.6	40.3 ± 3.6	0 ± 6.5	13.8 ± 7.7	0.023 ± 0.013	521	65	
	0.79	51.2 ± 1.6	35.0 ± 1.5	1.0 ± 2.7	12.8 ± 3.3	0.025 ± 0.0064	2,320	77	
	0.93	48.5 ± 2.8	41.1 ± 2.1	0 ± 3.7	10.4 ± 4.6	0.024 ± 0.011	1,614	89	
	1.03	34.6 ± 3.3	44.6 ± 3.6	0 ± 6.1	20.8 ± 7.0	0.059 ± 0.020	1,474	79	
	1.06	31.2 ± 1.9	43.4 ± 3.0	3.6 ± 5.0	21.8 ± 5.7	0.063 ± 0.016	1,527	68	
	1.09	27.1 ± 1.4	38.4 ± 2.3	6.7 ± 4.0	27.8 ± 4.4	0.090 ± 0.014	2,492	88	

(b) ACP-GluA2

Molecule	Number density (copies μm^{-2})	Fraction ± SE (%)				Tetramer density (number μm^{-2})± SE	No. of spots	Degrees of freedom	Fig.
		Monomer	Dimer	Trimer	Tetramer				
ACP-GluA2	0.11	67.1 ± 24.2	20.9 ± 22.1	12.0 ± 36.9	0 ± 52.9	0 ± 0.015	113	27	
	0.15	60.7 ± 7.2	28.5 ± 7.6	10.8 ± 12.1	0 ± 17.2	0 ± 0.0065	388	36	
	0.19	61.9 ± 13.9	16.6 ± 9.8	18.3 ± 20.1	3.2 ± 25.2	0.0015 ± 0.012	300	54	
	0.29	43.7 ± 6.4	27.6 ± 6.5	7.4 ± 12.5	21.3 ± 14.2	0.015 ± 0.010	455	65	
	0.39	40.5 ± 3.4	26.8 ± 3.8	16.1 ± 7.3	16.6 ± 8.4	0.016 ± 0.0082	516	61	
	0.41	37.4 ± 2.8	36.8 ± 3.5	24.4 ± 6.4	1.4 ± 8.0	0.0015 ± 0.0082	507	60	
	0.42	35.4 ± 5.3	50.1 ± 8.2	11.2 ± 12.6	3.3 ± 16.0	0.0035 ± 0.017	472	48	
	0.46	48.2 ± 5.0	25.6 ± 4.7	16.1 ± 9.2	10.1 ± 11.1	0.012 ± 0.013	707	65	
	0.50	41.2 ± 3.5	26.1 ± 3.8	22.2 ± 7.5	10.5 ± 8.9	0.013 ± 0.011	1,097	65	
	0.61	43.7 ± 4.4	25.3 ± 4.4	13.5 ± 8.7	17.5 ± 10.3	0.027 ± 0.016	882	62	2d
	0.61	42.2 ± 3.9	25.8 ± 4.1	26.0 ± 8.2	6.1 ± 9.9	0.0092 ± 0.015	722	57	S4a
	0.62	53.8 ± 7.6	16.8 ± 6.1	24.9 ± 4.5	4.5 ± 15.7	0.0070 ± 0.024	769	64	
	0.70	43.2 ± 4.2	20.9 ± 4.2	12.3 ± 8.4	23.6 ± 9.3	0.041 ± 0.016	524	62	
	0.72	33.4 ± 3.2	36.7 ± 4.5	21.3 ± 8.1	8.6 ± 9.8	0.015 ± 0.018	501	62	
	0.87	40.0 ± 4.0	20.2 ± 4.3	11.3 ± 8.6	28.5 ± 9.3	0.062 ± 0.020	879	61	
	0.95	30.8 ± 2.3	39.2 ± 3.5	20.2 ± 6.2	9.7 ± 7.4	0.023 ± 0.018	602	59	
	1.00	31.7 ± 2.0	25.5 ± 2.6	19.2 ± 5.2	23.6 ± 5.7	0.059 ± 0.014	1,117	63	
	1.04	18.9 ± 1.2	34.1 ± 2.5	15.5 ± 4.5	31.5 ± 4.8	0.082 ± 0.012	790	57	
	1.06	28.6 ± 1.8	32.0 ± 2.8	8.2 ± 5.2	31.2 ± 5.5	0.083 ± 0.015	814	63	
	1.10	21.7 ± 1.6	38.5 ± 3.2	11.5 ± 5.6	28.3 ± 6.1	0.078 ± 0.017	663	61	

(c) Halo7-GluA1

Molecule	Number density (copies μm^{-2})	Fraction ± SE (%)				Tetramer density (number μm^{-2})± SE	No. of spots	Degrees of freedom	Fig.
		Monomer	Dimer	Trimer	Tetramer				
Halo7-GluA1	0.11	60.4 ± 7.5	0 ± 6.4	39.6 ± 13.6	0 ± 16.3	0 ± 0.0045	290	42	2d S5a
	0.48	35.5 ± 3.6	42.5 ± 5.2	16.3 ± 8.7	5.7 ± 10.8	0.0069 ± 0.013	475	50	
	0.60	37.8 ± 2.5	29.2 ± 3.0	21.7 ± 5.8	11.3 ± 6.8	0.017 ± 0.010	1,502	53	
	0.68	31.3 ± 1.5	28.2 ± 2.0	23.5 ± 4.0	17.0 ± 4.5	0.029 ± 0.0076	2,848	54	
	0.71	33.0 ± 2.2	30.9 ± 2.9	23.3 ± 5.6	12.8 ± 6.5	0.023 ± 0.012	2,281	49	
	0.74	39.2 ± 2.0	25.0 ± 2.2	18.7 ± 4.3	17.1 ± 4.9	0.032 ± 0.0092	1,107	55	
	0.79	28.9 ± 2.5	25.8 ± 3.5	35.6 ± 7.3	9.7 ± 8.4	0.019 ± 0.017	1,632	52	
	0.88	22.8 ± 1.0	44.8 ± 2.0	21.9 ± 3.3	10.5 ± 3.9	0.023 ± 0.0086	2,657	52	
	1.02	42.2 ± 4.6	39.7 ± 5.5	5.1 ± 9.4	13.0 ± 11.2	0.033 ± 0.029	868	46	
	1.10	17.3 ± 0.6	26.5 ± 1.2	22.0 ± 2.5	34.2 ± 2.5	0.094 ± 0.0069	2,359	56	

(d) ACP-GluA1ΔNTD

Molecule	Number density (copies μm^{-2})	Fraction ± SE (%)				Tetramer density (number μm^{-2})± SE	No. of spots	Degrees of freedom	Fig.
		Monomer	Dimer	Trimer	Tetramer				
ACP-GluA1 ΔNTD	0.11	81.5 ± 16.2	0 ± 10.2	18.5 ± 16.2	0 ± 26.0	0 ± 0.0069	308	35	2b 2d
	0.16	73.1 ± 17.3	17.6 ± 14.3	9.3 ± 24.8	0 ± 34.8	0 ± 0.014	267	33	
	0.18	70.1 ± 10.1	28.2 ± 8.6	1.7 ± 16.1	0 ± 21.3	0 ± 0.0093	207	26	
	0.30	75.7 ± 21.4	20.7 ± 8.2	0 ± 18.7	3.6 ± 23.4	0.0027 ± 0.018	507	39	
	0.46	39.8 ± 4.4	26.1 ± 4.9	28.0 ± 9.7	6.1 ± 11.9	0.0070 ± 0.014	467	48	
	0.50	73.0 ± 5.3	15.5 ± 4.3	11.5 ± 7.4	0 ± 10.6	0 ± 0.013	689	48	
	0.54	79.2 ± 16.2	12.5 ± 8.9	4.3 ± 18.1	4.0 ± 23.0	0.0054 ± 0.031	956	46	
	0.60	49.5 ± 7.3	31.6 ± 7.0	4.0 ± 12.9	14.9 ± 15.2	0.022 ± 0.023	649	47	
	0.82	55.4 ± 6.0	28.1 ± 5.0	13.0 ± 9.6	3.5 ± 12.1	0.0072 ± 0.025	815	54	
	0.92	58.1 ± 6.3	14.6 ± 4.7	19.7 ± 9.7	7.6 ± 11.8	0.018 ± 0.027	1,446	50	

Supplementary Table 3. Detailed data supplementing the results shown in Fig. 2c and Supplementary Fig. 4b (fractions of monomers and oligomers), listing the fractions of monomers, dimers, trimers, and tetramers of ACP-GluA1 and ACP-GluA2, before and after the additions of the agonists, 0.1 mM AMPA and 10 mM L-glutamate, along with their statistical parameters.

Molecule	Agonist	Fraction ± SE (%)				No. of spots	No. of cells	Degrees of freedom	Fig.
		Monomer	Dimer	Trimer	Tetramer				
ACP-GluA1[a]	-	35.9 ± 1.7	40.4 ± 2.2	9.9 ± 3.7	13.8 ± 4.4	2,579	7	49	2c
	AMPA	40.5 ± 2.9	44.5 ± 3.7	5.6 ± 5.8	9.4 ± 7.5	1,930	5	51	
	L-glutamate	38.6 ± 3.0	40.6 ± 2.6	6.5 ± 6.6	11.1 ± 7.9	1,631	5	49	
ACP-GluA2[a]	-	36.6 ± 1.9	50.5 ± 2.9	3.9 ± 4.4	9.0 ± 5.4	4,276	8	51	S4b
	AMPA	36.3 ± 2.8	44.7 ± 3.7	10.1 ± 6.0	8.9 ± 7.3	2,612	7	47	
	L-glutamate	38.0 ± 2.7	46.4 ± 3.8	1.3 ± 6.1	14.3 ± 7.2	3,182	7	48	

[a]Average number densities of ACP-GluA1 and ACP-GluA2 expressed in the HEK293-TM were 0.50 ± 0.09 and 0.43 ± 0.024 copies μm^{-2} , respectively.

Supplementary Table 4. Numbers of examined colocalization/oligomerization events, numbers of examined cells, and degrees of freedom for evaluating the lifetimes of homo- and hetero-dimers, trimers, and tetramers, of fluorescently-labeled tagged GluA1, GluA1ΔNTD, GluA2, and TM, the fluorescent probes used, and the temperatures employed, supplementing the results shown in Table 1 and other figures.

Molecules	Colocalizations (Oligomers)	No. of examined events	No. of examined cells	Degrees of freedom	Label	Temp. (°C)	Fig.
ACP-TM (Monomer reference)	Incidental colocalization	231	12	9	ATTO594	22	3d, S8d
Halo7-TM (Monomer reference)	Incidental colocalization	146	12	11	Rho110[a]	22	S5b, S8e
ACP-TM Halo7-TM (Monomer reference)	Incidental colocalization	115	8	5	ATTO594 Rho110	22	4d, S9a
ACP-GluA1	GluA1 homo-D[b]	236	17	15	ATTO594	37	3d, 3e, 5a
	GluA1 homo-Tri[b]	54	25	9			None
	GluA1 homo-T[b]	90	17	9			3e, 4e
ACP-GluA1ΔNTD	GluA1ΔNTD homo-D	211	31	10	ATTO594	37	3d
	GluA1ΔNTD homo-T	89	31	2			3e
Halo7-GluA1	GluA1 homo-D	202	17	16	Rho110	37	S5b
ACP-GluA1 Halo7-GluA1	GluA1 homo-D	227	6	18	ATTO594 Rho110	37	4d, S9b
ACP-GluA2	GluA2 homo-D	137	17	21	ATTO594	37	S8d, S10a
	GluA1 homo-Tri	64	13	13			None
	GluA2 homo-T	80	17	11			4e, S8d
Halo7-GluA2	GluA2 homo-D	216	6	18	Rho110	37	S8e, S8f
Halo7-GluA2 in CHO-K1 cells[c]	GluA2 homo-D	158	6	23	Rho110	37	S8f
ACP-GluA2 Halo7-GluA2	GluA2 homo-D	243	8	25	ATTO594 Rho110	37	4d, S9b
ACP/Halo7-GluA1 Halo7/ACP-GluA2	GluA1/A2 hetero-D	359	16	39	ATTO594 Rho110	37	4d
	GluA1/A2 hetero-T	200	16	24			4e

[a]Rhodamine110.

[b]D, Tri, and T represent dimers, trimers, and tetramers, respectively.

[c]Only the results shown in this line were obtained in CHO-K1 cells, for comparison with the data obtained in HEK293 cells (all other results shown here).

Supplementary Table 5. Summary of the diffusion coefficients of fluorescently-labeled, tagged molecules of GluA1, GluA1ΔNTD, GluA2, and TM in the HEK293-PM, along with the statistical parameters for their determinations, supplementing the results shown in Supplementary Fig. 7. Observation temperatures were 37°C, except for ACP- and Halo7-TM, which were observed at both 22°C and 37°C.

Molecules (monomers)	Median D_{200ms} (μm^2 s^{-1})	P value (Brunner-Munzel test)	D_{200ms} (Mean ± SEM) from ensemble-averaged MSD (μm^2 s^{-1})	No. of molecules	No. of cells	Fluorescent probe
ACP-TM	0.23$^{*1\ a}$	-	0.23 ± 0.0021	1,787	3	ATTO594
ACP-TM at 22°C	0.13^{*2,Y1}	< 2.2 x 10^{-16}	0.13 ± 0.0026	207	6	ATTO594
Halo7-TM	0.22^{*3}	-	0.25 ± 0.00095	499	12	Rho110
Halo7-TM at 22°C	0.14^{*4,Y3}	< 2.2 x 10^{-16}	0.13 ± 0.00042	2,375	5	Rho110
ACP-GluA1 Monomer	0.15^{*5,N2}	0.68	0.15 ± 0.00049	92	14	ATTO594
ACP-GluA1ΔNTD Monomer	0.15^{N2,N5}	^{N2}0.26<br>^{N5}0.23	0.15 ± 0.0012	131	9	ATTO594
Halo7-GluA1 Monomer	0.15^{*6,N4,N5}	^{N4}0.13<br>^{N5}0.30	0.15 ± 0.00075	136	19	Rho110
GluA1-mGFP Monomer	0.16^{N5,N6}	^{N5}0.38<br>^{N6}0.81	0.14 ± 0.0015	87	9	mGFP
ACP-GluA2 Monomer	0.16^{*7,N2}	5.1 x 10^{-2}	0.18 ± 0.00023	132	20	ATTO594
Halo7-GluA2 Monomer	0.15^{*8,N4,N7}	^{N4}0.26<br>^{N7}0.21	0.13 ± 0.0024	118	9	Rho110
GluA2-mGFP Monomer	0.14^{N7,N8}	^{N7}0.14<br>^{N8}0.58	0.14 ± 0.0012	68	9	mGFP

^{a} $*$, ^{Y}, and ^{N} indicate the results of the statistical tests. The distribution selected as the basis for the comparison is shown by the superscript, $*$. Different numbers (1–8) indicate different bases. The superscript ^{Y} or ^{N} indicates that the distribution is or is not significantly different from that of the basis distribution, based on the p value of the Brunner-Munzel test (the next column on the right) being smaller or greater than 0.05, respectively.

Supplementary Table 6. Summary of the lifetimes of hetero-dimers (hetero-D) and tetramers (hetero-T) of GluA1 and GluA2 (after the corrections for the photobleaching lifetimes) in the HEK293-PM at 37°C, together with statistical parameters, supplementing the data shown in Table 1 and Supplementary Fig. 9c, d.

Molecules[a]	Hetero-D or T	Heteromer lifetimes (Mean ± SEM) (ms)[c]	*P* value (Log-rank test)	No. of examined events	No. of examined cells	Degrees of freedom
ACP-GluA1 Halo7-GluA2	Hetero-D[b]	347 ± 33[*1]	-	236	8	39
	Hetero-T[b]	218 ± 14[*2,Y1]	5.7×10^{-3}	128	8	24
ACP-GluA2 Halo7-GluA1	Hetero-D	312 ± 37[*3,N1]	0.39	123	8	30
	Hetero-T	192 ± 14 [N2,Y3]	[N2]0.14<br>[Y3]1.6×10^{-3}	72	8	16

[a]ACP and Halo7 tags were labeled with ATTO594 and Rhodamine110, respectively.

[b]D and T represent dimers and tetramers, respectively.

[c] *, [Y], and [N] indicate the results of the statistical tests, as described in note b in **Table 1**.

Supplementary Table 7. Summary of the lifetimes of homodimers of GluA1 and GluA2 in the presence and absence of overexpressed TARP2-mGFP, as well as the lifetimes of heteromers of GluA1 and TARP2-mGFP and those of GluA2 and TARP2-mGFP (for monomers and homodimers of GluA1 or GluA2) after the correction for photobleaching lifetimes, together with statistical parameters, supplementing the data shown in Fig. 5a, c and Supplementary Fig. 10a, c.

Molecules[a]	Colocalizations (Oligomers)	Lifetimes[b] (Mean ± SEM) (ms)	*P* value (Log-rank test)	No. of examined events	No. of examined cells	Degrees of freedom
ACP-GluA1	Homodimer	164 ± 16[*1]	-	236	17	15
ACP-GluA1 with overexpressed TARP2-mGFP	Homodimer	158 ± 16[N1]	0.31	177	13	19
ACP-GluA1 monomer and TARP2-mGFP	Heteromer	190 ± 7[*2]	-	243	15	25
ACP-GluA1 dimer and TARP2-mGFP	Heteromer	184 ± 8[*3,N2]	0.96	115	15	20
ACP-GluA2	Homodimer	156 ± 16[*4]	-	137	17	21
ACP-GluA2 with overexpressed TARP2-mGFP	Homodimer	160 ± 13[N4]	0.52	157	12	19
ACP-GluA2 monomer and TARP2-mGFP	Heteromer	229 ± 10[*5,N2]	8.1×10^{-2}	249	16	27
ACP-GluA2 dimer and TARP2-mGFP	Heteromer	206 ± 10[N3,N5]	[N3]0.91<br>[N5]0.12	136	16	22

[a]ACP tag was labeled with ATTO594.

[b] * and [N] indicate the results of the statistical tests, as described in note b in **Table 1**.

Supplementary Table 8. Numbers of examined cells expressing Halo7-GluA1 and ACP-GluA1ΔNTD (ATTO594 label) at various levels in the HEK293-PM, for observing the relationship of the cytoplasmic Ca^{2+} increases with the number densities of expressed molecules in the PM, as well as with the number densities of tetramers in the PM, supplementing the results shown in Fig. 6c, d.

(a) For the data shown in Fig. 6c; for observing the relationship of the cytoplasmic Ca^{2+} increases with the number densities of expressed molecules in the PM

Range of number density (copies μm^{-2})	No. of examined cells (Halo7-GluA1)	No. of examined cells (ACP-GluA1ΔNTD)
0.00 - 0.33	1	5
0.33 - 0.67	4	5
0.67 - 1.00	10	4
1.00 - 1.33	9	5
1.33 - 1.67	6	2
1.67 - 2.00	4	5
2.00 - 2.33	1	5
2.33 - 2.67	1	4
2.67 - 3.00	0	1
3.33 - 3.66	0	1
5.00 - 5.33	0	1

(b) For the data shown in Fig. 6d; for observing the relationship of the cytoplasmic Ca^{2+} increases with the number densities of tetramers in the PM

Range of tetramer density (numbers μm^{-2})	No. of observed cells (Halo7-GluA1)	No. of observed cells (ACP-GluA1ΔNTD)
0.000 - 0.033	11	20
0.033 - 0.067	9	11
0.067 - 0.100	3	4
0.100 - 0.133	2	1
0.133 - 0.167	5	1
0.167 - 0.200	2	0
0.233 - 0.267	2	0
0.300 - 0.333	1	1
0.400 - 0.433	1	0

Supplementary Table 9. Diffusion coefficients (D_{200ms}) of Cy3-DOPE and Halo7(ATTO594)-CD47 in the HEK293-PM and dendritic-shaft PM observed at 37°C, along with their statistical parameters, supplementing the data shown in Supplementary Fig. 11a.

Molecules	Cell	Median D_{200ms} ($\mu m^2\ s^{-1}$)	*P* value (Brunner-Munzel test)	D_{200ms} (Mean ± SEM) from ensemble-averaged MSD ($\mu m^2\ s^{-1}$)	No. of examined molecules	No. of examined cells
Cy3-DOPE	HEK293	$0.25^{*1\ a}$	-	0.25 ± 0.0023	281	20
Cy3-DOPE	Neuron (Dendritic-shaft)[b]	0.16^{Y1}	2.2×10^{-16}	0.19 ± 0.00062	205	19
Halo7-CD47	HEK293	0.22^{*2}	-	0.21 ± 0.00077	2,285	22
Halo7-CD47	Neuron (Dendritic-shaft)[c]	0.14^{Y2}	$< 2.2 \times 10^{-16}$	0.15 ± 0.0017	307	7

[a] * and ^{Y} indicate the results of the statistical tests, as described in note a in **Supplementary Table 5**.

[b]DIV24.

[c]DIV15.

Supplementary Table 10. Diffusion coefficients of ATTO594-labeled ACP-GluA1 and ACP-GluA2 in the HEK293-PM, ATTO594-labeled Halo7-GluA1, Halo7-GluA1ΔNTD, and Halo7-GluA2 in the dendritic-shaft PM and on Homer1b, and endogenous GluA1 and GluA2 conjugated with their respective mAbs-ATTO594 in the dendritic-shaft PM at 37°C, along with their statistical parameters, supplementing the diffusion data shown in Fig. 7c, d, as well as in Supplementary Figs. 7b–d, 11b, c, 12c, d, 15c, and 16c.

Molecules	Cell	Median D_{200ms} ($\mu m^2 s^{-1}$)	*P* value (Brunner-Munzel test)	D_{200ms} (Mean ± SEM) from ensemble-averaged MSD ($\mu m^2 s^{-1}$)	No. of examined molecules	No. of examined cells	Fig.
ACP-GluA1 Monomer	HEK293	0.15[*1 a]	-	0.15 ± 0.00049	92	14	7c, S7b S7d,S11b
ACP-GluA1 Homo-D[b]	HEK293	0.046[*2,Y1]	6.7×10^{-12}	0.069 ± 0.0014	104	19	7c, S7b, S11b
ACP-GluA1 Homo-T[b]	HEK293	0.014[*3,Y2]	1.6×10^{-3}	0.014 ± 0.0038	10	9	7c, S7b, S11b
Halo7-GluA1	Neuron (Dendritic shaft)[c]	0.073[*4,Y2,Y3]	[Y2] 1.3×10^{-3}<br>[Y3] 4.0×10^{-5}	0.090 ± 0.0015	1,250	34	7c, 7d, S15c
Halo7-GluA1	Neuron (Synapse)[c]	-	-	0.010 ± 0.00078	127	27	7d
Halo7-GluA1ΔNTD	Neuron (Dendritic shaft)[c]	0.087[Y4]	1.7×10^{-3}	0.11 ± 0.00057	527	27	7c
GluA1 (mAb)	Neuron (Dendritic shaft)[c]	0.079[N4]	0.63	0.068 ± 0.0022	124	20	S15c
ACP-GluA2 Monomer	HEK293	0.16[*5]	-	0.18 ± 0.00023	132	20	S7c, S7d, S11c, S12c
ACP-GluA2 Homo-D	HEK293	0.043[*6,Y5]	$< 2.2 \times 10^{-16}$	0.051 ± 0.0011	132	20	S7c, S11c, S12c
ACP-GluA2 Homo-T	HEK293	0.016[*7,Y6]	6.3×10^{-3}	0.015 ± 0.0052	15	9	S7c, S11c, S12c
Halo7-GluA2	Neuron (Dendritic shaft)[c]	0.065[*8,Y6,Y7]	[Y6] 4.6×10^{-6}<br>[Y7] 9.9×10^{-6}	0.073 ± 0.0013	901	13	S12c, S12d, S16c
Halo7-GluA2	Neuron (Synapse)[c]	-	-	0.0076 ± 0.00045	129	19	S12d
GluA2 (mAb)	Neuron (Dendritic shaft)[c]	0.073[N8]	0.58	0.054 ± 0.0044	167	22	S16c

[a] *, [Y], and [N] indicate the results of the statistical tests, as described in note a in **Supplementary Table 5**.

[b]D and T represent dimers and tetramers, respectively.

[c]DIV13.

Supplementary Figures

Supplementary Figure 1

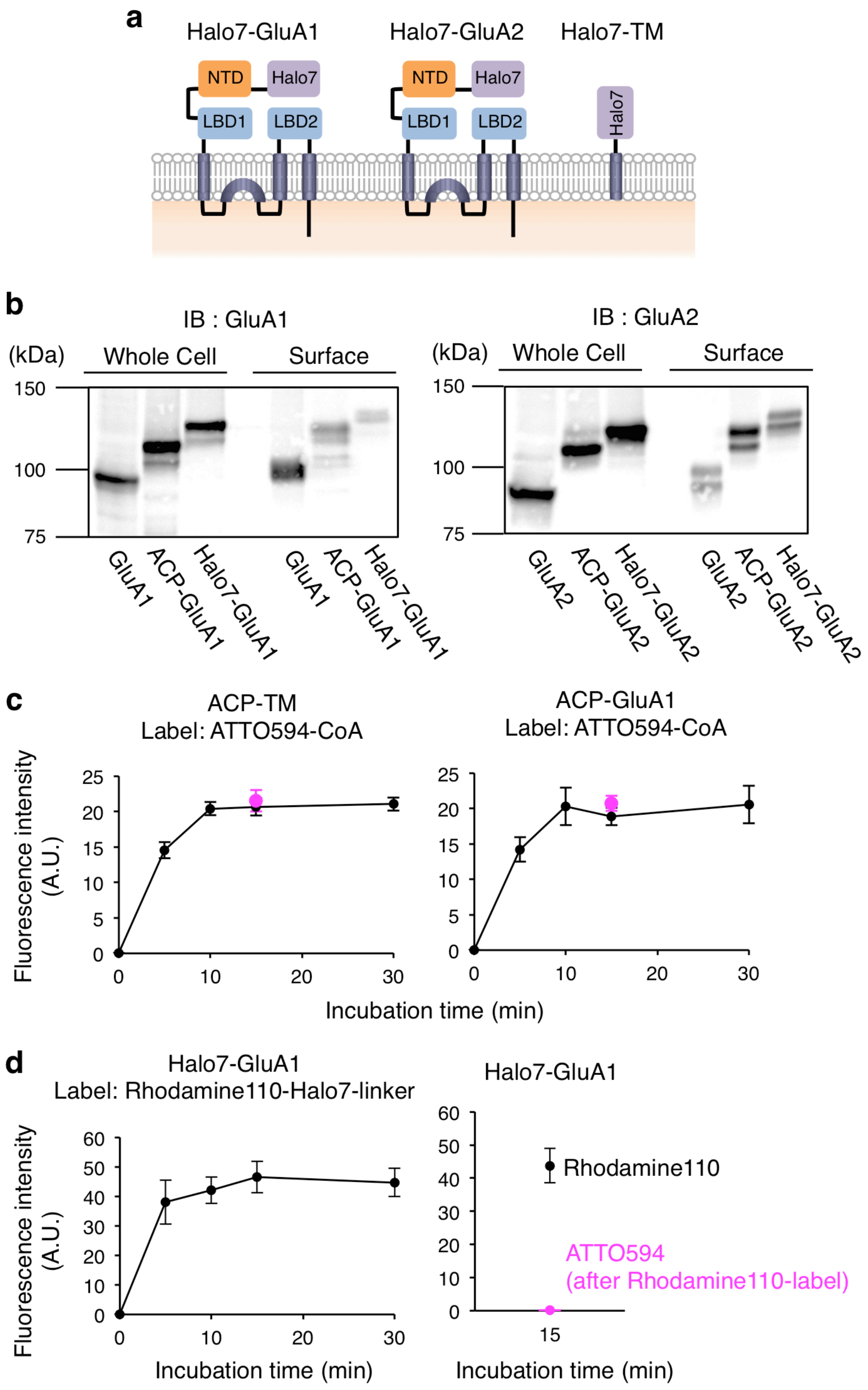

Supplementary Figure 1. ACP-GluA1, Halo7-GluA1, ACP-GluA2, and Halo7-GluA2 were expressed in the HEK293-PM and virtually every molecule was fluorescently labeled.

(**a**) Schematic figure showing the structures of Halo7-GluA1, Halo7-GluA2, and Halo7-TM.

(**b**) Results showing that GluA1, ACP-GluA1, Halo7-GluA1, GluA2, ACP-GluA2, and Halo7-GluA2 were expressed on the HEK293 cell surface, when HEK293 cells were transfected with the cDNAs encoding these proteins. After the HEK293 cells were transfected, biotinylated with a non-permeable biotin derivative, EZ-Link Sulfo-NHS-SS-biotin, and then extracted with 1% Triton X-100 and 0.5% Nonident P-40, part of the extract was immunoblotted with anti GluA1 (left) or anti GluA2 (right) rabbit polyclonal antibodies (Whole Cell). The remaining extract was treated with streptavidin beads, and biotinylated molecules were captured with the beads, and then immunoblotted in the same way (Surface). The bands were located approximately at the positions of the predicted sizes (molecular weights of non-tagged, ACP-tagged, and Halo7-tagged molecules are predicted to be approximately 100, 110 and 130 kDa, respectively). Often two bands closely located near the expected positions, rather than a single band, appeared in each lane, which might represent the molecules with immature and mature glycans[1]. These results show that some fractions of the expressed molecules are transported to the PM. For the raw images of these blots, see **Supplementary Fig. 20**.

(**c, d**) Time courses of the conjugation of fluorescent dyes to ACP-TM and ACP-GluA1 (**c**, 50 nM ATTO594-CoA and 1 μM ACP synthase) and Halo7-GluA1 (**d**, 50 nM rhodamine110–Halo7 ligand), as monitored by the fluorescence intensity. The HEK293 cells transfected with the cDNAs encoding the molecules fused with ACP- or Halo7-tag proteins were incubated with the fluorescent probes (and the enzyme in the case of ACP). Their bottom PM was observed using total internal reflection fluorescence microscopy (TIRFM) at single-molecule sensitivities, and the fluorescence signal intensity in a 9.6 × 9.6 μm membrane area was measured (keys represent mean ± SEM). After the addition of the fluorescent ligands (and the ACP synthase), the fluorescence signal intensities reached plateaus within 10 min (black keys), suggesting that virtually all ACP (Halo7) molecules were conjugated by ATTO594 (rhodamine110).

This was confirmed in the following ways. In the experiments in (**c**), which were for ACP-TM and ACP-GluA1, the ACP reaction was performed under more intensive conditions (200 nM ATTO594-CoA and 4 μM ACP synthase for 15 min, magenta key). These reactions gave virtually the same fluorescence signal intensities as those observed in cells treated under much milder reaction conditions. In the case of (**d**), which was for Halo7-GluA1, after the cells were incubated with 50 nM rhodamine110-conjugated Halo7 ligand for 15 min, the cells were further incubated with 50 nM ATTO594-conjugated Halo7 ligand. No ATTO594 signal was found (magenta key; n = 10 cells; the same 10 cells were observed before and after the incubation with ATTO594–Halo7 ligand),

indicating that all Halo7-GluA1 molecules on the cell surface had already been labeled after the first incubation with 50 nM rhodamine110 for 15 min (**d**, right).

The numbers of cells observed in other experiments were the following. Black keys in **c**: 20, 17, 21, and 19 cells (ACP-TM) and 12, 13, 25, and 10 cells (ACP-GluA1) for the incubation periods of 5, 10, 15, and 30 min, respectively. Magenta keys in **c**: 12 and 10 cells for ACP-TM and ACP-GluA1, respectively. In **d** (left), 14, 15, 20, and 16 cells for 5, 10, 15, and 30 min, respectively. Note that although we did not examine the labeling efficiencies for all of the combinations of the dye molecules (ATTO594 and rhodamine110), tag proteins (ACP and Halo7), and target molecules (GluA1, GluA2, TM), we believe that the results shown in **c** and **d** strongly suggest that all of the tagged molecules employed in this study were labeled at levels close to 100%.

Supplementary Figure 2

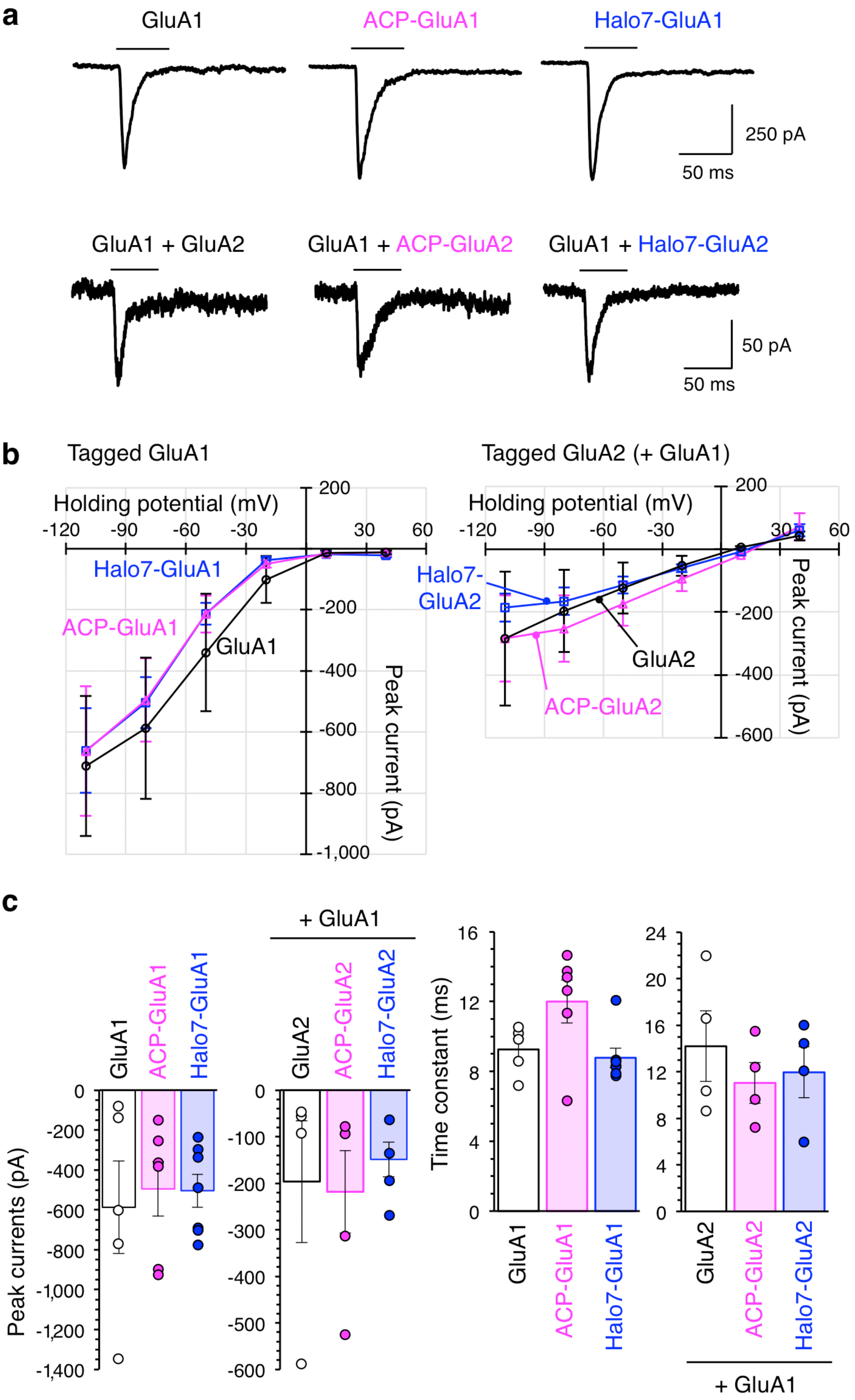

Supplementary Figure 2. Tagged GluA1 and GluA2 subunits are functional.

(**a**) Representative current traces at a clamp voltage of –80 mV observed in the HEK293 cells transfected with GluA1, ACP-GluA1, and Halo7-GluA1 (for the number of independent experiments, see **b**), and the effects of the puff application of L-glutamate (the bars above the current traces). The influence of attaching ACP- and Halo7-tags to GluA2 was analyzed with GluA1 co-expression (see the positive currents for the positive holding potential in **b**, which is a characteristic function of GluA2).

(**b**) The current–voltage (I–V) relationships (clamped voltage from –110 to +40 mV, every 30 mV) when L-glutamate was applied to HEK293 cells transfected with GluA1 (black, n = 5), ACP-GluA1 (red, n = 6), and Halo7-GluA1 (blue, n = 7) (left), and with GluA2 (black, n = 4), ACP-GluA2 (red, n = 4), and Halo7-GluA2 (blue, n = 4) (right; in the presence of coexpressed GluA1).

(**c**) Peak current amplitudes (left) and time constants (right) of L-glutamate-induced current responses at –80 mV observed in HEK293 cells expressing GluA1, ACP-GluA1, and Halo7-GluA1 (left) and GluA2, ACP-GluA2, and Halo7-GluA2 (right; with GluA1 co-expression) (for the number of independent experiments, see **b**), indicating that these expressed molecules probably form functional homotetramers or heterotetramers in the PM of HEK293 cells. Error bars: SEM. For the peak current responses observed in individual experiments at other holding voltages (Supplementary for the results shown in **b**), see **Supplementary Fig. 21**.

Supplementary Figure 3

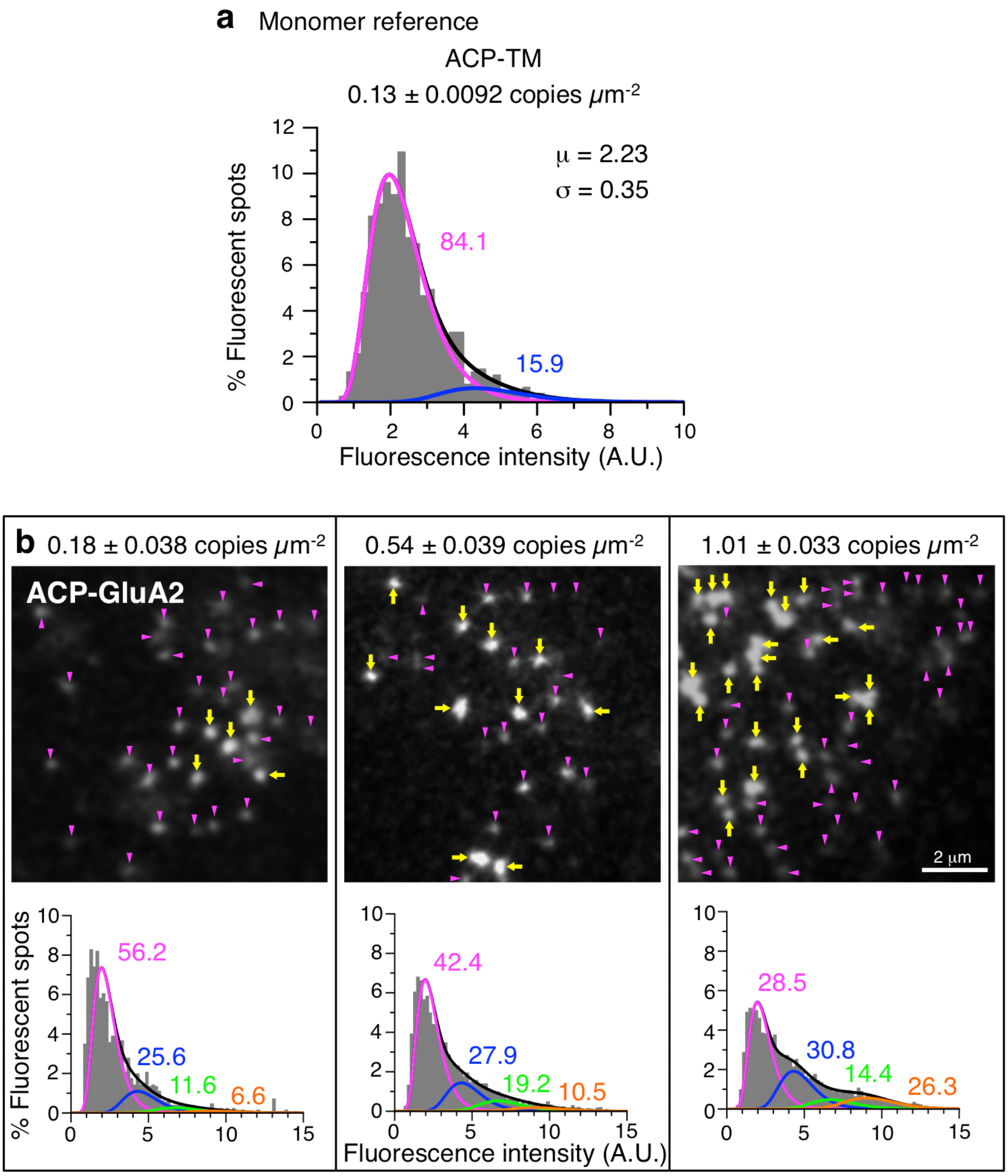

Supplementary Figure 3. The distribution of the signal intensities of individual fluorescent spots of ATTO594-labeled ACP-TM (a monomer reference molecule) expressed in the HEK293-PM at the lowest number density employed in this study, and that of ATTO594-labeled ACP-GluA2, which should be compared to the ACP-GluA1 data shown in Fig. 1.

(**a**) The distribution of the signal intensities of single individual fluorescent spots of ACP-TM at an average expression level of 0.13 ± 0.0092 copies μm^{-2}. The distribution could be fitted with the sum of two lognormal functions, representing monomers and (apparent) dimers (magenta and blue

curves, respectively, and the black curve represents the sum. Since ACP-TM exhibited a very low tendency to form homodimers[2], the dimer component is likely to represent apparent dimers due to incidental overlap of the two spots). The best-fit lognormal function for monomers provided the mean intensity (the mode of the lognormal function = 2.23 A.U.) and the standard deviation (σ = 0.35). These values were used for the lognormal fitting of the signal intensity distributions of ACP-tagged GluA1 and GluA2 molecules, using the sum of four lognormal functions (for monomers, dimers, trimers, and tetramers), to provide the fractions of monomers, dimers, trimers, and tetramers. The number of examined fluorescent spots: 749.

(**b**) ACP-GluA2 results supplementing the ACP-GluA1 data shown in **Fig. 1c**. For details of these figures, see the captions to **Fig. 1b** and **c**. Distributions of the signal intensities of single individual fluorescent spots of ACP-GluA2 were obtained at the expression levels (number densities) of 0.18 ± 0.038, 0.54 ± 0.039, and 1.01 ± 0.033 copies μm^{-2} (1,256, 6,697, and 4,865 spots in 4, 10, and 6 images [cells], respectively).

Supplementary Figure 4

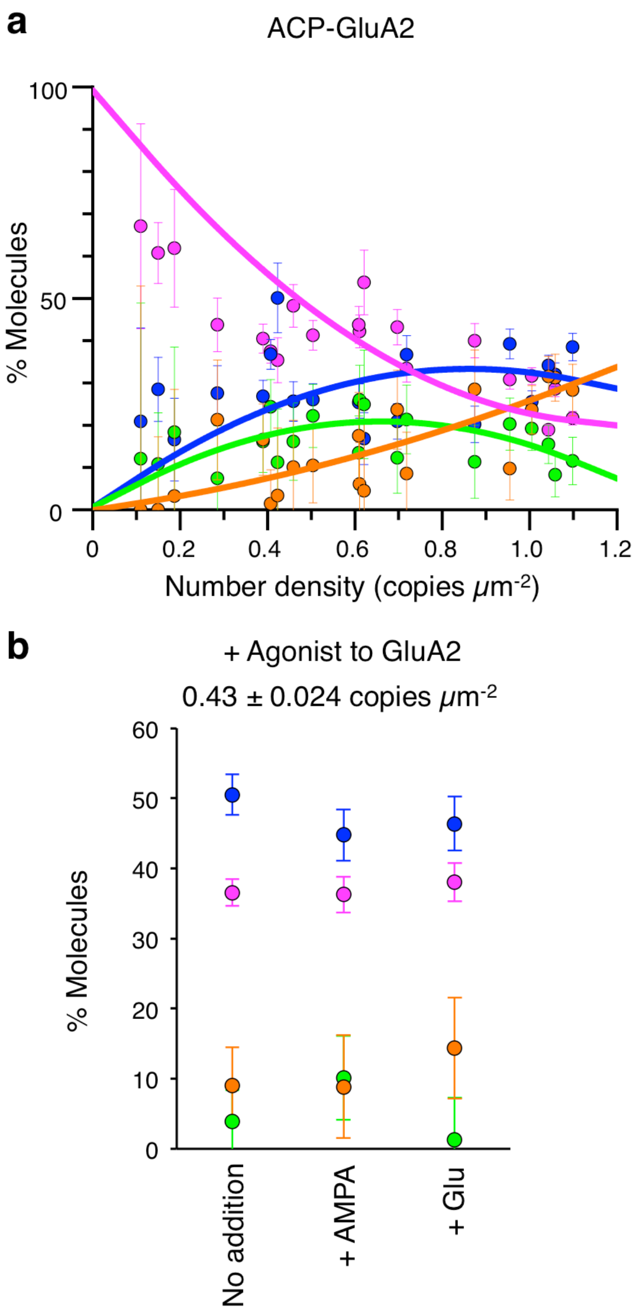

Supplementary Figure 4. With an increase in the number density of GluA2 expressed in the HEK293-PM, the GluA2 monomer fraction decreases whereas the homotetramer fraction increases.

Results obtained by using ACP-GluA2, supplementing the results of ACP-GluA1 shown in **Fig. 2a, d**. For the details of these figures, see the captions to **Fig. 2a, d**.

(**a**) The fractions of ACP-GluA2 molecules that exist as monomers, homodimers, homotrimers, and homotetramers, plotted as a function of the number density of ACP-GluA2 molecules expressed in the HEK293-cell PM (magenta, blue, green, and orange circles and lines, respectively, which are the

same for all of the panels). Curves are to help the eye (curve fitting with quadratic functions). The numbers of independent experiments conducted to obtain the results shown in this figure are summarized in **Supplementary Table 2b**.

(**b**) The effects of the agonists on the fractions of ACP-GluA2 molecules that exist as monomers, homodimers, homotrimers, and homotetramers. The numbers of cells examined in (**b**) are summarized in **Supplementary Table 3** (ACP-GluA2 expressed at 0.43 ± 0.024 copies μm^{-2}).

Supplementary Figure 5

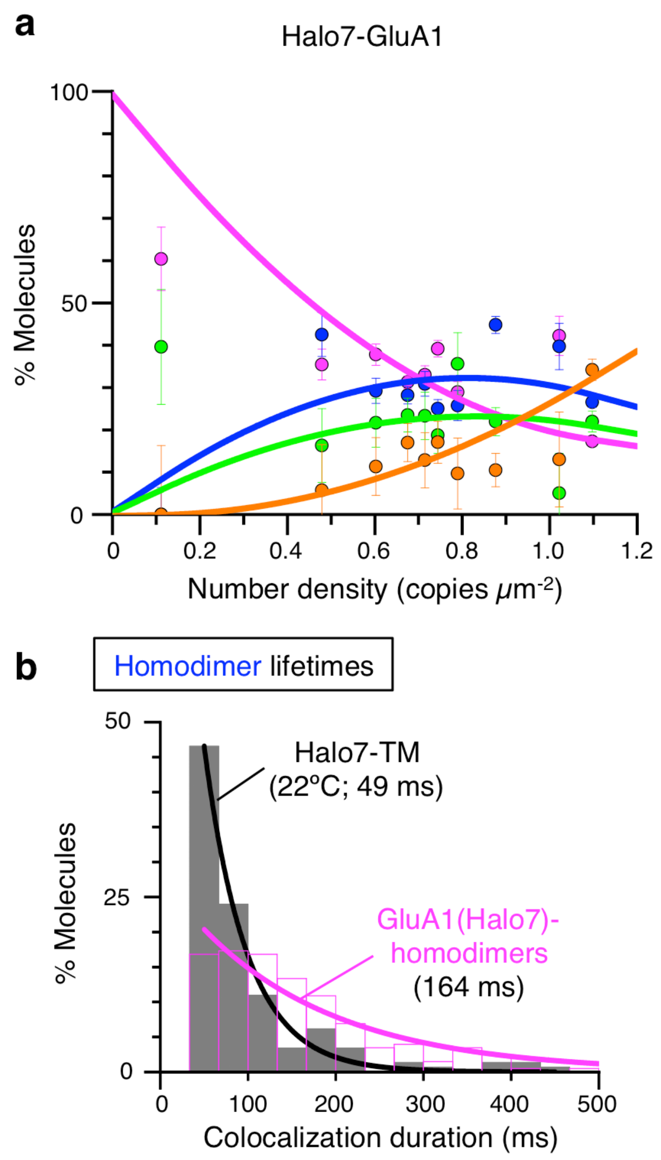

Supplementary Figure 5. With an increase in the number density of Halo7-GluA1 expressed in HEK293-PM, the Halo7-GluA1 monomer fraction decreases whereas the homotetramer fraction increases, while the Halo7-GluA1 homodimers are metastable with a lifetime of 164 ms.

Results obtained by using Halo7-GluA1, supplementing the ACP-GluA1 data shown in **Figs. 2a** and **3d**. For details of these figures, see the captions to **Figs. 2a** and **3d**. The numbers of experiments conducted to obtain the results shown in this figure are summarized in **Supplementary Tables 2c** and **4**, respectively.

(**a**) The fractions of Halo7-GluA1 molecules that exist as monomers, homodimers, homotrimers, and homotetramers, plotted as a function of molecular densities of Halo7-GluA1 expressed in the HEK293-PM. Curves are to help the eye (curve fitting with quadratic functions).

(**b**) The distributions of homodimer durations of the Halo7-GluA1 (37°C) and Halo7-TM (22°C) in the HEK293-PM. Each histogram could be fitted well with a single exponential decay function, providing the dimer lifetime (the durations given in parentheses are the homodimer lifetimes after correction for the photobleaching lifetime of the fluorescent probe).

Supplementary Figure 6

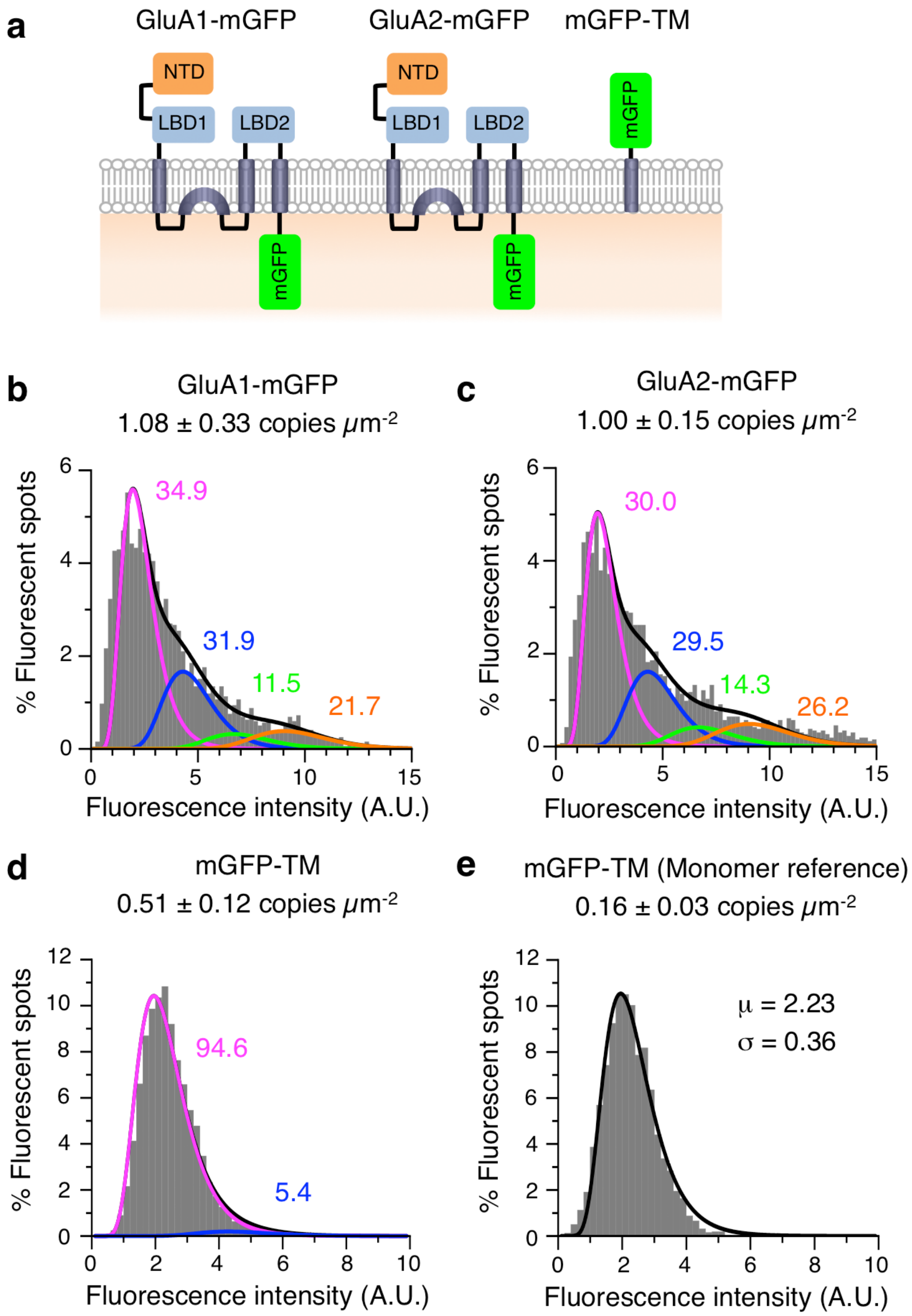

Supplementary Figure 6. GluA1 and GluA2 tagged with mGFP at their C-termini (rather than their N-termini as in the other experiments using ACP- and Halo7-tagged proteins) also exist as monomers, dimers, trimers, and tetramers in the HEK293-PM. Results obtained by using mGFP as a probe (GluA1-mGFP, GluA2-mGFP, and mGFP-TM), supplementing the data of ACP-GluA1 (**Fig. 1c**) and ACP-GluA2 (**Supplementary Fig. 3b**). For details of these figures, see the caption to **Fig. 1**.

(**a**) Schematic figure showing structures of GluA1-mGFP, GluA2-mGFP, and mGFP-TM.

(**b–e**) Distributions of the signal intensities of single individual fluorescent spots of the molecules shown in (**a**). Their expression levels (number densities) were the following. GluA1-mGFP, 1.08 ± 0.33 copies μm^{-2} (3,188 spots in 4 images [cells])(**b**); GluA2-mGFP, 1.00 ± 0.15 copies μm^{-2} (2,638 spots in 4 images [cells])(**c**); mGFP-TM, 0.51 ± 0.12 copies μm^{-2} (4,117 spots in 33 images [cells])(**d**); and mGFP-TM, 0.16 ± 0.026 copies μm^{-2} (3,179 spots in 10 images [cells])(**e**).

The fluorescent fraction of mGFP is generally 70 ~ 80% in steady-state cells[3-5], which is lower than the fluorescently-labeled fractions of Halo7-tag and ACP-tag proteins (nearly 100%). However, the tetramer fractions of GluA1-mGFP and GluA2-mGFP were comparable to those found for Halo7- and ACP-tagged GluA1 and GluA2 expressed at about the same number density in the PM. Perhaps, the slight propensity of mGFP dimer formation[2,6] compensated for the lower fluorescent-labeling efficiencies of mGFP-tagged molecules.

Supplementary Figure 7

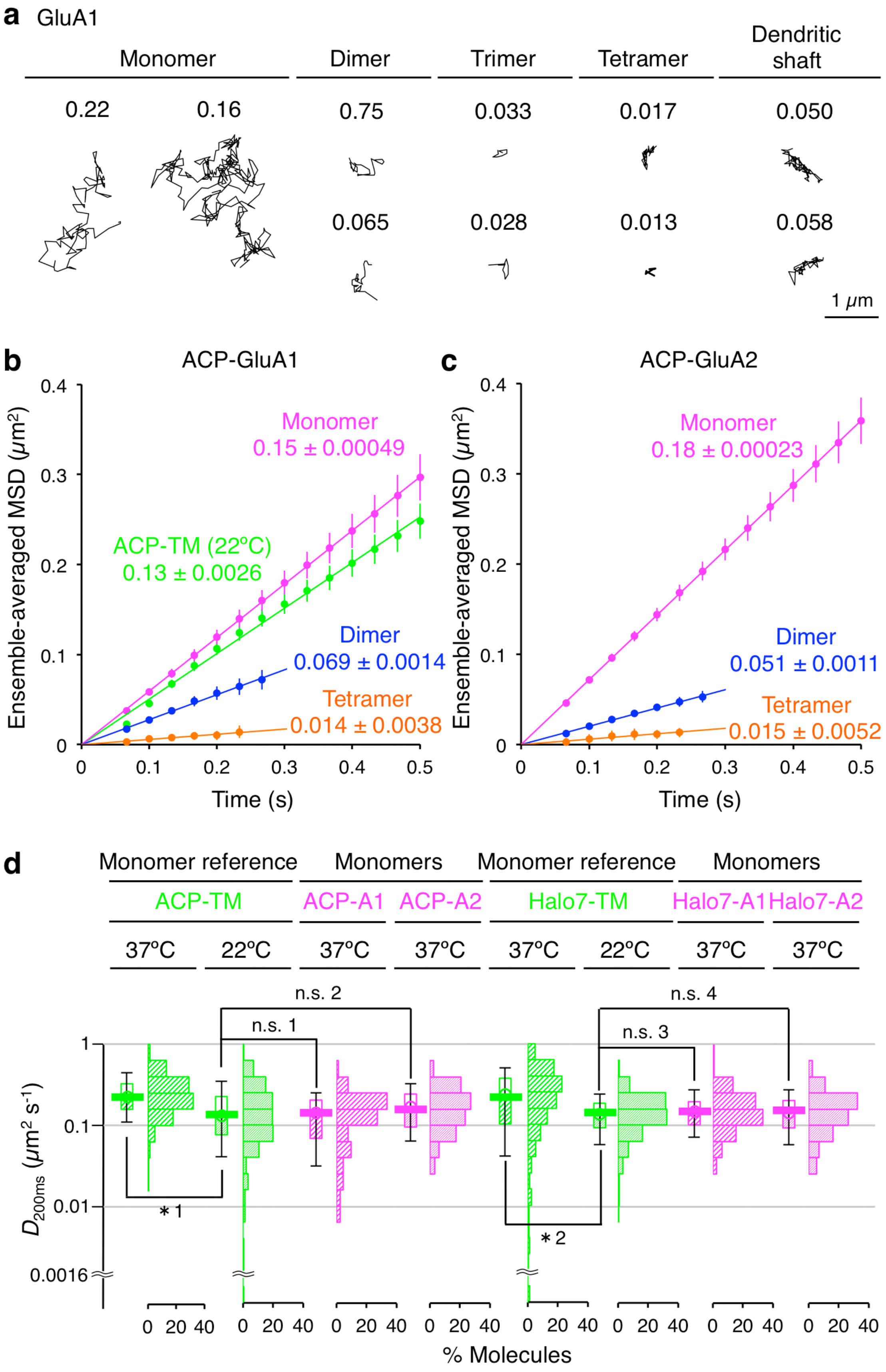

Supplementary Figure 7. Diffusion characteristics of GluA1, GluA2, ACP-TM, and

Halo7-TM in the HEK293-PM.

(**a**) Representative trajectories of GluA1 monomers, dimers, trimers, and tetramers in the HEK293-PM and Halo7-GluA1 (ATTO594-labeled) in the dendritic-shaft PM among 92, 104, 5, 10, and 1,348 trajectories, respectively (trajectories that exhibited the diffusion coefficients close to the median values were selected).

(**b, c**) The diffusion of a single molecule (a fluorescent spot) can be conveniently described by plotting the mean-square displacement (MSD) of the molecule against the time interval (x axis). When many single molecules are observed, the MSD for each time interval obtained for a single molecule can then be averaged over all of the observed single molecules, which is termed the "ensemble-averaged MSD" (y axis here). When the molecules undergo simple-Brownian diffusion, the ensemble-averaged MSD-Δt plot follows a straight line (allowing linear fitting as shown in the figure), and the mean diffusion coefficient can be obtained by dividing the slope by 4 (in the case of 2-dimensional diffusion). Here, the ensemble-averaged MSD-Δt plots for monomers, dimers, and tetramers of ACP-GluA1 (**b**) and for those of ACP-GluA2 (**c**) in the HEK293-PM are shown. The ensemble-averaged MSD-Δt plot for ACP-TM (observed at 22°C) is included in (**b**). Error bars represent standard errors. These plots could be fitted well with straight lines, showing that the monomers, homodimers, and homotetramers of GluA1 and GluA2 effectively undergo simple-Brownian diffusion in the time scales shown here. The mean diffusion coefficients are shown in the figure. The numbers of molecules observed are summarized in **Supplementary Table 5** (for monomers) and **Supplementary Table 10** (for monomers, dimers, and tetramers).

(**d**) Based on the results described in **b** and **c**, in the present report, the diffusion coefficient for each fluorescent spot in the time scale of ~200 ms was estimated by fitting its MSD-Δt plot in the time scale between 167 and 233 ms, which was termed D_{200ms}. The figure **d** shows the distributions of D_{200ms} values of individual GluA1 and GluA2 monomers (both ACP-tagged and Halo7-tagged ones) estimated at 37°C. The distributions were very similar to those of ACP-TM and Halo7-TM evaluated at 22°C. The breaks at a D_{200ms} of 0.0016 $\mu m^2 s^{-1}$ indicate the threshold between mobile and (apparently) immobile molecules, which was determined from the SEMs of the diffusion coefficients of Halo7-GluA1 and Halo7-GluA2 (0.00154 and 0.00133 $\mu m^2 s^{-1}$ respectively) in the dendritic-shaft PM, because the diffusion coefficients as small as or smaller than their SEMs were considered insignificant. Bars, circles, boxes, and whiskers indicate the median values, mean values, interquartile range interquartile range (25–75%), and 10–90% range, respectively. Asterisks and n.s. indicate P < and > 0.05, respectively, using the Brunner-Munzel test. The actual p values are the following: n.s. 1, 0.68; n.s. 2, 0.051; n.s. 3, 0.13; n.s. 4, 0.26; ＊1, < 2.2 x 10^{-16}; ＊2, < 2.2 x 10^{-16}. The statistical parameters for these experiments are summarized in **Supplementary Table 5**.

Supplementary Figure 8

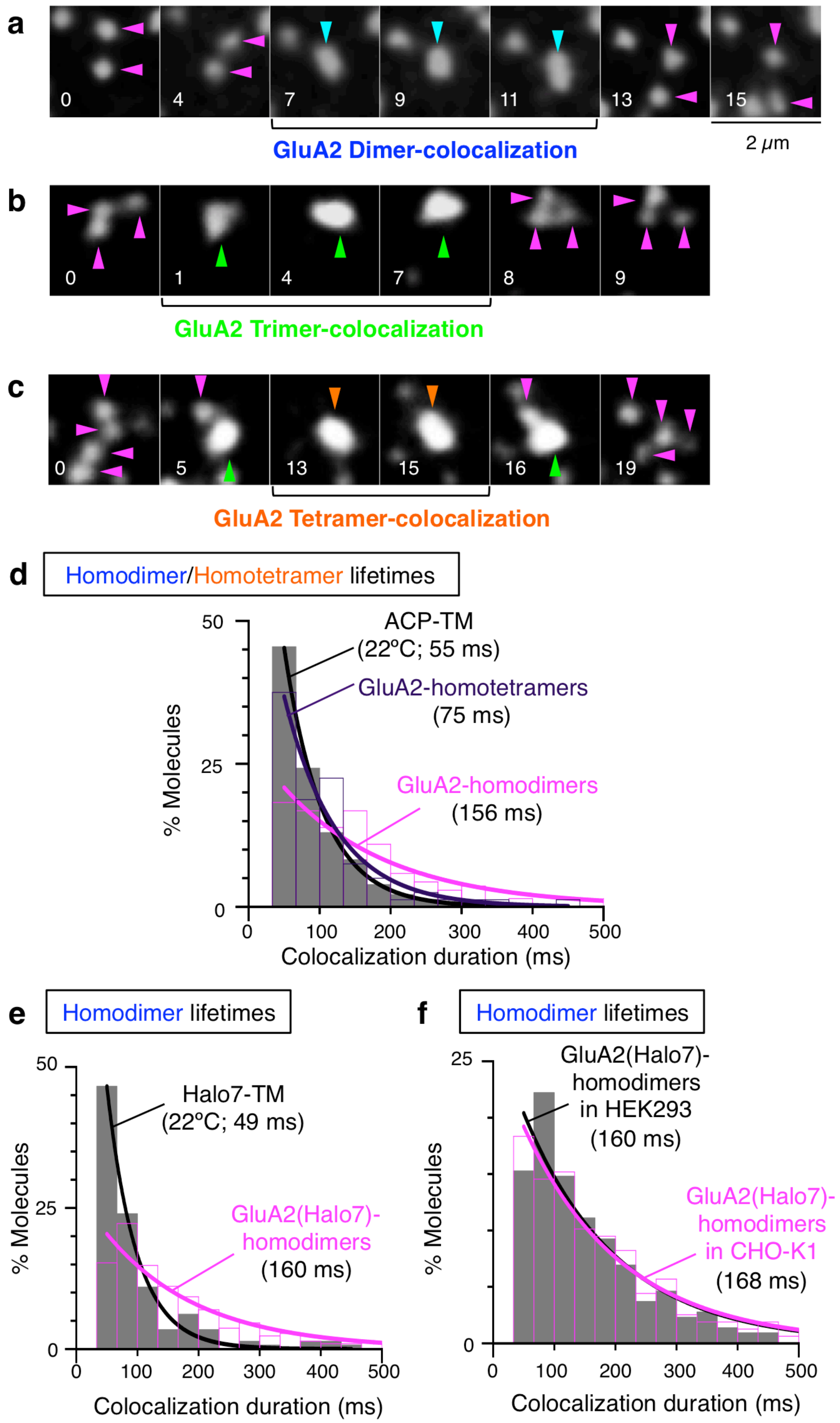

Supplementary Figure 8. GluA2 monomers dynamically form metastable homooligomers with exponential lifetimes of less than 200 ms, and dissociate into monomers, just like GluA1 homooligomers; single-color experimental results.

GluA2 results (using both ACP- and Halo7-GluA2), supplementing the ACP-GluA1 data (**Fig. 3**), as well as the Halo7-TM results, supplementing the ACP-TM data (**Fig. 3d**). For details of these figures, see the caption to **Fig. 3**. For statistical parameters, see **Supplementary Table 4**.

(**a–c**) Typical single-molecule fluorescence image sequences of two, three, and four diffusing ACP-GluA2 molecules undergoing transient colocalization and codiffusion (transient dimerization, trimerization, and tetramerization) in the HEK293-PM (typical results from 137, 64, and 80 independent movies for **a**, **b**, and **c**, respectively).

(**d**) The duration distributions of the ACP-GluA2 homodimers and homotetramers (37°C) and ACP-TM (22°C; control for the incidental overlaps of two molecules) in the HEK293-PM. Each histogram could be fitted well with a single exponential decay function, providing the dimer and tetramer lifetimes. The durations given in parentheses are the homodimer and homotetramer lifetimes after the correction for the photobleaching lifetime of the fluorescent probe. Note that unassociated monomer-reference molecules may track together by chance over short periods of time for short distances, but the probability of this occurring for multiple frames is small, and therefore the longer colocalization durations of GluA2 (or GluA1; **Fig. 3**) imply the presence of molecular interactions between two, three, and four molecules, rather than incidental encounters (although molecular interactions are initiated by incidental encounters).

(**e**) Same as (**d**), but for the homodimers of Halo7-GluA2 and Halo7-TM (22°C).

(**f**) The homodimer lifetime of GluA2 (Halo7-GluA2) did not depend on the host cell line. The homodimer lifetime distribution of Halo7-GluA2 in the PM of the HEK293 cells, which were used throughout this study (in addition to hippocampal neurons in a primary culture), was compared with that in the PM of the CHO-K1 cells.

Supplementary Figure 9

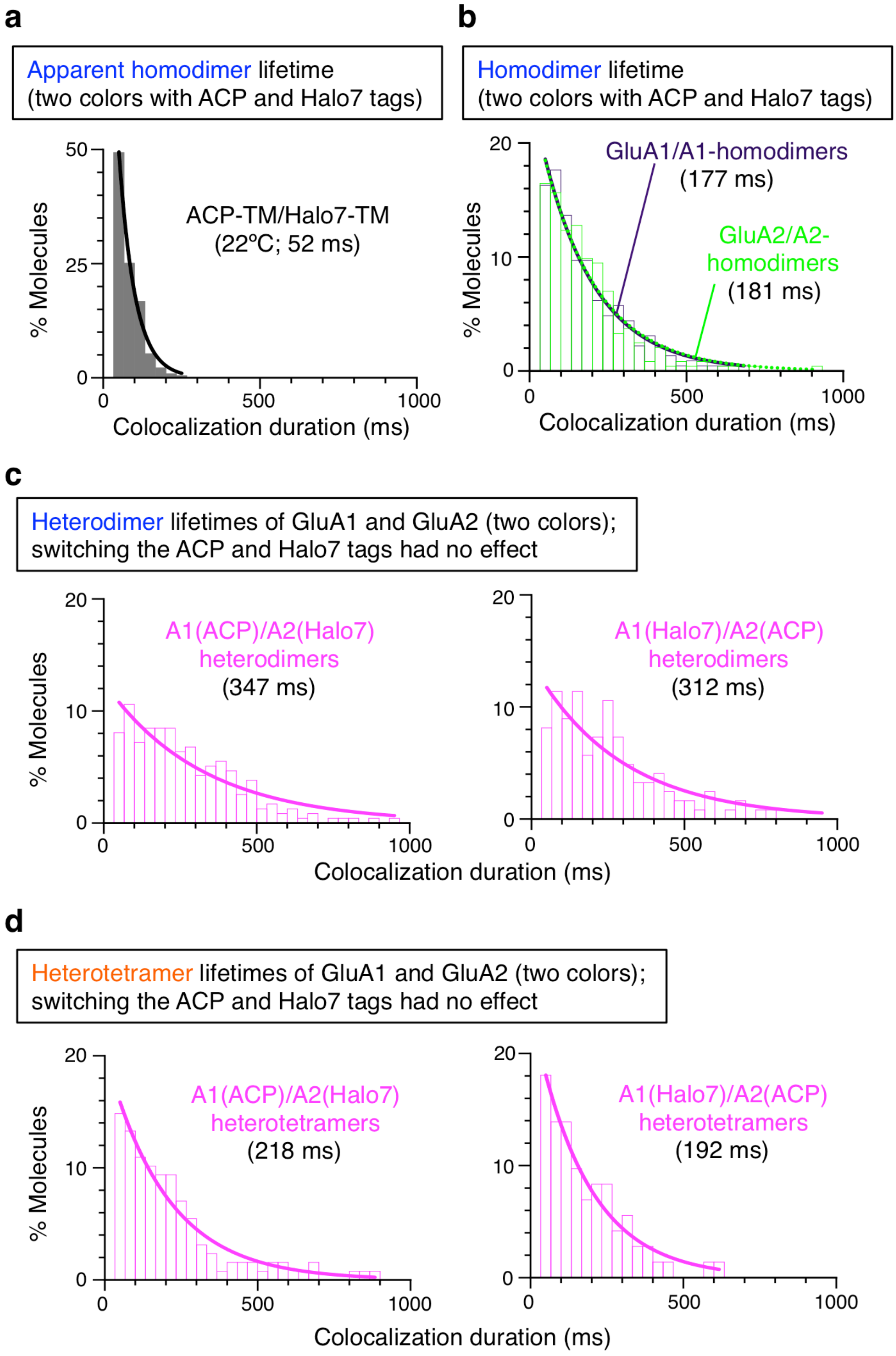

Supplementary Figure 9. Two-color single-molecule imaging provided homodimer lifetimes for GluA1 and GluA2 (as well as the incidental colocalization lifetimes of ACP-TM and Halo7-TM) similar to those obtained by single-color experiments,

supporting the subsecond lifetimes of GluA1 and GluA2 homodimers, and also provided the lifetimes of GluA1 and GluA2 hetero-dimers and hetero-tetramers.

Data supplementary to **Fig. 4d** and **e**. Each histogram could be fitted well with a single exponential decay function, providing the lifetimes of homodimers, heterodimers, and heterotetramers (shown in parentheses, after correction for the probe photobleaching lifetime).

(**a**) The duration distribution for incidental homodimers of TM at 22°C, using two different tag proteins, ACP and Halo7, for two-color experiments (thus, strictly speaking, this lifetime represents that for heterodimers of ACP-TM and Halo7-TM), providing an exponential lifetime of 52 ms. This is virtually the same as that of homodimers of ACP-TM molecules in the single-color results (55 ms) shown in **Fig. 3d**. In **Fig. 4d**, only the best-fit exponential function obtained here (for the pair of ACP-TM and Halo7-TM; two-color experimental result) is shown.

(**b**) The duration distribution (histogram) for homodimers of GluA1, using ACP-GluA1 and Halo7-GluA1 (purple), and that for homodimers of GluA2, using ACP-GluA2 and Halo7-GluA2 (green), obtained by two-color experiments. The homodimer lifetimes obtained by the two-color experiments (177 and 181 ms for GluA1 and GluA2 homodimers, respectively) shown here are virtually the same as those obtained by single-color experiments (164 ms for GluA1 homodimers [**Fig. 3d** and **Supplementary Fig. 5b**] and 156–160 ms for GluA2 homodimers [**Supplementary Fig. 8d, e**]). In **Fig. 4d**, only the best-fit exponential functions of the homodimer duration distributions obtained here (two-color experiments) are shown.

(**c, d**) The duration distributions (histograms) for heterodimers (**c**) and heterotetramers (**d**) of ACP-GluA1 and Halo7-GluA2 (left) and ACP-GluA2 and Halo7-GluA1 (right), obtained by two-color experiments. The duration histograms of heterodimers and heterotetramers (of GluA1 and GluA2; any combination of these for heterotetramers) shown in **Fig. 4d, e** were the averages of the histograms on the left and right (ACP- and Halo7-tags were switched between GluA1 and GluA2 in the left and right histograms) displayed in **c** and **d** here, respectively. For statistical parameters, see **Supplementary Table 6**.

Supplementary Figure 10

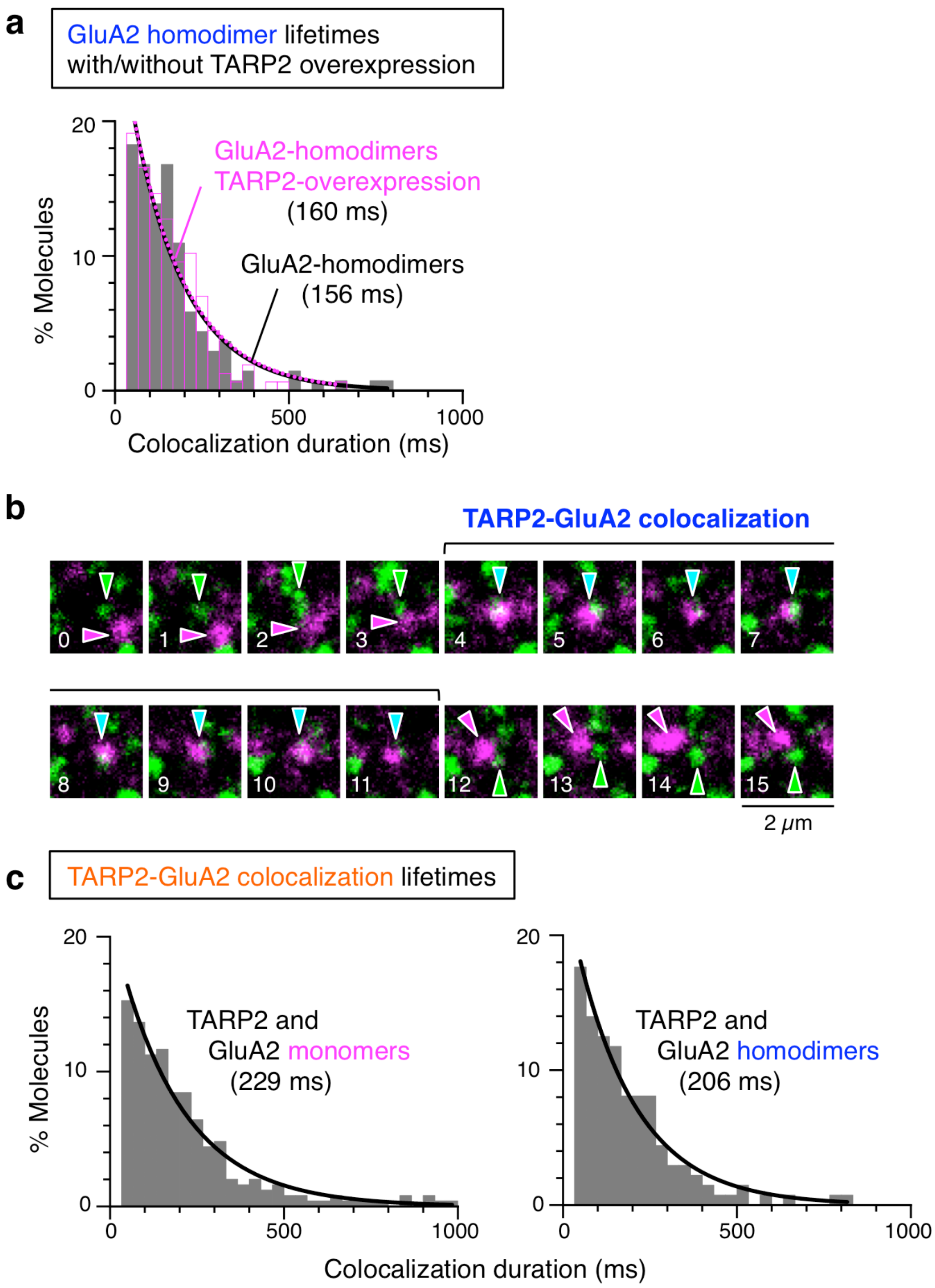

Supplementary Figure 10. TARP2 forms metastable complexes with GluA2.

Results obtained using GluA2, supplementing the GluA1 data shown in **Fig. 5**.

(**a**) The distributions of homodimer durations of ACP-GluA2 in the HEK293-PM with (magenta) and without (grey, see **Supplementary Fig. 8d**) TARP2-mGFP overexpression (expression levels 47 (±30) times higher than those of ACP-GluA2; n = 12 cells).

(**b**) Typical single fluorescent-molecule image sequences, showing that a TARP2-mGFP monomer (green arrowheads) formed a transient heteromer with an ACP-GluA2 monomer (magenta

arrowheads) in the HEK293-PM (a typical result from 385 independent movies). Cyan arrowheads indicate the heteromer.

(**c**) The duration distributions of the heteromers of TARP2-mGFP with ACP-GluA2 monomers (left) and dimers (right) in the HEK293-PM. The histograms could be fitted well with single exponential decay functions, providing the heteromer lifetimes, which are shown in parentheses (after correction for the probe photobleaching lifetimes). Note that homotetramers of GluA1 and GluA2 occurred rarely under our experimental conditions employed here, and their colocalizations with TARP2-mGFP took place quite seldomly, and thus we were unable to evaluate the colocalization lifetimes of TARP2-mGFP and homotetramers of GluA1 and GluA2. However, if the TARP2-mGFP binding had greatly stabilized the homotetramers of GluA1 and GluA2, as might have been expected from the single-particle cryo-electron microscopy (cryo-EM) data for the structure of the complex of TARP2 and GluA2 tetramers[7,8], then we should have been able to readily find the complexes of TARP2-mGFP and GluA1 or GluA2 homotetramers. Therefore, we concluded that the binding of TARP2 to GluA1 and GluA2 homotetramers would also be quite transient, and that it did not substantially stabilize the homotetramers.

For statistical parameters, see **Supplementary Table 7**.

Supplementary Figure 11

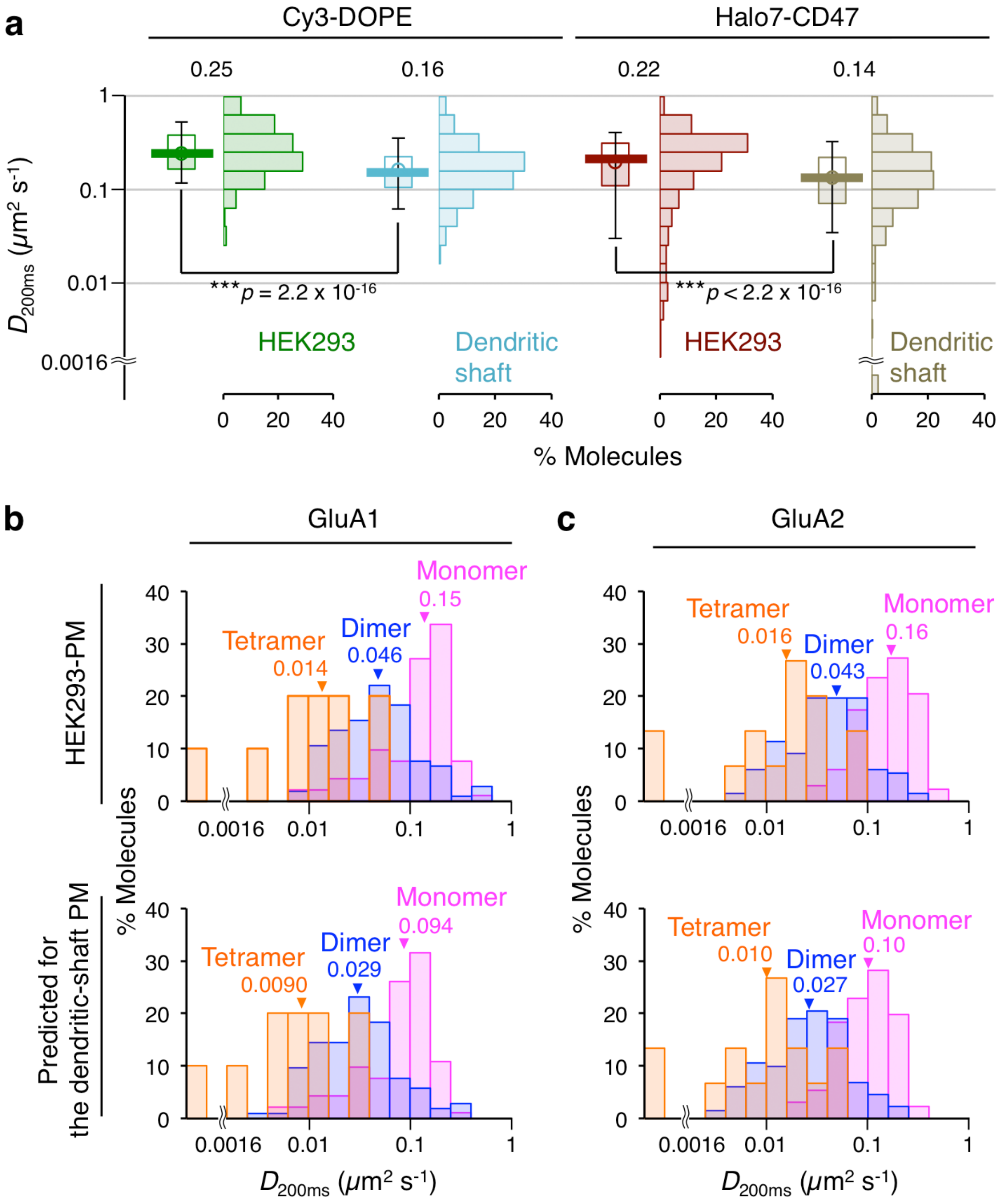

Supplementary Figure 11. The distributions of the diffusion coefficients (*D_{200ms}*) of monomers, homodimers, and homotetramers of GluA1 and GluA2 in the HEK293-PM and their predicted distributions in the dendritic-shaft PM, based on the comparison of the diffusion coefficients of fluorescently-labeled representative membrane molecules in the HEK293-PM with those in the dendritic-shaft PM.

The basic data for the GluA1 results shown in **Fig. 7c** and for the GluA2 data shown in **Supplementary Fig. 12c**. For the statistical parameters, see **Supplementary Tables 9** and **10**.

(**a**) The distributions of D_{200ms} of two fluorescently-labeled representative membrane molecules, Cy3-DOPE (non-raft phospholipid) and Halo7-CD47 (five-pass transmembrane protein), in the

HEK293-PM and dendritic-shaft PM. Bars, circles, boxes, and whiskers indicate the median values, mean values, interquartile ranges (25–75%), and 10–90% ranges, respectively. The median D_{200ms} values (shown on the top of each graph) for Cy3-DOPE and Halo7-CD47 in the dendritic-shaft PM are a factor of 1.56 smaller than those in the HEK293-PM. *** indicates p <0.001 using the Brunner-Munzel test (see the figure for the actual p values). For the break at 0.0016 $\mu m^2 s^{-1}$, see the caption to **Supplementary Fig. 7d**.
(**b, c**) The distributions of D_{200ms} of monomers, dimers, and tetramers of ACP-GluA1 (**b**) and ACP-GluA2 (**c**) in the HEK293-PM (top) and their predicted D_{200ms} distributions in the dendritic-shaft PM (bottom) estimated from the values in the HEK293-PM by dividing the D_{200ms} value for each molecule by 1.56, as determined in **a**. Arrowheads indicate the median D_{200ms} values. The GluA1 and GluA2 histograms shown here (top in **b** and **c**) are the bases for the graphs shown in **Fig. 7c** (left; GluA1) and **Supplementary Fig. 12c** (left; GluA2), respectively. The bottom histograms in **b** and **c** are the raw data for the results shown in **Fig. 7c** (extreme right; GluA1) and **Supplementary Fig. 12c** (extreme right; GluA2), respectively. For the breaks at 0.0016 $\mu m^2 s^{-1}$, see the caption to **Supplementary Fig. 7d**.

Supplementary Figure 12

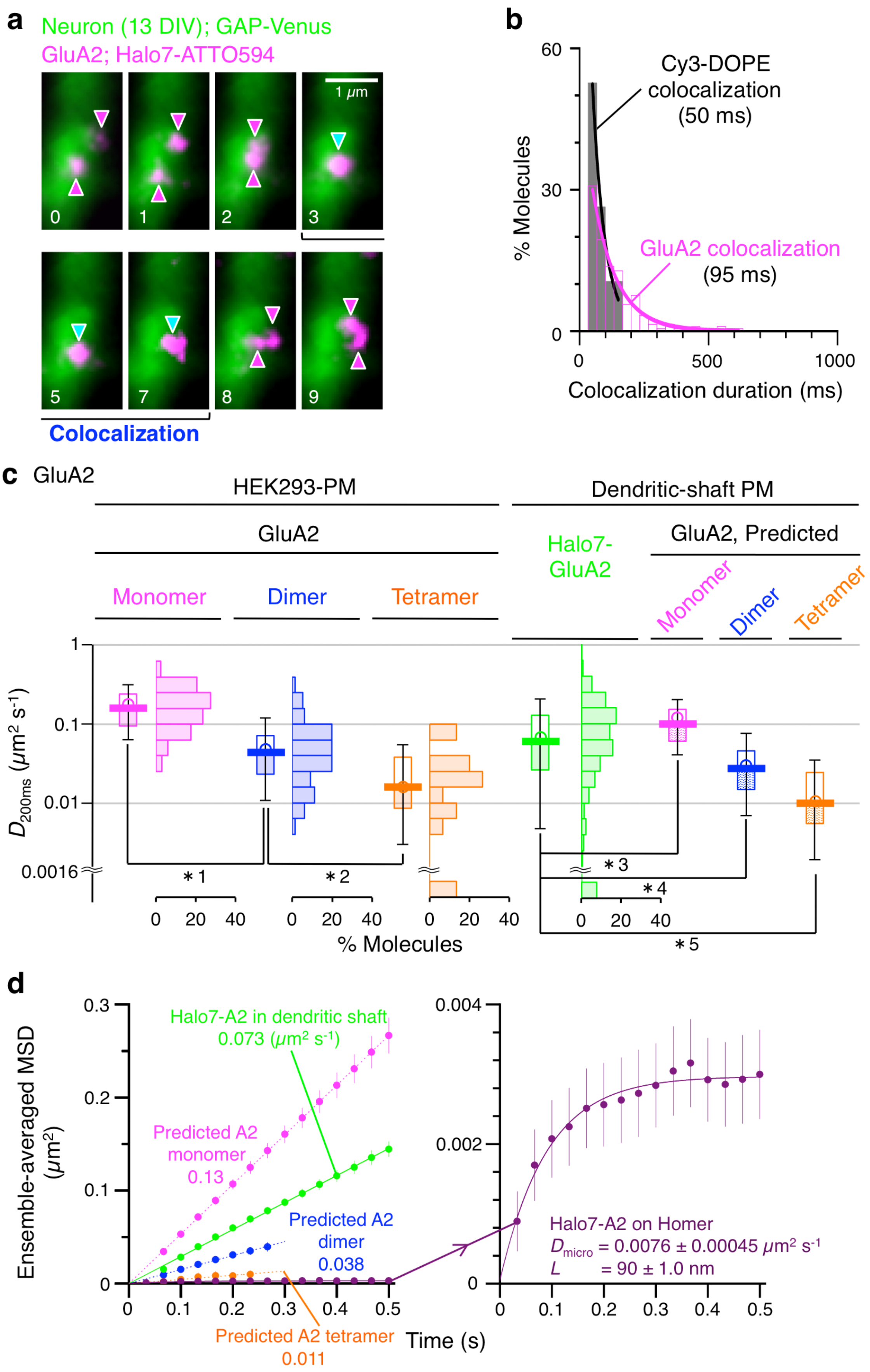

Supplementary Figure 12. Many GluA2 molecules existed as monomers and underwent intermittent transient dimerization/oligomerization in the PMs of neuronal dendritic shafts.

Results obtained using GluA2, supplementing the GluA1 data shown in **Fig. 7**. For details of these figures, see the caption to **Fig. 7**.

(**a**) A representative image sequence of two diffusing Halo7-GluA2 fluorescent spots in the dendritic-shaft PM, exhibiting transient colocalization–codiffusion (representative results from 211 independent video clips).

(**b**) The distribution of the colocalization durations of Halo7-GluA2 (n = 211 events) and that of Cy3-DOPE (n = 19 events) in the dendritic-shaft PM, giving exponential lifetimes of 95 and 50 ms, respectively.

(**c**) The distributions of D_{200ms} of ACP-GluA2 monomers, homodimers, and homotetramers actually measured in the HEK293-PM (left) and those of presumed ACP-GluA2 monomers, homodimers, and homotetramers in the dendritic-shaft PM (right) predicted from the HEK293-PM results (see **Supplementary Fig. 11c** for the raw data), as well as those of Halo7-GluA2 (labeled with ATTO594) in the dendritic-shaft PM (middle). Asterisks indicate $p < 0.05$ using the Brunner-Munzel test (the actual p values are the following: ＊1, $< 2.2 \times 10^{-16}$; ＊2, 6.3×10^{-3}; ＊3, 8.0×10^{-10}; ＊4, $< 2.2 \times 10^{-16}$; ＊5, 7.2×10^{-9}).

(**d**) The ensemble-averaged MSD plotted against time Δt (for its definition, see the caption to **Supplementary Fig. 7b, c**). The statistical parameters are summarized in **Supplementary Table 10**. Halo7-GluA2 underwent simple-Brownian diffusion in the dendritic-shaft PM at a mean diffusion rate between those of hypothetical ACP-GluA2 monomers and dimers (as shown by the linear fitting of the MSD-Δt plots in the figure on the left), whereas it exhibited confined diffusion within a confinement domain of 90 nm in the Homer1b-EGFP region (as shown by the confined-diffusion fitting in the figure on the right; when a molecule is confined within a domain, the MSD-Δt plot levels off at $L^2 3^{-1}$ in the 2-dimensional diffusion, where L is the confinement size [side length] in the model of the square confining lattice; D_{micro} represents the apparent diffusion coefficient within the confined domain)[9].

Supplementary Figure 13

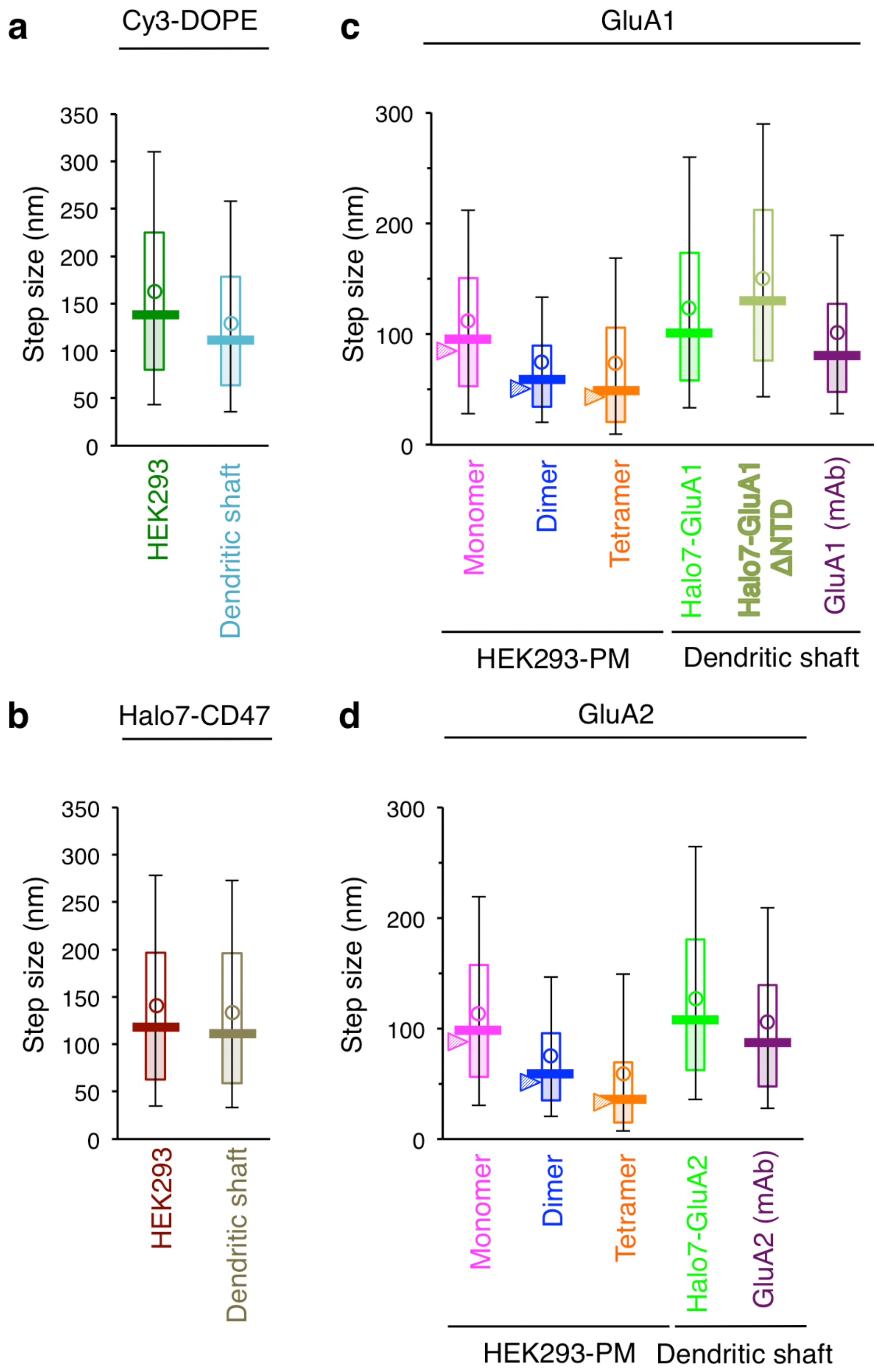

Supplementary Figure 13. The step sizes (the distances in which a single molecule moved between two consecutive frames in the image sequence) of Cy3-DOPE and Halo7-CD47 in the HEK293- and dentritic-shaft-PMs, as well as those of monomers, homodimers, and homotetramers of GluA1 and GluA2 (ACP-GluA1 and GluA2) expressed in the HEK293-PM and Halo7-GluA1, Halo7-GluA1ΔNTD, and Halo7-GluA2 labeled with ATTO594 and endogenous GluA1 and GluA2 labeled with ATTO594-conjugated respective mAbs in the dendritic-shaft PM, supporting the results

based on the diffusion coefficient (Fig. 7c and Supplementary Figs. 12c, 15c, and 16c).
(**a, b**) Step sizes of Cy3-DOPE (**a**) and Halo7-CD47 (**b**) in the PMs of HEK293 cells and neuronal dendritic shafts, showing that their diffusion is slower in the dendritic-shaft PM than in the HEK293-PM. The median step sizes for Cy3-DOPE and Halo7-CD47 in the dendritic-shaft PM are a factor of 1.15 smaller than those in the HEK293-PM. Note that the errors for single-molecule localizations are included in these step sizes, and therefore, the ratio of the median step size in the HEK293-PM vs. that in the dendritic shaft PM (a factor of 1.15) appears smaller than that expected from the ratio of D_{200ms}. Nevertheless, the larger step sizes of Cy3-DOPE and Halo7-CD47 in HEK293-PM were clear. The numbers of examined step sizes: Cy3-DOPE in the HEK293-PM, 7,997; Cy3-DOPE in the dendritic-shaft PM, 7,175; Halo7-CD47 in the HEK293-PM, 11,5379; Halo7-CD47 in the dendritic-shaft pM, 8,647.
(**c, d**) Step sizes of monomers, homodimers, and homotetramers of ACP-GluA1 (**c**) or ACP-GluA2 (**d**) in the HEK293-PM and step sizes of Halo7-GluA1 and Halo7-GluA1ΔNTD (**c**) and Halo7-GluA2 (**d**) in the dendritic-shaft PM (comprehensive histograms shown in **Supplementary Fig. 14**). Even the direct comparison of the step size of Halo7-GluA1 (GluA2) in the dendritic-shaft PM with that of the homodimers of GluA1 (GluA2) in the HEK293-PM clearly showed that Halo7-GluA1 (GluA2) diffuses faster than the homodimers of GluA1 (GluA2) expressed in the HEK293-PM. Furthermore, Halo7-GluA1ΔNTD showed significantly longer step sizes than Halo7-GluA1, in agreement with the results shown in **Fig. 7c**. The distributions of the step sizes of endogenous GluA1 and GluA2 were also obtained for comparison. Note that the homodimers of GluA1 (GluA2) in the dendritic-shaft PM would diffuse even slower: Triangles with slashes indicate the predicted median step sizes in the dendritic shaft-PM, estimated from the values in the HEK293-PM by dividing the median step sizes for each molecule by 1.15, as determined in **a** and **b**. Solid bars, circles, boxes, and whiskers indicate the median values, mean values, interquartile ranges (25–75%), and 10–90% ranges, respectively. The numbers of examined step sizes: ACP-GluA1 monomers, 13,926; ACP-GluA1 dimers, 2,007; ACP-GluA1 tetramers, 153; Halo7-GluA1 in dendritic-shaft PM, 49,913; Halo7-GluA1ΔNTD in dendritic-shaft PM, 15,610; endogenous GluA1 (mAb) in dendritic-shaft PM, 6,074; ACP-GluA2 monomers, 10,669; ACP-GluA2 dimers, 2,608; ACP-GluA2 tetramers, 186; Halo7-GluA2 in dendritic-shaft PM, 48,095; endogenous GluA2 (mAb) in dendritic-shaft PM, 6,525.

Supplementary Figure 14

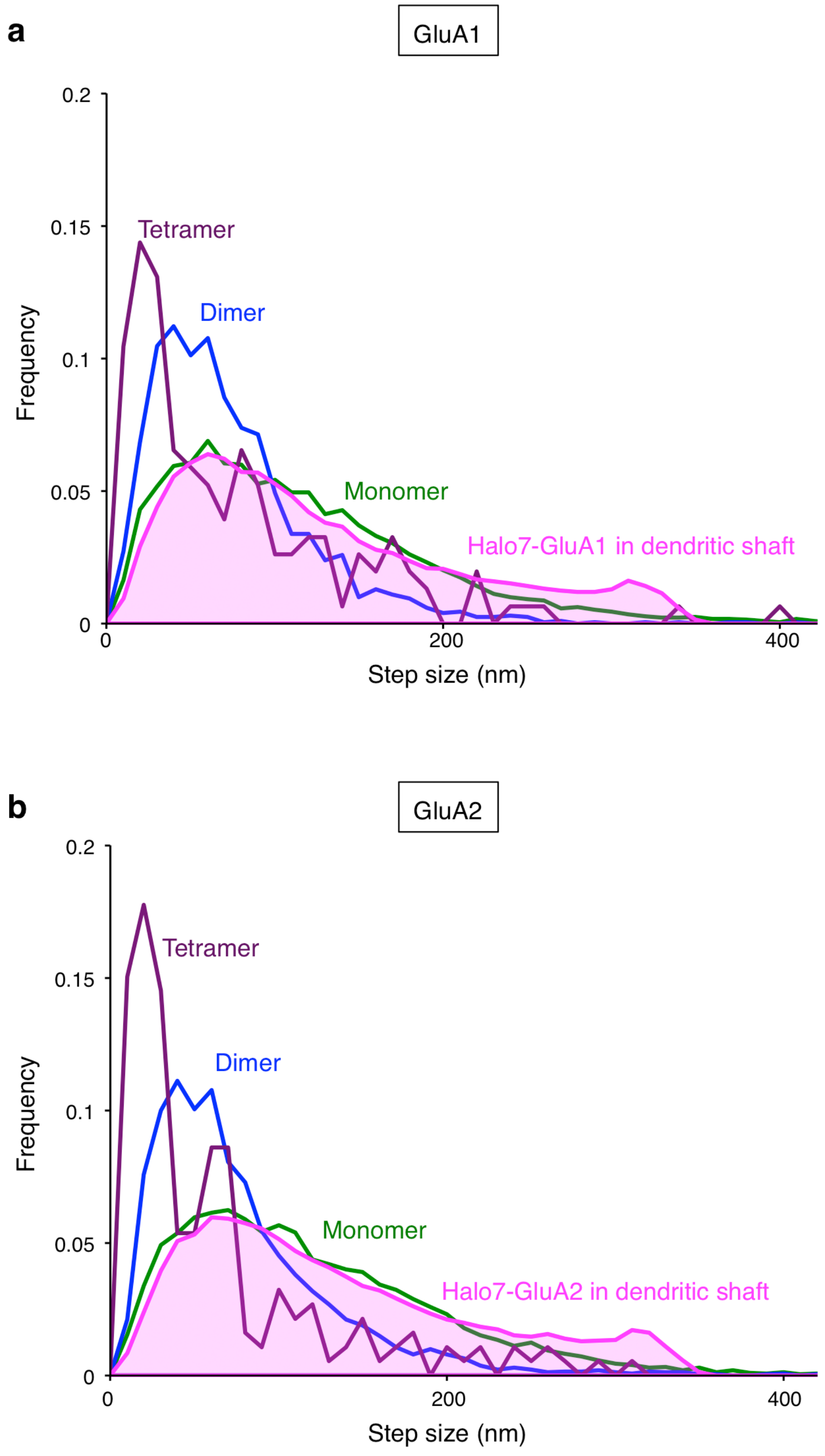

Supplementary Figure 14. The distribution of the step sizes of monomers (green), dimers (blue), and tetramers (purple) of GluA1 (ACP-GluA1) (a) and GluA2

(ACP-GluA2) (b) expressed in the HEK293-PM and Halo7-GluA1 (a) and Halo7-GluA2 (b) in the dendritic-shaft PM (magenta).

The raw data for the figures shown in **Supplementary Fig. 13c, d.**

Supplementary Figure 15

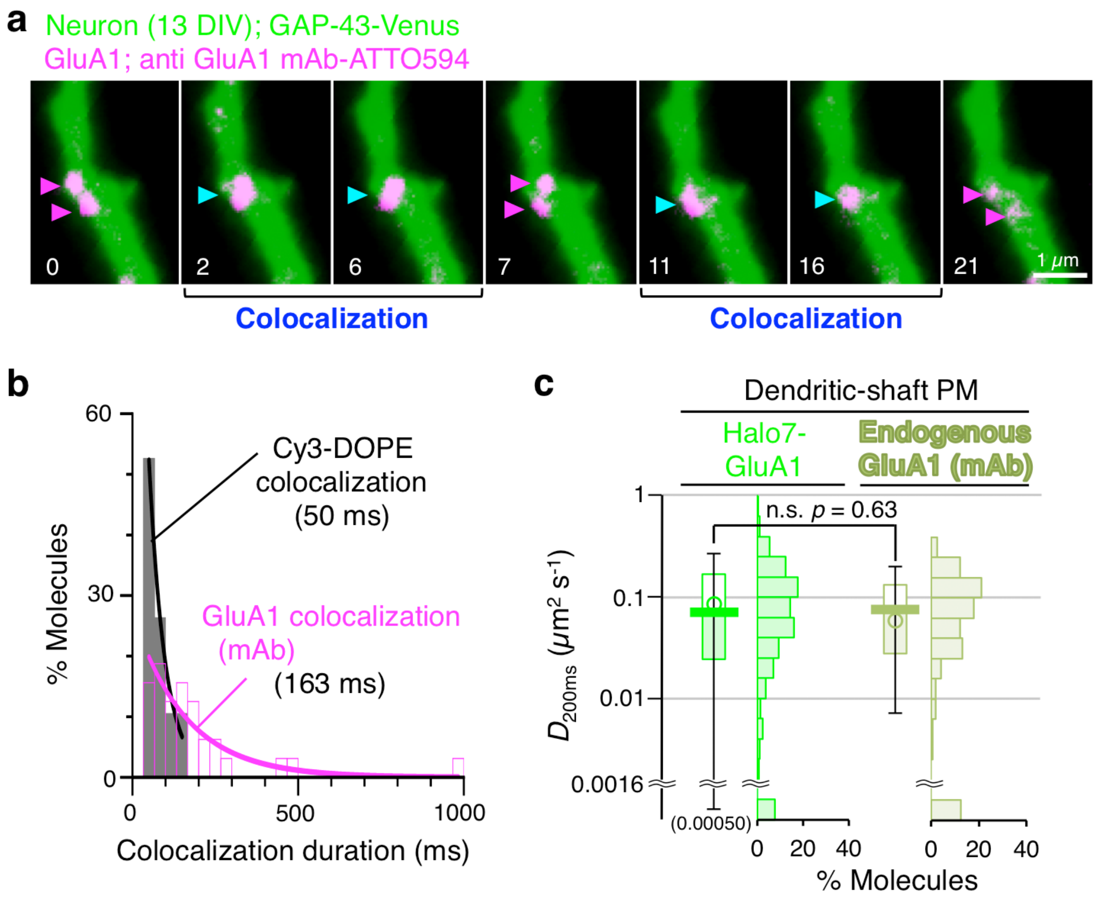

Supplementary Figure 15. Many endogenous GluA1 molecules (labeled with anti GluA1 mAb-ATTO594) existed as monomers and underwent intermittent transient dimerization/oligomerization in the PM of the neuronal dendritic shafts, supporting the data in Figure 7.

For details of the figures, see the caption to **Fig. 7c**.

(**a**) A representative image sequence of two diffusing endogenous GluA1 fluorescent spots (labeled with ATTO594-tagged anti GluA1 mAb; representative results from 32 independent video clips) in a dendritic shaft of a mouse hippocampal neuron in a primary culture (13 DIV). Two diffusing spots underwent intermittent transient colocalization–codiffusion (twice, each lasting for 167 and 200 ms).

(**b**) The distributions of the colocalization durations of endogenous GluA1 spots (n = 32) on the dendritic-shaft PM. As a control, Cy3-DOPE incorporated in the dendritic-shaft PM was investigated (n = 19), giving the exponential lifetimes of 163 and 50 ms, respectively. The colocalization lifetimes of antibody-bound endogenous GluA1 and GluA2 in the dendritic-shaft PM (163 and 129 ms, respectively; for GluA2, see **Supplementary Fig. 16b**) were longer than those obtained by using Halo7-GluA1 and Halo7-GluA2 expressed in the dendritic-shaft PM (104 and 95 ms, respectively; **Fig. 7b** and **Supplementary Fig. 12b**). The molecules undergoing colocalization–codiffusion might be

transiently crosslinked by the bound monoclonal antibodies, which may have prolonged the durations of colocalization–codiffusion.

(**c**) The D_{200ms} distribution of Halo7-GluA1 (the same histogram shown in **Fig. 7c**) and endogenous GluA1 (labeled with ATTO594-tagged anti GluA1 mAb) in the dendritic-shaft PM. No statistically significant differences were detectable.

Supplementary Figure 16

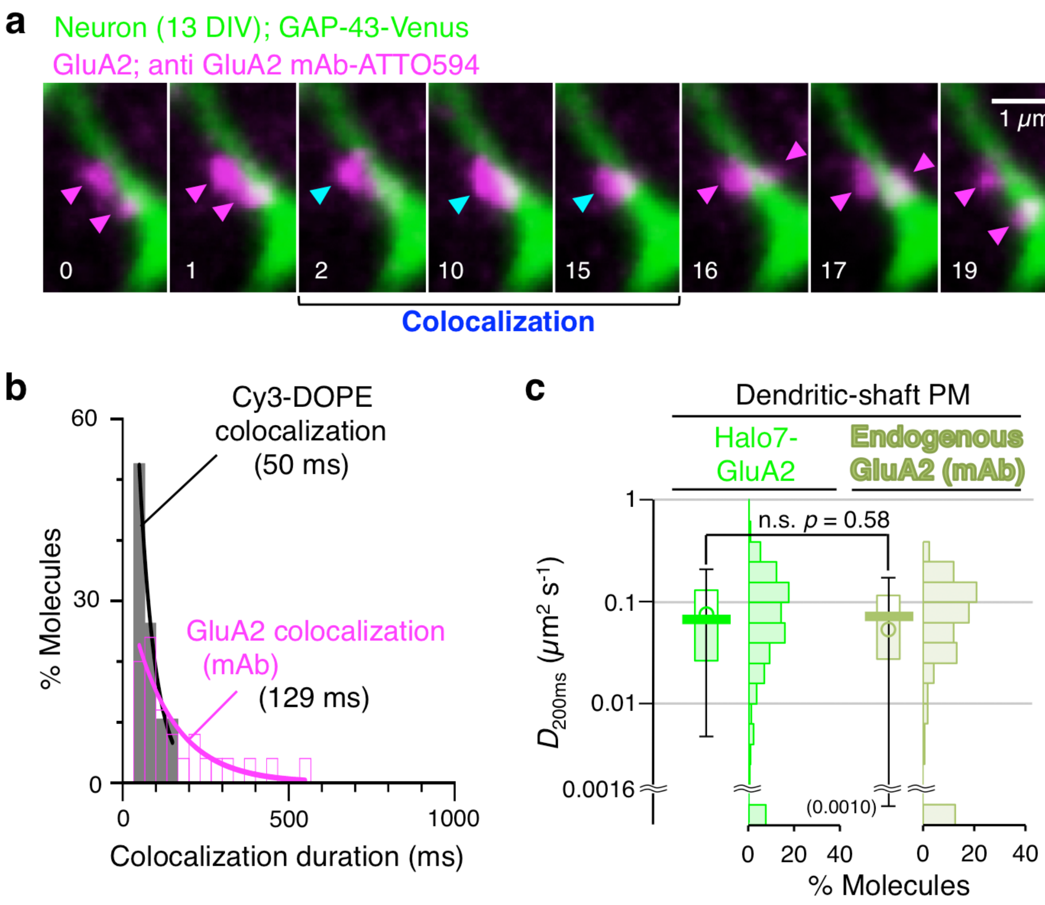

Supplementary Figure 16. Many endogenous GluA2 molecules (labeled with anti GluA2 mAb-ATTO594) existed as monomers and underwent intermittent transient dimerization/oligomerization in the PM of the neuronal dendritic shafts.

Results obtained using GluA2, supplementing the GluA1 data shown in **Supplementary Fig. 15**. For details of these figures, see the caption to **Fig. 7** and **Supplementary Fig. 15**.

(**a**) A representative image sequence of two diffusing endogenous GluA2 fluorescent spots (representative results from 25 independent video clips) in the dendritic-shaft PM.

(**b**) The distribution of the colocalization durations of endogenous GluA2 (n = 25) and that of Cy3-DOPE (n = 19) in the dendritic-shaft PM, giving exponential lifetimes of 129 and 50 ms, respectively.

(**c**) Same to **Supplementary Fig. 15c**, but for GluA2.

Supplementary Figure 17

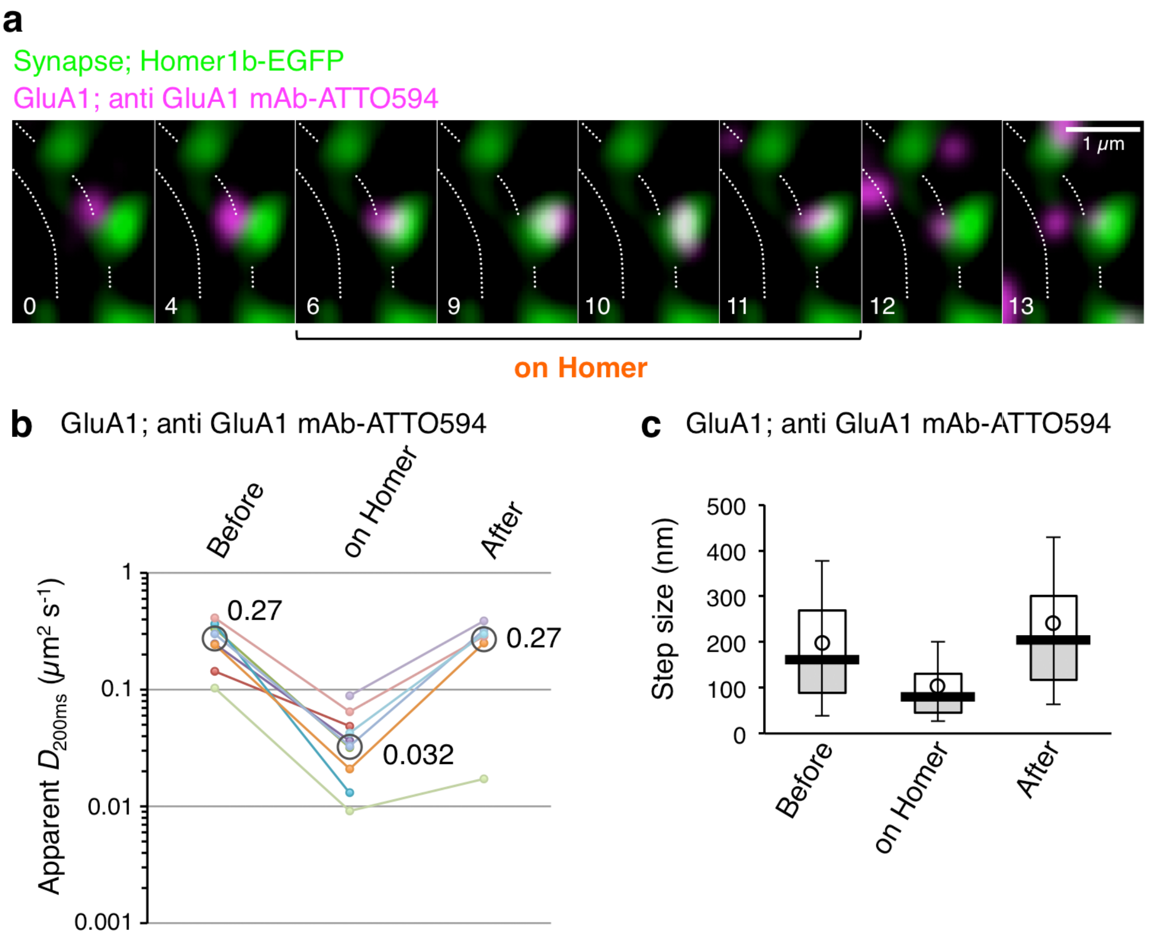

Supplementary Figure 17. Fast diffusing endogenous GluA1 monomers and dimers enter and exit from the synaptic regions.

Results obtained using anti GluA1 mAb-ATTO594, supplementing the GluA1 data shown in **Fig. 8**. For details of these figures, see the caption to **Fig. 8**.

(**a**) A typical image sequence of a fluorescent spot of endogenous GluA1 (conjugated by ATTO594-tagged anti GluA1 mAb; magenta spot; representative results from 8 independent video clips), entering a synaptic region from the dendritic-shaft PM and exiting from the synaptic region into the dendritic-shaft PM in mouse hippocampal neurons in a primary culture (13 DIV). Dotted lines indicate the peripheries of the dendritic shaft.

(**b**) The changes of D_{200ms} when each fluorescent GluA1 spot entered and/or exited from the synaptic region marked by Homer1b-EGFP.

(**c**) The changes of the step sizes of GluA1 spots when they entered and/or exited from the synaptic regions.

Supplementary Figure 18

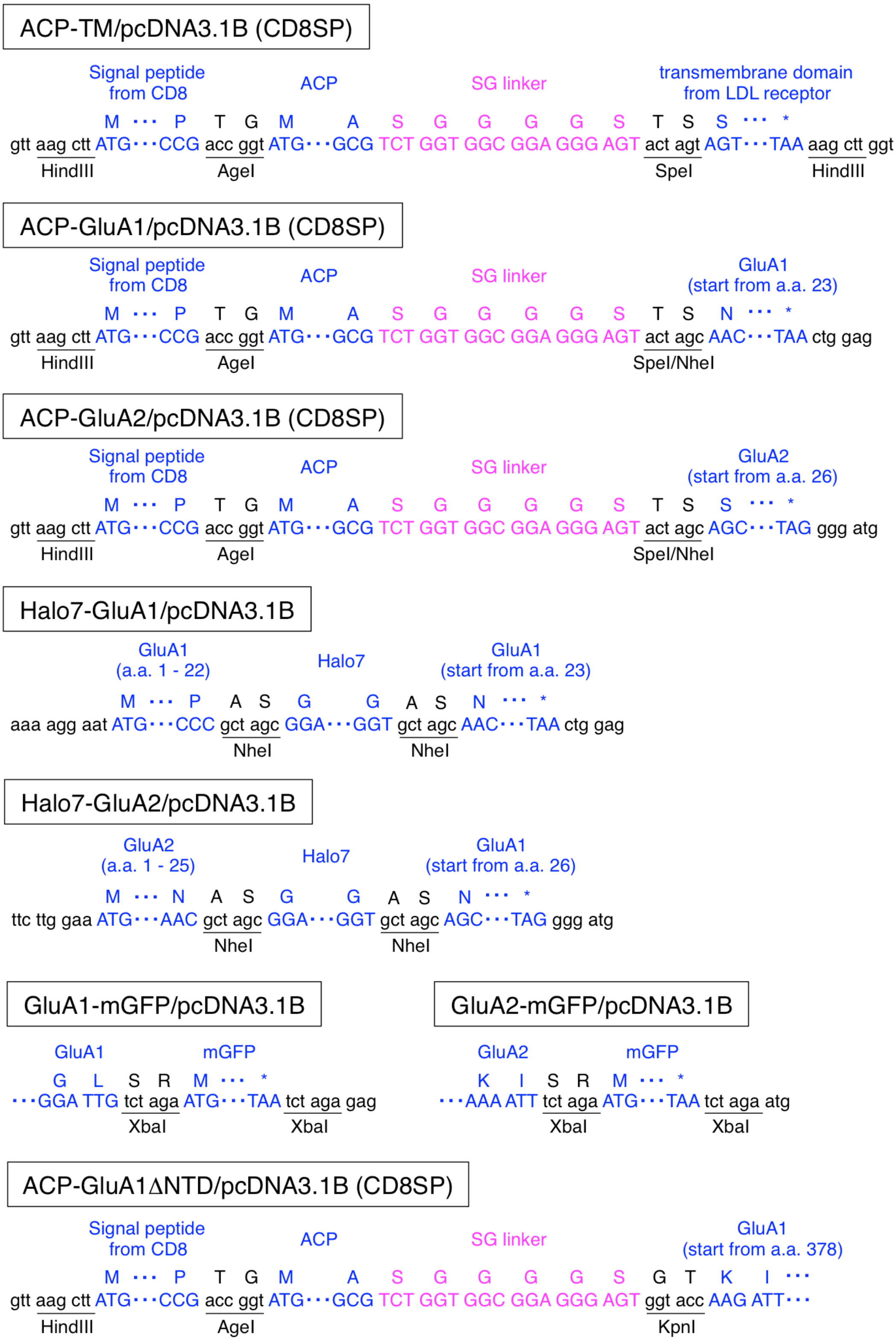

Supplementary Figure 18. Schematic structures of the cDNA constructs used for protein expressions in the HEK293 and CHO-K1 experiments, showing their multiple cloning sites, added signal sequences (for the cDNAs containing exogenous signal sequences), inserted tag proteins, linkers, and the target proteins.

Supplementary Figure 19

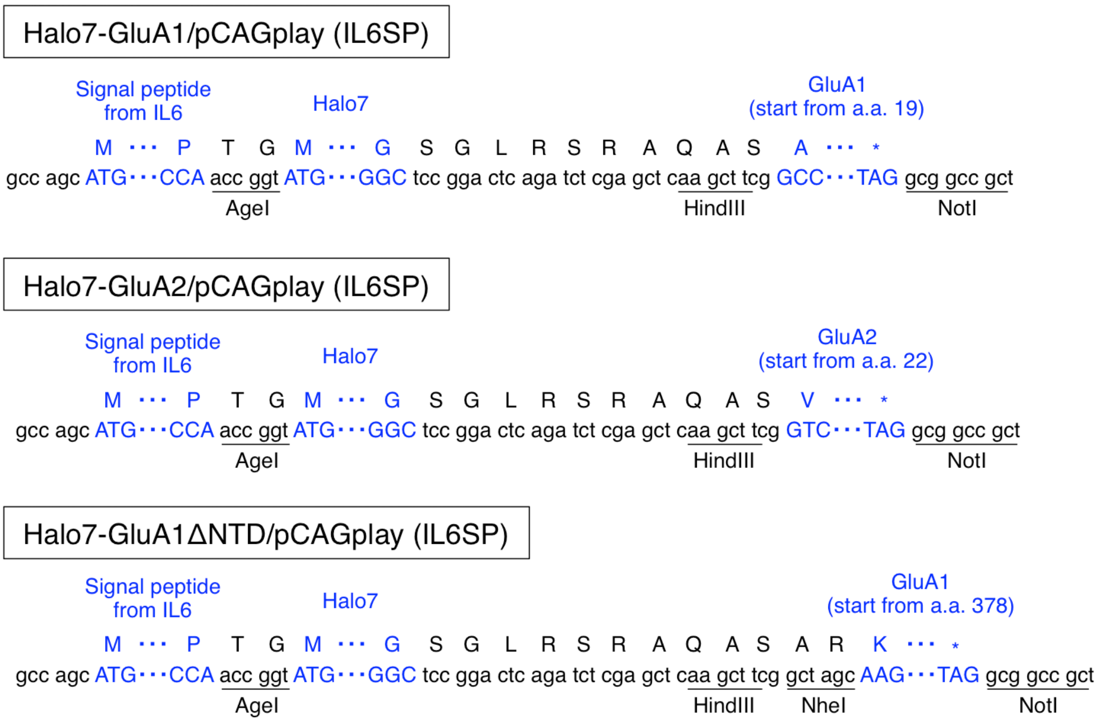

Supplementary Figure 19. Schematic structures of the cDNA constructs used for protein expression in the hippocampal neurons in culture, showing their multiple cloning sites, added signal sequences (for the cDNAs containing exogenous signal sequences), inserted tag proteins, linkers, and the target proteins.

Supplementary Figure 20

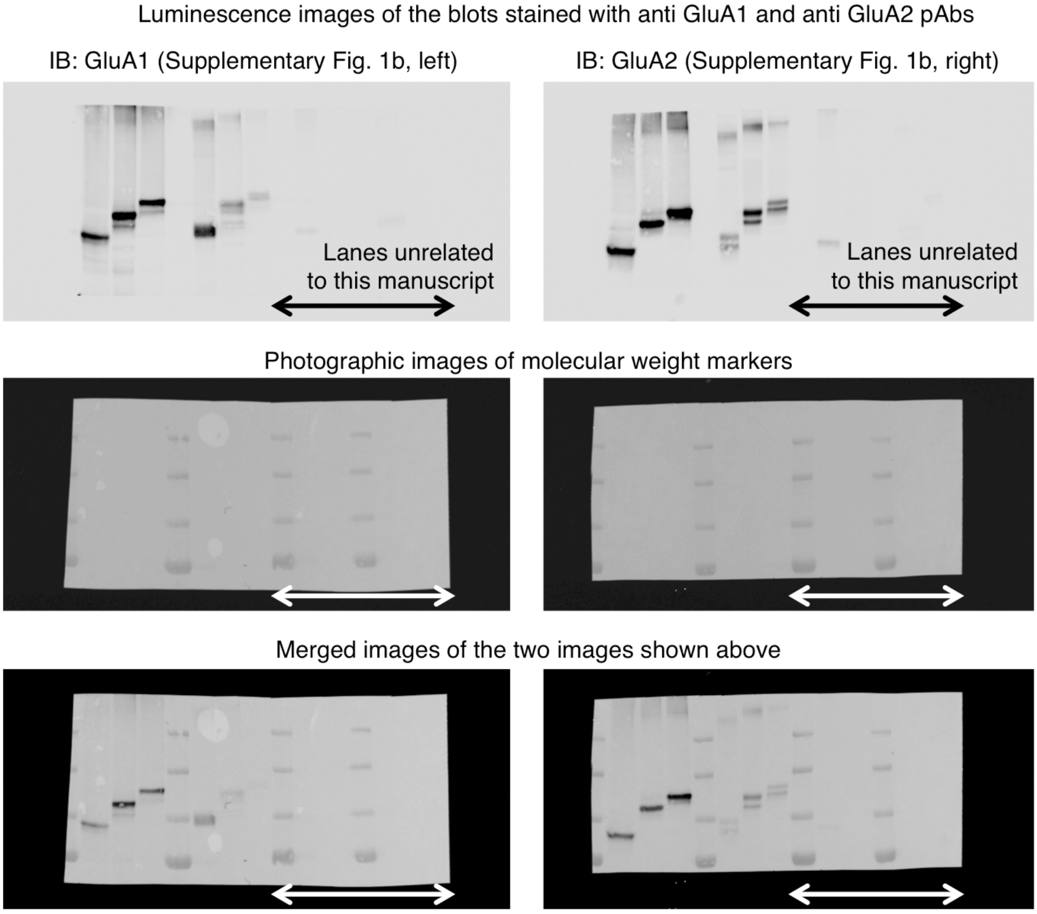

Supplementary Figure 20. The images of the entire western blot membranes.

Data supplementary to those shown in **Supplementary Fig. 1b**.

Supplementary Figure 21

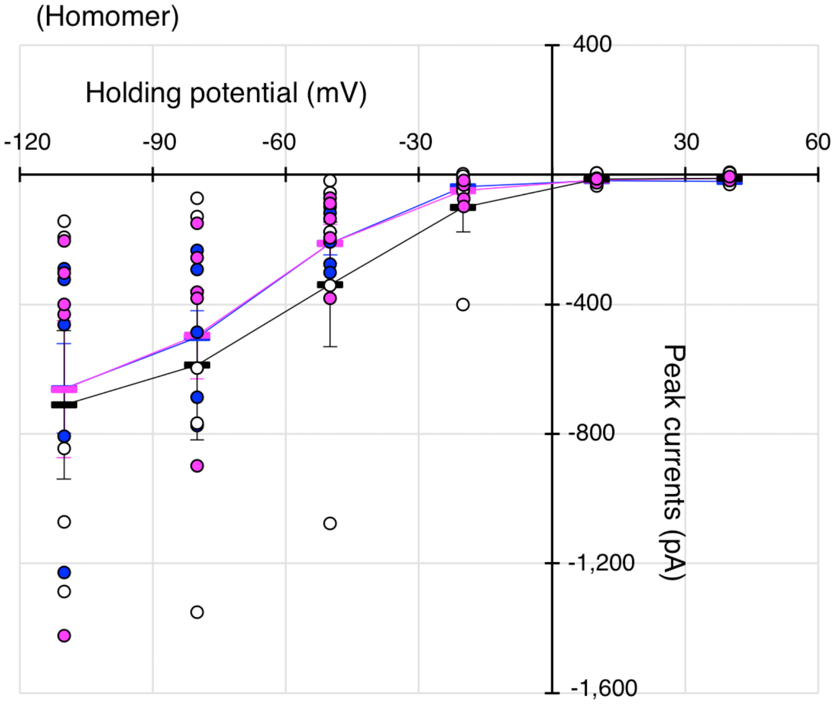

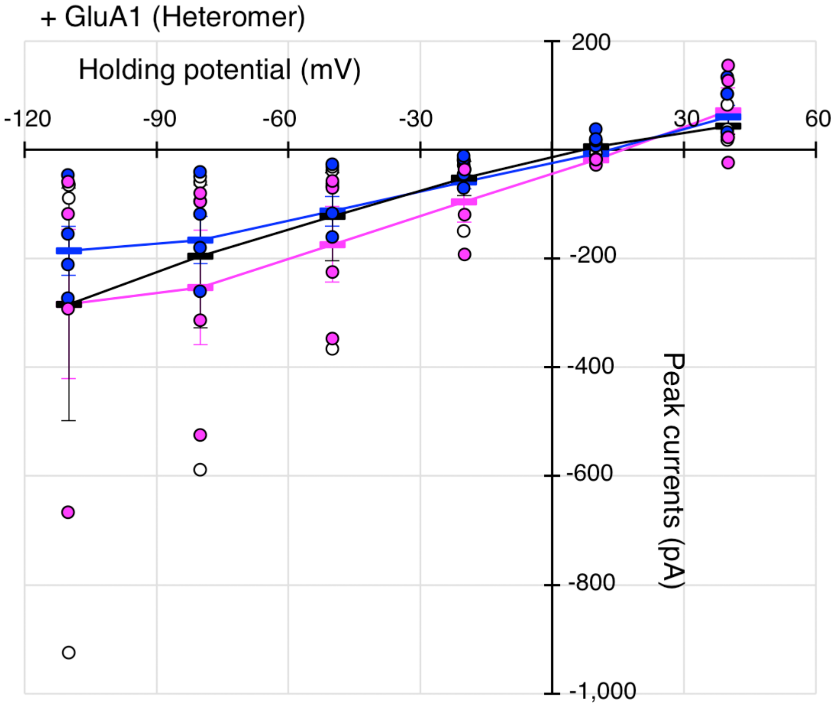

Supplementary Figure 21. All of the data points (dot plots) obtained for evaluating the current–voltage (I–V) relationships shown in Supplementary Fig. 2b.

To avoid excessive complications in **Supplementary Fig. 2b**, individual data points (all the data obtained) are shown here.

Supplementary References

1. Greger, I. H., Khatri, L. & Ziff, E. B. RNA editing at arg607 controls AMPA receptor exit from the endoplasmic reticulum. *Neuron* **34**, 759–772 (2002).

2. Suzuki, G. N. et al. Transient GPI-anchored protein homodimers are units for raft organization and function. *Nat. Chem. Biol.* **8**, 774–783 (2012).

3. Hastie, P. et al. AMPA receptor/TARP stoichiometry visualized by single-molecule subunit counting. *Proc. Natl. Acad. Sci. USA* **110**, 5163–5168 (2013).

4. Kasai, R. S. et al. Full characterization of GPCR monomer-dimer dynamic equilibrium by single molecule imaging. *J. Cell. Biol.* **192**, 463–480 (2011).

5. Sugiyama, Y., Kawabata, I., Sobue, K. & Okabe, S. Determination of absolute protein numbers in single synapses by a GFP-based calibration technique. *Nat. Methods* **2**, 677–684 (2005).

6. Zacharias, D. A., Violin, J. D., Newton, A. C. & Tsien, R. Y. Partitioning of lipid-modified monomeric GFPs into membrane microdomains of live cells. *Science* **296**, 913–916 (2002).

7. Twomey, E. C., Yelshanskaya, M. V., Grassucci, R. A., Frank, J. & Sobolevsky, A. I. Elucidation of AMPA receptor-stargazin complexes by cryo-electron microscopy. *Science* **353**, 83–86 (2016).

8. Zhao, Y., Chen, S., Yoshioka, C., Baconguis, I. & Gouaux, E. Architecture of fully occupied GluA2 AMPA receptor-TARP complex elucidated by cryo-EM. *Nature* **536**, 108–111 (2016).

9. Kusumi, A., Sako, Y. & Yamamoto, M. Confined lateral diffusion of membrane receptors as studied by single particle tracking (nanovid microscopy). Effects of calcium-induced differentiation in cultured epithelial cells. *Biophys. J.* **65**, 2021–2040 (1993).
