# Supplementary material for: AMPA receptors in the synapse turnover by monomer diffusion
Source: Nat Commun. 2019 Nov 20;10:5245. doi: 10.1038/s41467-019-13229-8 (PMC6868016; doi:10.1038/s41467-019-13229-8)
Supplement: Supplementary file 3 — Reporting Summary [file 41467_2019_13229_MOESM3_ESM.pdf]

nature research

Corresponding author(s): Kenichi G.N. Suzuki
Akihiro Kusumi
Shogo Oka

Last updated by author(s): Oct 17, 2019

Reporting Summary

Nature Research wishes to improve the reproducibility of the work that we publish. This form provides structure for consistency and transparency in reporting. For further information on Nature Research policies, see Authors & Referees and the Editorial Policy Checklist .

Statistics

For all statistical analyses, confirm that the following items are present in the figure legend, table legend, main text, or Methods section.

n/a	Confirmed	
☐	☒	The exact sample size (*n*) for each experimental group/condition, given as a discrete number and unit of measurement
☐	☒	A statement on whether measurements were taken from distinct samples or whether the same sample was measured repeatedly
☐	☒	The statistical test(s) used AND whether they are one- or two-sided<br>*Only common tests should be described solely by name; describe more complex techniques in the Methods section.*
☒	☐	A description of all covariates tested
☐	☒	A description of any assumptions or corrections, such as tests of normality and adjustment for multiple comparisons
☐	☒	A full description of the statistical parameters including central tendency (e.g. means) or other basic estimates (e.g. regression coefficient) AND variation (e.g. standard deviation) or associated estimates of uncertainty (e.g. confidence intervals)
☐	☒	For null hypothesis testing, the test statistic (e.g. *F*, *t*, *r*) with confidence intervals, effect sizes, degrees of freedom and *P* value noted<br>*Give P values as exact values whenever suitable.*
☒	☐	For Bayesian analysis, information on the choice of priors and Markov chain Monte Carlo settings
☒	☐	For hierarchical and complex designs, identification of the appropriate level for tests and full reporting of outcomes
☐	☒	Estimates of effect sizes (e.g. Cohen's *d*, Pearson's *r*), indicating how they were calculated

Our web collection on statistics for biologists contains articles on many of the points above.

Software and code

Policy information about availability of computer code

Data collection	Immunoblotting:<br>Luminoimage Analyzer LAS-3000 (Fuji Film)<br>L Process V2.21 (Fuji Film)<br>ImageGauge V4.22 (Fuji Film)<br><br>Whole-cell current recording:<br>Axoclamp 1D amplifier (Molecular Devices)<br>Digidata 1440 A digitizer (Molecular Devices)<br>pCLAMP10 software (Molecular Devices)<br><br>TIRF microscopy:<br>Home-built single-molecule imaging station built on Olympus IX-70 (Olympus)<br>(Koyama et al., Biophys. J., 2005; Suzuki et al., J. Cell Biol. 2007).
Data analysis	Single fluorescent-molecule tracking:<br>WinTrack, WinATR, and WinSAT, produced in house (Koyama et al., Biophys. J., 2005; Suzuki et al., J. Cell Biol. 2007; Fujiwara et al., Mol. Biol. Cell. 2016)<br><br>Calcium influx:<br>ImageJ (NIH)<br><br>Statistical analysis<br>Origin Pro 2018 (OriginLab)

Statview 5.0. (SAS)

For manuscripts utilizing custom algorithms or software that are central to the research but not yet described in published literature, software must be made available to editors/reviewers. We strongly encourage code deposition in a community repository (e.g. GitHub). See the Nature Research guidelines for submitting code & software for further information.

Data

Policy information about availability of data

All manuscripts must include a data availability statement. This statement should provide the following information, where applicable:

- Accession codes, unique identifiers, or web links for publicly available datasets
- A list of figures that have associated raw data
- A description of any restrictions on data availability

All the data that support the findings presented in this study are available from the corresponding author upon request.

Field-specific reporting

Please select the one below that is the best fit for your research. If you are not sure, read the appropriate sections before making your selection.

☒ Life sciences ☐ Behavioural & social sciences ☐ Ecological, evolutionary & environmental sciences

For a reference copy of the document with all sections, see nature.com/documents/nr-reporting-summary-flat.pdf

Life sciences study design

All studies must disclose on these points even when the disclosure is negative.

Sample size	Sample sizes were similar as reported in previous publications (Suzuki et al., Nat. Chem. Biol. 2012; Nemoto et al., Cell. Biochem. Biophys. 2017; Tsunoyama et al., Nat. Chem. Biol. 2018)
Data exclusions	No data were excluded in this study.
Replication	All experimental findings were reliably reproduced.
Randomization	All sample allocations were random.
Blinding	The sample preparation and observation were mostly preformed by the same operator.

Reporting for specific materials, systems and methods

We require information from authors about some types of materials, experimental systems and methods used in many studies. Here, indicate whether each material, system or method listed is relevant to your study. If you are not sure if a list item applies to your research, read the appropriate section before selecting a response.

Materials & experimental systems

n/a	Involved in the study	
☐	☒	Antibodies
☐	☒	Eukaryotic cell lines
☒	☐	Palaeontology
☐	☒	Animals and other organisms
☒	☐	Human research participants
☒	☐	Clinical data

Methods

n/a	Involved in the study	
☒	☐	ChIP-seq
☒	☐	Flow cytometry
☒	☐	MRI-based neuroimaging

Antibodies

Antibodies used

Antibodies used for single fluorescent imaging;
mouse anti-GluA1 monoclonal antibody (a gift from Prof. Richard L. Huganir, The Johns Hopkins University)
mouse anti-GluA2 monoclonal antibody (Millipore, MAB397)

Antibodies used for immunobloting, primary antibodies;
rabbit anti-GluA1 polyclonal antibodies (Enzo Life Sciences, ADI-905-416-1, 1:200)
rabbit anti-GluA2 polyclonal antibodies (Enzo Life Sciences, ADI-905-414-1, 1:200)

Antibodies used for immunobloting, seondary antibodies;

	HRP-conjugated goat anti-rabbit IgG (Invitrogen, 65-6120, 1:2000)
Validation	Validation details are available from the manufacture for each commercial antibody used in this study. Mouse anti-GluA1 monoclonal antibody provided by Richard L. Huganir laboratory is validated in the previous report (Diering et al., Neuron 2014)

Eukaryotic cell lines

Policy information about cell lines

Cell line source(s)	HEK293 cells and CHO-K1 cells were purchased from American Type Culture Collection and Japanese Collection of Research Bioresources, respectively. These are described in the Methods section.
Authentication	The identity of HEK293 cells was authenticated by PowerPlex16 STR (contract to Promega). CHO-K1 cells were not authenticized. These are described in the Methods section.
Mycoplasma contamination	HEK293 cells and CHO-K1 cells were proved to be mycoplasma free using MycoAlert (Lonza). These are described in the Method section.
Commonly misidentified lines (See ICLAC register)	n/a

Animals and other organisms

Policy information about studies involving animals; ARRIVE guidelines recommended for reporting animal research

Laboratory animals	We used C57BL/6J strain mice (Shimizu Laboratory Supplies, Kyoto, Japan). Day 16 embryos from pregnant mice were used for neuronal experiments.
Wild animals	n/a
Field-collected samples	n/a
Ethics oversight	All the animal experiments were conducted according to the Fundamental Guidelines for Proper Conduct of Animal Experiments and Related Activities in Academic Research Institutions under the jurisdiction of the Ministry of Education, Culture, Sports, Science and Technology of Japan and approved by the Committees on Animal Experimentation of Kyoto University.

Note that full information on the approval of the study protocol must also be provided in the manuscript.
