# Supplementary material for: AMPA receptors in the synapse turnover by monomer diffusion
Source: Nat Commun. 2019 Nov 20;10:5245. doi: 10.1038/s41467-019-13229-8 (PMC6868016; doi:10.1038/s41467-019-13229-8)
Supplement: Supplementary file 4 — Description of Additional Supplementary Files [file 41467_2019_13229_MOESM4_ESM.docx]

**Description of Additional Supplementary Files**

File name: Supplementary Movie 1.
Description: Typical behaviors of many single ACP(ATTO594)-GluA1 molecules expressed in the HEK293-PM at a number density of 0.50 copies µm^-2^ (see Figure 1c). GluA1 molecules largely exist as monomers at this number density, but sometimes form metastable homooligomers (yellow arrows). This movie shows a part of the HEK293-PM. All movies were recorded at video rate. Replay: 10-fold slowed from real time.

File name: Supplementary Movie 2.
Description: Typical transient merging and splitting of a Halo7-GluA1 spot (magenta, ATTO594 label) in the dendritic-shaft PM (see Figure 7a). The dendrite was visualized by expressing GAP-43-Venus (green). Replay: 30-fold slowed from real time.

File name: Supplementary Movie 3.
Description: Halo7-GluA1 spot (magenta, ATTO594 label) diffusing in and out of the synaptic region (see Figure 8a). Synaptic regions were visualized by expressing Homer1b-EGFP (green arrowheads). Replay: 15-fold slowed from real time.
