# Supplementary material for: AMPA receptors in the synapse turnover by monomer diffusion
Source: Nat Commun. 2019 Nov 20;10:5245. doi: 10.1038/s41467-019-13229-8 (PMC6868016; doi:10.1038/s41467-019-13229-8)
Supplement: Supplementary file 2 — Peer Review [file 41467_2019_13229_MOESM2_ESM.pdf]

Reviewers' comments:

Reviewer #1 (Remarks to the Author):

Synaptic plasticity is mediated by dynamic regulation of the quantity and composition of synaptic AMPA receptors (AMPAR). Previous studies also demonstrated that plasma membrane lateral diffusion of AMPAR from the dendritic shafts to extra-synaptic sites to the synapse (and vice versa) plays a crucial role in this process. In this interesting study, Morise et al propose a provocative hypothesis that lateral diffusion primarily involves AMPAR subunit monomers or dimers rather than fully formed tetramers as the currently believe. Not only that, they also propose that AMPAR tetramers are not stable entities and readily fall apart to dimers and monomers that could reform to tetramers at the synapse (even at different compositions) or recycle back. Indeed, such a mechanism may allow the rapid response and the flexibility required during the initial steps leading to the long term synaptic changes e.g. LTP and LTD. In a way: "pick and choose" as needed from the close by pool. Experimentally, the study is based on single-molecule imaging with total internal reflection fluorescence (TIRF) of AMPAR subunits GluA1 and GluA2 (the major form in the hippocampus) expressed in HEK293 cells and mouse hippocampal neurons. They show that the oligomeric state of both subunits is depended on the receptor expression level, such that the higher the expression, the higher the tetrameric fractions, with the N-terminal domain (NTD) playing a crucial role in facilitating the tetrameric formation (or stabilization). In dendritic spines of mouse hippocampal neurons, tetramers were present only transiently and monomers and dimers readily diffused in and out of the spine and only a limited number of these molecules became immobilized in the spine. Overall, in my view, the study is well conducted and the results are convincing; yet, in Neurons, it is not clear to me if the mAb labeling is saturating enough and truly captures the oligomric state; which is critical to the interpretation of the in vivo data. Also, it is not clear to me how the authors distinguish between simple co-localization and true assembly (argument in lines 195-198).

Regarding the conclusion put forward by the authors:
1. The assembly process of AMPAR is complicated and involves crossover of large globular domains between subunits in the dimer-of-dimer assembly. Also, intra- and inter-dimer interfaces involve large areas of strong connection. How this can be explained by rapid assembly, and particularly disassembly, of the tetramers near and in the synapse?
2. AMPAR are tightly associated with auxiliary proteins, particularly TARPs, which were shown to associate with the receptor already in the ER. TARPs were also shown to be particularly important for the lateral diffusion of AMPAR and for their encoring/immobilization at the synapse via phosphorylation/de-phosphorylation of the TARP C-tail. TARP tightly bind in between AMPAR subunits (i.e. requiring at least dimers if not tetramers for association). How this can be explained/fitted in the model?
3. The study would be much stronger if the authors could provide some data for LTP.

In summary, the study challenges a general believe in the field that AMPAR delivered to the plasma membrane are fully formed functional tetramers, and most probably also associated with auxiliary proteins, the TARPs at least. As such, the study is important enough to be published at a high profile journal, even if only for the sake of being challenged by future studies. If the authors are right, this would entirely change how we think about the mechanisms of induction and formation of synaptic plasticity.

Reviewer #2 (Remarks to the Author):

This manuscript from Morise and their colleagues present an elegant study about the molecular composition of AMPA receptors using single molecule imaging techniques. AMPARs are functional tetramers, and the structure has been believed stable. However, their results suggest that GluA1 and

GluA2 may be expressed as monomers or dimers in most of time on the plasma membrane, at least under some conditions. The mobility of monomers and dimers are much higher than tetramers.

They found that in HEK cells, fluorescent intensity of labeled AMPAR is consistent with 1- to 4-mers, with a significant fraction of them as monomer and dimer. The ratio depends on the surface concentration of the monomer, and was consistent with dynamic equilibration. The lifetime of the tetramers was measured to be surprisingly short: an order of 100 ms. Ca^{2+} transients caused by glutamate was proportional to their tetramer density, but supra-linear to the number density.

Data in neurons are done by antibody, and overall less convincing. They occasionally observed transient collocalization of AMPAR subunits, suggesting rapid subunit exchanges. The diffusion appears to be consistent with that of monomeric subunits in dendritic shafts.

Overall, the manuscript is well written, and many of their claims appear to be well supported by their data. The results have important implications in AMPAR turnover in and out of synapses. However, there are several technical concerns:

1. Diffusion measurements in neurons are performed with antibody, and it would be problematic. They argue that antibody would not cause any cross-linking based on their diffusion data. However, since it is known that antibody can crosslink AMPAR and change diffusion (like Penn, … Choquet, Nature, 2017), this argument would be not so convincing. Also, since the diffusion in dendrite is so different from HEK cells, it would be necessary to have an independent control. One possibility is to use overexpressed AMPAR subunits, just like in their HEK cell measurements.

2. They estimated the diffusion rate of monomers, dimers and tetramers in neurons from the diffusion of synthetic molecules, calibrated from HEK cell results. However, it would be important to measure the diffusion of monomer AMPAR subunit in neurons. This could be done by measuring overexpressed DeltaNTD in neurons (it may necessary to downregulate endogenous proteins with shRNA or CRISPR).

3. For their diffusion data in neurons, they should present the relationship between mean square distance and time, so that the readers can see that the diffusion is normal (they present MSD data for HEK cells in S-Fig. 8). This is especially important for diffusion in synapses, because the movement may show saturation as they may be trapped in small compartments. 200 ms may be too long for measurements of diffusion.

Minor
1. Figure 6: Homer would be not a marker of spines but synapses. This depends of course on the developmental stage etc, but their image does not show clear dendritic spine morphology. Perhaps they should call this “synapse” rather than “spine”.

Revisions made by our initiative (these revisions are not major, and thus are not indicated in the main text, Tables, and figures)

(1) To improve the readability of the paper and the presentations of the data, the data shown in **Supplementary Figs. 3b** and **4a** and **Supplementary Table 4** in the original manuscript are now shown as **Fig. 1** and **Table 1**, respectively, in the revised manuscript. As a result, all of the numbers of the figures are increased by 1 (Previous Fig. 1 is now Fig. 2, and so on).
The schematic figure, summarizing the results reported in this paper, was shown in **Fig. 6e** in the original manuscript, but it is now shown in **Fig. 9**.

(2) All of the displays and exponential fitting of the distributions (histograms) of the colocalization durations have been revised. In the original manuscript, the numbers of events (y-axis) were normalized by that of the first bin in the fitted curve (because the fitting curves and their differences were then easily visible). However, we found that the exponential fitting with the fixed value of 1 at the first bin induces small systematic errors. Therefore, in the revised manuscript, we now show the raw values of the %events (direct experimental data) in the histogram (without normalization), and the fitting was performed using two free parameters: the exponential decay time and the pre-exponential factor. The differences between the previous and present exponential decay times are less than 10% of the previous values, except for the homodimer lifetime of ACP-GluA1 (11%). The figures revised in this way are the following.

Figs. 3d, e and **4d, e**
Supplementary Figs. 5b, 8d–f, 9, 13b, and **14b**

(3) We have made two systematic mistakes in evaluating the diffusion coefficients reported in the original manuscript. The single-molecule localization errors were underestimated, and the slopes of the MSD-Δt plots were systematically underestimated by a factor of 1.25. These mistakes have been corrected in the revised manuscript. After this correction, the newly evaluated diffusion coefficients are greater than the values in the original manuscript by factors between 1.14 and 1.30, except for the diffusion coefficient of the ACP-A2 tetramer (decrease from 0.017 to 0.016 $\mu m^2/s$; i.e., by a factor of 0.94). However, these modifications did not affect any conclusions made previously in the original manuscript.

(4) In the process of considering the second point of Reviewer #2, we found that the method for evaluating the diffusion coefficients of the monomer reference molecules Cy3-DOPE and Halo7-CD47 in the dendritic-shaft PM should be refined. Combined with (3), their corrected diffusion coefficients are now greater by a factor of 1.41, as compared with the previous value described in the original manuscript. These changes did not affect any

conclusions made previously in the original manuscript. For details, please see the initial part of our response to the second point of Reviewer #2.

(5) In our displays of the distributions of the diffusion coefficients in the revised manuscript, all of the molecules exhibiting diffusion coefficients smaller than 0.0016 $\mu m^2/s$ are now placed in a single bin. The value 0.0016 $\mu m^2/s$ was the SEM of the diffusion coefficient of Halo7-GluA1 in the dendritic-shaft PM, and therefore, the diffusion coefficients smaller than this value were considered insignificant.

Reviewer comments:

Reviewer #1 (Remarks to the Author):

Synaptic plasticity is mediated by dynamic regulation of the quantity and composition of synaptic AMPA receptors (AMPAR). Previous studies also demonstrated that plasma membrane lateral diffusion of AMPAR from the dendritic shafts to extra-synaptic sites to the synapse (and vice versa) plays a crucial role in this process. In this interesting study, Morise et al propose a provocative hypothesis that lateral diffusion primarily involves AMPAR subunit monomers or dimers rather than fully formed tetramers as the currently believe. Not only that, they also propose that AMPAR tetramers are not stable entities and readily fall apart to dimers and monomers that could reform to tetramers at the synapse (even at different compositions) or recycle back. Indeed, such a mechanism may allow the rapid response and the flexibility required during the initial steps leading to the long term synaptic changes e.g. LTP and LTD. In a way: "pick and choose" as needed from the close by pool. Experimentally, the study is based on single-molecule imaging with total internal reflection fluorescence (TIRF) of AMPAR subunits GluA1 and GluA2 (the major form in the hippocampus) expressed in HEK293 cells and mouse hippocampal neurons. They show that the oligomeric state of both subunits is depended on the receptor expression level, such that the higher the expression, the higher the tetrameric fractions, with the N-terminal domain (NTD) playing a crucial role in facilitating the tetrameric formation (or stabilization). In dendritic spines of mouse hippocampal neurons, tetramers were present only transiently and monomers and dimers readily diffused in and out of the spine and only a limited number of these molecules became immobilized in the spine. Overall, in my view, the study is well conducted and the results are convincing; yet, in Neurons, it is not clear to me if the mAb labeling is saturating enough and truly captures the oligomric state; which is critical to the interpretation of the in vivo data. Also, it is not clear to me how the authors distinguish between simple co-localization and true assembly (argument in lines 195-198).

Thank you very much for your critical reading of our manuscript, and providing us with important criticisms for further improving it.

We would like to thank you for your kind comment, "Overall, in my view, the study is well conducted and the results are convincing" (the fifth line from the bottom of the second paragraph in your comment). Right after this comment, you raised two specific points that need clarifications (highlighted in green above). We addressed these two points in the revised manuscript in the following way.

- Whether the mAb labeling is saturating enough and truly captures the oligomric state.

The expression levels of endogenous GluA1 and GluA2 are far higher than the number of mAb molecules bound to the neuron surface. Using mAbs, we sampled very small portions of endogenous GluA1 and GluA2 molecules (probably fewer than only one out of 1,000 copies of endogenous molecules). In this way, we try to observe, rather than disturb, the behaviors of GluA1 and GluA2. This is a rather general method for the methods using probes.

For the revision of the manuscript, we further examined the behaviors of GluA1 and GluA2 fused with the Halo7-tag protein expressed in neurons, rather than using mAbs, just as we did in HEK cells. The results obtained by using Halo7-GluA1 and GluA2 are now shown in the main figures. For the comparison with the behaviors of endogenous GluA1 and GluA2 molecules, the data obtained with fluorescently-labeled mAbs are shown in the Supplementary Figures. Please refer to our response to the first point raised by Reviewer #2.

In fact, at the time of the original submission, we could not successfully express GluA1 and GluA2 conjugated with the tag proteins ACP or Halo7 in primary neurons, and thus used monoclonal antibodies. The comments by Reviewers #1 and #2, as well as by the Editor, strongly motivated us to try harder to express tag-protein-conjugated GluA1 and GluA2 in neurons, and as a result, we obtained data under better conditions. Therefore, we are extremely thankful for this important point raised by Reviewers #1 and #2 and the Editor.

- How the authors distinguish between simple co-localization and true assembly (argument in lines 195-198)

In our assay of the fractions of molecules that existed as monomers, dimers, ..., we did not make the distinction between incidental colocalizations and true molecular assemblies (therefore, when necessary, we used terms like “two, three, and four colocalized molecules” and “apparent tetramers”; **p. 7**), due to the following two reasons. First, since the monomer reference molecules ACP-TM and mGFP-TM exhibited no incidental colocalizations that appear like trimers and tetramers in the range of the number densities of molecules employed here (**Fig. 1b**; **Supplementary Figs. 3a** and **6d, e**), we did not need to make any corrections for the fractions of apparent trimers and tetramers. Second, although the non-distinction of true and apparent dimers in this method would cause a slight overestimation of true dimers and a slight underestimation of monomers, since the most important point we make in this report is the presence of considerable fractions of monomers, underestimating the monomer fraction is acceptable for this paper. Therefore, for the sake of simplicity, we employed the molecular fractions directly determined for

fluorescently-labeled GluA1 and GluA2 molecules. This is now explained in the revised manuscript at the end of the second paragraph on **p. 7**, and in **the new subsection called "Determining the fractions of monomers, dimers, trimers, and tetramers (two, three, and four colocalized molecules) of transmembrane proteins"** in the **Methods section** on **p. 30**.

In our colocalization lifetime measurements, we also did not distinguish simple co-localization and true assembly. This is mainly due to the limitations of the time resolution and the signal-to-noise ratios obtainable for the specimens with our instrument (although it is the state-of-the-art), as previously described in Suzuki et al. (Nat. Chem. Biol. 2012, ref#41) and Kasai et al. (Cell Biochem. Biophys. 2018, ref#69).

In principle, the histogram of colocalization durations of two molecules (including both apparent and true dimer cases) could be quite complex (for ease of explanation, let us explain by taking the case of dimers). Even in the case where no specific interactions between two molecules occur (incidental colocalization), the distribution of incidental colocalization durations (histogram) could be described by a complex function ($h_{\text{incidental}}$). Furthermore, in the case where a specific interaction of two molecules takes place (where true dimers are formed), the two molecules must first have close encounters within the diffraction-limited distance (240 nm, as described in the Results and Methods sections; this marks the beginning of the colocalization), undergo specific interactions (forming true dimers), and then after dissociation, become separated by more than the diffraction-limited distance of 240 nm (marking the end of the colocalization). Namely, the duration distribution ($h_{\text{composite}}$) would be the sum of the distributions of the following three durations: the duration from the encounter of two molecules within the distance of 240 nm until the binding of two molecules (h_{before}), the duration of the actual binding of two molecules ($h_{\text{true-dimers}}$), and the duration from the dissociation of two molecules until they become separated over the distance of 240 nm (h_{after}). Since we did not distinguish these two cases described by $h_{\text{composite}}$ and $h_{\text{incidental}}$ (where the actual molecular binding does and does not occur, respectively), the function describing the observed duration distribution would be the sum of these two complex functions with a proper weight c; i.e., $h_{\text{observed}} = [1\text{-}c]h_{\text{incidental}} + ch_{\text{composite}}$, where c is a real number satisfying $0 \le c \le 1$ (representing a measure for the fraction of molecules that became located within the 240-nm range and formed a dimer).

However, due to the insufficient time resolution (33 ms/frame) and limited signal-to-noise ratios in our single-molecule imaging movies, the histogram h_{observed} would not allow determinations of c, $h_{\text{incidental}}$, and $h_{\text{composite}}$, but appeared like a single exponential function, characterized by a single exponential decay constant between 164 and 335 ms (**Figs. 3d** and **4d**, **Supplementary Fig. 5b**). The lifetime obtained in this way (for h_{observed}) was

considered sufficiently long as compared with the lifetime obtained by approximating $h_{incidental}$ as a single exponential function (49 and 55 ms; **Figs. 3d** and **4d**, **Supplementary Fig. 5b**; again probably due to the lack of sufficient time resolutions and the limited signal-to-noise ratios of our single-molecule imaging movies to determine $h_{incidental}$). Furthermore, $h_{observed}$ would not depend on the number densities of AMPAR subunits: $h_{incidental}$ and $h_{composite}$ would not be affected by the number density, and c would also be independent of the number density, because the true binding of two molecules only occurs after they enter the colocalization range of 240 nm.

Since the exponential decay constants for $h_{observed}$ (between 164 and 335 ms) were considered sufficiently long as compared with those for $h_{incidental}$ (between 49 and 55 ms), we did not attempt to make any corrections. In addition, at the ranges of the number densities of molecules employed here, the monomer reference molecules did not exhibit apparent trimers and tetramers, and therefore, we did not have to consider $h_{incidental}$ ($c \approx 1$).

These points were not explained in the original manuscript, as indicated by Reviewer #1, mainly due to the limitations of the number of words in the manuscript and the existence of previous publications using the same method (Suzuki et al., Nat. Chem. Biol. 2012, ref#41; Kasai et al., Cell Biochem. Biophys. 2018, ref#69). In the revised manuscript, we stated that the lifetimes of the dimers and greater multimers described in this report should be considered as approximate measures, rather than absolute lifetimes (at the end of the first paragraph on **p. 10**), and the explanation given in the previous paragraphs about the estimates of the colocalization lifetimes is now included in the **Method section** (**pp. 32-34**).

Regarding the conclusion put forward by the authors:
1. The assembly process of AMPAR is complicated and involves crossover of large globular domains between subunits in the dimer-of-dimer assembly. Also, intra- and inter-dimer interfaces involve large areas of strong connection. How this can be explained by rapid assembly, and particularly disassembly, of the tetramers near and in the synapse?

As stated by Reviewer #1, the X-ray crystallographic data suggest extensive intra- and inter-dimer interactions in an AMPAR tetramer (Sobolevsky et al., Nature 2009; Herguedas et al., Science 2016; Zhao et al., Science 2019; cited as ref#47, #45, and #33, respectively in the revised manuscript). However, before the present study, the relationship between these interactions and the kinetics for the formation and dissociation of AMPAR tetramers had never been investigated. Namely, the interactions found in crystallography cannot predict whether the tetramer lifetime is, for example, a fraction of a second or several hours. We think the results described in this paper would be a good starting point for clarifying the

relationship between the interactions found in X-ray crystallography and kinetic observations in living cells. This point is now described in the Discussion section (the third paragraph on **p. 21**).

2. AMPAR are tightly associated with auxiliary proteins, particularly TARPs, which were shown to associate with the receptor already in the ER. TARPs were also shown to be particularly important for the lateral diffusion of AMPAR and for their encoring/immobilization at the synapse via phosphorylation/de-phosphorylation of the TARP C-tail. TARP tightly bind in between AMPAR subunits (i.e. requiring at least dimers if not tetramers for association). How this can be explained/fitted in the model?

We experimentally addressed this point, using the HEK293 model system, by observing (1) the effect of TARP2(-mGFP) overexpression on the homodimer lifetimes of ACP-GluA1 and ACP-GluA2, and (2) the lifetime of TARP2-mGFP binding to GluA1 and GluA2 (monomers and homodimers). We obtained the following results.

(1) The effect of TARP2 overexpression on the homodimer lifetimes of GluA1 and GluA2 in HEK cells

For this experiment, mGFP-fused to the C-terminus of TARP2 (TARP2-mGFP) was employed. Using the mGFP-fusion protein was necessary to evaluate the expression levels of TARP2, so that the cells expressing similar levels of TARP2 could be used for all of the experiments. Overexpression at levels 47 (±30) times higher than those of GluA1 and GluA2 was employed (n = 13 and 12 cells, respectively). The fusion of mGFP to TARP2's C-terminus might inhibit TARP2 binding to PSD95 in neurons (Schnell et al., PNAS, 2002, ref#35; Bats et al., Neuron 2007, ref#36), but would not block its binding to GluA1 and GluA2 (Hastie et al., PNAS 2013, ref#24; Bedoukian et al., JBC 2006, ref#37).

The overexpression of TARP2-mGFP did not significantly alter the homodimer lifetimes of GluA1 and GluA2 (**Fig. 5a** and **Supplementary Fig. 10a;** the first paragraph on **p. 12**), suggesting that TARP2 did not stabilize the homodimers of GluA1 and GluA2, perhaps at variance with the general belief, although it might have enhanced the formation of these homodimers (we could not determine this, as we only measured the dissociation rates).

(2) The lifetime of TARP2-mGFP binding to monomers and homodimers of GluA1 and GluA2 in HEK cells

Next, we examined whether TARP2-mGFP binds to GluA1 and GluA2 (monomers and homodimers), and whether the durations of TARP2 binding to homodimers of GluA1 and GluA2 were longer than to their monomers. We found that TARP2-mGFP bound to both monomers and dimers of GluA1 and GluA2, but quite unexpectedly (Shanks et al., J. Neurosci. 2010, ref#31), its binding was quite transient, lasting for fractions of a second.

The exponential lifetimes of TARP2-mGFP binding to GluA1 monomers and homodimers were quite similar; i.e., 190 and 184 ms, respectively (no significant difference; *P* = 0.96; **Fig. 5b, c**), and those to GluA2 monomers and homodimers were also quite similar; i.e., 229 and 206 ms, respectively (no significant difference; *P* = 0.12; **Supplementary Fig. 10b, c**). Again, perhaps at variance with the general belief, the binding lifetimes of TARP2 to homodimers of GluA1 and GluA2 were not significantly longer than those to monomers of GluA1 and GluA2.

The lifetimes of TARP2-mGFP binding to GluA1 and to GluA2 were also not significantly different (*P* = 0.081 for monomer comparison and *P* = 0.91 for dimer comparison). Note that, like the other quantitative results, the SEM, the number of experiments, and other statistical analysis results are shown in **Supplementary Table 7** (all statistical analyses of the colocalization duration distributions were performed by using the log-rank test).

Homotetramers of GluA1 and GluA2 occurred quite rarely, and their colocalizations with TARP2-mGFP took place even more rarely, and thus we were unable to evaluate the colocalization lifetimes of TARP2-mGFP and homotetramers of GluA1 and GluA2. However, if the TARP2-mGFP binding had greatly stabilized the homotetramers of GluA1 and GluA2, as might have been expected from the single-particle cryo-EM data for the structure of the complex of TARP2 and GluA2 tetramers (Twomey et al., Science 2016, ref#38; Zhao et al., Nature 2016, ref#39), then we should have been able to readily find the complexes of TARP2-mGFP and GluA1 or GluA2 homotetramers. Therefore, we concluded that the binding of TARP2 to GluA1 and GluA2 homotetramers was also quite transient, and thus did not substantially stabilize the homotetramers.

In summary, perhaps at variance with previous expectations, we concluded that TARP2 neither formed stable complexes with monomers or homodimers of GluA1 and GluA2, nor induced the stabilization of GluA1 and GluA2 homodimers (and probably homotetramers). TARP2 only formed transient complexes with both monomers and homodimers (and probably homotetramers) of GluA1 and GluA2, and the off rates of TARP2 from the homodimers of GluA1 and GluA2 were not significantly different than those from their respective monomers (approximately inverse of ~200 ms).

The (apparently) stable complexes of TARP2 with AMPAR subunits, which have been suggested to exist in synapses, may occur due to the higher concentrations of TARP2 bound to (and perhaps immobilized on) PSD95 clusters in the synapse, which might induce continual rebinding of AMPAR subunits to the concentrated TARP2 on PSD clusters as soon as AMPAR subunits dissociate from TARP2. This would make the AMPARs virtually immobile, confining them in the PSD95-concentrated synaptic regions (no long-range lateral diffusion),

as pointed out by Reviewer #1. Such a dynamic mechanism for molecular assemblies might be generally important, in addition to synaptic molecular assemblies (such as focal contacts and adhesions and immunological synapses), for inducing and maintaining molecular assemblies that can be regulated and disintegrated when the needs arise.

These results and explanations for the consistencies with previous observations and models have now been included in the revised manuscript in a **new subsection, entitled "TARP2 transiently binds to GluA1 and GluA2 (monomers, dimers, and probably tetramers) in the PM"** on **pp. 11-13** and the new figures (**Fig. 5** and **Supplementary Fig. 10**).

3. The study would be much stronger if the authors could provide some data for LTP.

Thank you very much for this excellent suggestion. We intensively discussed this possibility, but in conclusion, we presently do not want to include this part in the paper. This is because we plan to perform a comprehensive study on this subject matter and wish to publish it separately. In addition, we do not want to make this report more voluminous, to preserve its readability.

In summary, the study challenges a general believe in the field that AMPAR delivered to the plasma membrane are fully formed functional tetramers, and most probably also associated with auxiliary proteins, the TARPs at least. As such, the study is important enough to be published at a high profile journal, even if only for the sake of being challenged by future studies. If the authors are right, this would entirely change how we think about the mechanisms of induction and formation of synaptic plasticity.

Thank you very much for your kind statement.

Reviewer #2 (Remarks to the Author):

This manuscript from Morise and their colleagues present an elegant study about the molecular composition of AMPA receptors using single molecule imaging techniques. AMPARs are functional tetramers, and the structure has been believed stable. However, their results suggest that GluA1 and GluA2 may be expressed as monomers or dimers in most of time on the plasma membrane, at least under some conditions. The mobility of monomers and dimers are much higher than tetramers.

They found that in HEK cells, fluorescent intensity of labeled AMPAR is consistent with 1- to 4-mers, with a significant fraction of them as monomer and dimer. The ratio depends on the surface concentration of the monomer, and was consistent with dynamic equilibration. The lifetime of the tetramers was measured to be surprisingly short: an order of 100 ms. Ca2+ transients caused by glutamate was proportional to their tetramer density, but supra-linear to the number density.

Data in neurons are done by antibody, and overall less convincing. They occasionally observed transient collocalization of AMPAR subunits, suggesting rapid subunit exchanges. The diffusion appears to be consistent with that of monomeric subunits in dendritic shafts.

Overall, the manuscript is well written, and many of their claims appear to be well supported by their data. The results have important implications in AMPAR turnover in and out of synapses. However, there are several technical concerns:

Thank you very much for your critical reading of our manuscript, and providing us with constructive advice and suggestions for improving it.

1. Diffusion measurements in neurons are performed with antibody, and it would be problematic. They argue that antibody would not cause any cross-linking based on their diffusion data. However, since it is known that antibody can crosslink AMPAR and change diffusion (like Penn, … Choquet, Nature, 2017), this argument would be not so convincing. Also, since the diffusion in dendrite is so different from HEK cells, it would be necessary to have an independent control. One possibility is to use overexpressed AMPAR subunits, just like in their HEK cell measurements.

As recommended by Reviewer #2, as well as Reviewer #1 and the Editor, we expressed Halo7-GluA1 and Halo7-GluA2 in primary neurons, and observed their diffusion behaviors as well as the homo colocalization-codiffusion of GluA1 and GluA2 in the plasma membrane

(PM) of the hippocampal neurons in culture (in HEK293 cells, ACP-GluA1 and GluA2 were used more extensively, as compared with Halo7-GluA1 and GluA2. However, we were unable to express ACP-GluA1 and GluA2 in neurons). As explained below, virtually all of the results obtained by using fluorescently-labeled monoclonal antibodies (mAbs) as probes, as shown in the original manuscript, were reproduced using Halo7-GluA1 and Halo7-GluA2 expressed in the neuronal PM, except for the homo-colocalization lifetimes (exponential decay time constants) of Halo7-GluA1 and Halo7-GluA2, which were 30-40% shorter than the lifetimes obtained with the mAb probes. As stated by Reviewers #1 and #2, since the use of mAb probes is less favorable, we used the data obtained with Halo7-GluA1 and Halo7-GluA2 expressed in the neuronal PM in the revised manuscript (**Figs. 7** and **8**; **Supplementary Figs. 7a, 12, 13c, 14c, 15c, d, 16,** and **19**). Note that the expression levels of Halo7-GluA1 and Halo7-GluA2 are far lower than those of the endogenous GluA1 and GluA2: a single copy of Halo7-GluA1 and Halo7-GluA2 was found in less than every 10 synapses, avoiding the problems of overexpression. The data using mAb probes bound to endogenous GluA1 and GluA2 are now shown in the Supplementary figures, to confirm the results obtained with Halo7 probes (**Supplementary Figs. 13, 14, 15c, d,** and **17**).

The following are the summaries of the data obtained with Halo7-GluA1 and Halo7-GluA2 expressed in the neuronal PM. The first part is about their diffusion in the neuronal PM. The second part is about the homo colocalization-codiffusion of GluA1 and GluA2 in the dendritic-shaft PM.

(1) The diffusion coefficients found in the dendritic shaft PM (**Fig. 7c, d**; **Supplementary Fig. 12c, d**; the second paragraph on **p. 15**) were quite similar to those evaluated with fluorescently-labeled mAbs bound to endogenous GluA1 and GluA2 (**Supplementary Figs. 13c** and **14c; Supplementary Table 10**).

Following the advice of Reviewer #2 (point #3, to be discussed later in detail), we examined the data by using *ensemble-averaged* MSD-Δt plots (**Fig. 7d** and **Supplementary Fig. 12d**). We found that the ensemble-averaged MSD-Δt plots for the diffusion of Halo7-GuA1 and Halo7-GluA2 in the synaptic PM marked by Homer1b-EGFP could be fitted, as suggested by Reviewer #2, using an equation based on a model of confined diffusion (Kusumi et al., Biophys. J. 1993, ref#72), rather than that based on simple Brownian diffusion (linear fitting, which we used in the original report). The new results indicated a ~9-10x slowed diffusion rate from the diffusion coefficients in the dendritic-shaft PM plus confinement within a domain of ~109 nm, as shown in **Fig. 7d** and **Supplementary Fig. 12d** and discussed in the main text on the last paragraph on **p. 17** ~ the first paragraph on **p. 18**).

Meanwhile, the MSD-Δt plots for individual spots of Halo7-GluA1 and Halo7-GluA2 (i.e., not ensemble-averaged) in the synaptic PM (on Homer1b-EGFP) are quite noisy, and the signal-to-noise ratios of the MSD-Δt plots for individual fluorescent spots did not allow reasonable fitting using a function describing the model of confined diffusion, because this function is more complicated than the linear function describing the simple-Brownian diffusion. Therefore, we could not evaluate the diffusion coefficients and the confinement sizes for individual fluorescent spots, and thus we do not present the histograms showing their distributions in the revised manuscript. In the revised manuscript, we only show the MSD-Δt plots ensemble averaged over all of the spots that existed in the synaptic PM, and the mean (± SEM) values of the diffusion coefficients obtained from the MSD-Δt plots ensemble averaged over all of the molecules that existed in the synaptic PM (**Fig. 7d** and **Supplementary Fig. 12d**).

(2) Homo colocalization-codiffusion of GluA1 and GluA2 in the dendritic-shaft PM was also re-examined using Halo7-GluA1 and Halo7-GluA2 expressed in the dendritic-shaft PM. Both Halo7-GluA1 and Halo7-GluA2 exhibited frequent transient colocalization-codiffusion events. However, their lifetimes (exponential decay times) were 104 and 95 ms for Halo7-GluA1 and Halo7-GluA2, respectively (the last paragraph on **p. 14** ~ the first paragraph on **p. 15**; **Fig. 7b** and **Supplementary Fig. 12b**), which were shorter than those found using antibody-bound GluA1 and GluA2 (163 and 129 ms, respectively; **Supplementary Figs. 13b** and **14b**). With the bound monoclonal antibodies, the molecules undergoing colocalization-codiffusion might be transiently crosslinked by monoclonal antibodies, which may have prolonged the durations of colocalization-codiffusion. This explanation is included in the caption to **Supplementary Fig. 13b**.

In fact, at the time of the original submission, our attempts to express GluA1 and GluA2 conjugated with the tag proteins ACP and Halo7 in primary neurons had been unsuccessful, and thus we used monoclonal antibodies. The comments by Reviewers #1 and #2 as well as by the Editor strongly motivated us to try harder to express tag-protein-conjugated GluA1 and GluA2 in neurons, and as a result, we obtained data under better conditions (the expression became successful when we used the pCAGplay vector and the signal peptide of IL6). Therefore, we are extremely thankful for this important point raised by Reviewers #1 and #2 and the Editor.

2. They estimated the diffusion rate of monomers, dimers and tetramers in neurons from the diffusion of synthetic molecules, calibrated from HEK cell results. However, it would be important to measure the diffusion of monomer AMPAR subunit in neurons. This could be done by measuring overexpressed DeltaNTD in neurons (it may necessary to downregulate endogenous proteins with shRNA or CRISPR).

Following the advice of Reviewer #2, we examined the diffusion of Halo7-GluA1ΔNTD in the dendritic-shaft PM.

However, before explaining the new results of Halo7-GluA1ΔNTD in the dendritic-shaft PM, let us explain the revision of the diffusion coefficients of the monomeric reference molecules, Cy3-DOPE and Halo7-CD47, in the dendritic-shaft PM. Previously, to evaluate the diffusion coefficients in the dendritic PM, we used trajectories with entire lengths comparable to those used for the estimate in HEK cells (30 frames = 1 s for monomers and the entire durations of dimers and greater oligomers, because their lifetimes are shorter than 1 s). However, we have now realized that this method underestimated the diffusion coefficients in the dendritic shaft PM. Since the width of the dendritic shaft is narrow, rapidly diffusing molecules disappeared faster, because they quickly moved out of the basal PM and diffused into the PM in the top part of the shaft (TIR excitation light does not reach there, and thus we cannot visualize the molecules that existed there). Therefore, by selecting only the trajectories that were as long as those in HEK cells, we actually ended up selecting slower trajectories. So, this has been changed to any trajectories with lengths of 11 frames or longer (≥ 367 ms; this enabled the measurement of diffusion coefficients up to ~0.7 $\mu m^2/s$ for molecules diffusing in the middle part of the dendritic shaft with a width of ~2 μm; in retrospect, this was previously ~0.3 $\mu m^2/s$).

Using the new method, the median diffusion coefficients of Cy3-DOPE and Halo7-CD47 in the dendritic-shaft PM were 0.16 and 0.14 $\mu m^2/s$, respectively (their distributions are shown in **Supplementary Fig. 11a**; **Supplementary Table 9**), as compared with the previous median values of 0.095 and 0.083 $\mu m^2/s$, respectively. Therefore, the diffusion coefficients in the dendritic shaft PM are smaller than those in the HEK-cell PM by factors of 1.534 and 1.593 for Cy3-DOPE and Halo7-CD47, respectively (an average value for these two values, 1.56, is now employed). This ratio was previously 2.0. This change is now included in the second paragraph on **p. 15**.

The median diffusion coefficient of Halo7-GluA1ΔNTD expressed in the dendritic shaft PM was smaller than that in the HEK-cell PM by a factor of 1.73 (0.087 and 0.15 $\mu m^2/s$ in the dendritic shaft PM and in the HEK-cell PM, respectively; for their distributions, see **Fig. 7c**; see related text in the second paragraph on **p. 16**). This ratio of 1.73 is quite close to the ratio of 1.56 for the monomer reference molecules Cy3-DOPE and Halo7-CD47 (as described in the previous paragraph). This means that the reduction in the diffusion coefficient of Halo7-GluA1ΔNTD in the dendritic shaft PM, as compared with that in the HEK-cell PM, was about the same as (or only slightly smaller than) that for the monomer reference molecules, suggesting that Halo7-GluA1ΔNTD hardly formed complexes with the endogenous AMPAR

subunits in the dendritic-shaft PM. Consistent with this result, the median diffusion coefficient of Halo7-GluA1ΔNTD was virtually the same as that predicted for GluA1 monomers (0.087 and 0.094 $\mu m^2/s$, respectively; non-significant difference, $P = 0.54$; **Fig. 7c**), further supporting the conclusion that Halo7-GluA1ΔNTD does not extensively form complexes with the endogenous AMPAR subunits in the dendritic-shaft PM. These results are now described in the revised manuscript in the second paragraph on **p. 16** in the main text (**Fig. 7c**).

For predicting the diffusion coefficients of the monomers, dimers, and tetramers of GluA1 and GluA2 in the dendritic shaft PM from their measured values in the HEK-cell PM, we used the ratio of 1.56 determined by using the monomer reference molecules Cy3-DOPE and Halo7-CD47 (the second paragraph on **p. 15**), rather than 1.73 evaluated by using Halo7-GluA1ΔNTD. Although we could use the 1.73 ratio determined with Halo7-GluA1ΔNTD, we preferred using the ratio of 1.56, because Halo7-GluA1ΔNTD could form dimers and tetramers with the endogenous AMPAR subunits, as it exhibited a slight tendency to form homo-dimers and tetramers (**Fig. 2d**).

In the dendritic-shaft PM, the median diffusion coefficient of Halo7-GluA1 was significantly smaller than that of Halo7-GluA1ΔNTD (0.073 and 0.087 $\mu m^2/s$, respectively; $P = 1.7 \times 10^{-3}$; **Fig. 7c**; the third paragraph on **p. 16**). This is probably because Halo7-GluA1 forms dimers and tetramers with endogenous AMPAR subunits more extensively than Halo7-GluA1ΔNTD.

3. For their diffusion data in neurons, they should present the relationship between mean square distance and time, so that the readers can see that the diffusion is normal (they present MSD data for HEK cells in S-Fig. 8). This is especially important for diffusion in synapses, because the movement may show saturation as they may be trapped in small compartments. 200 ms may be too long for measurements of diffusion.

Following the suggestion by Reviewer #2, we now show the relationship between the ensemble-averaged MSD and the time (time interval) for Halo7-GluA1 and Halo7-GluA2 in the dendritic-shaft PM and on Homer1b (**Fig. 7d** and **Supplementary Fig. 12d**). As suggested by Reviewer #2, the plots obtained in the Homer1b area indicate leveling off (visible after ~0.1 s in **Fig. 7d** and **Supplementary Fig. 12d**, particularly the figures on the right). In the original manuscript, since we considered linear approximation to be simpler and thus better, we employed linear fitting in the range of 2-15 steps (as we did for molecules in the HEK-cell PM and the dendritic-shaft PM). However, since the MSD-Δt plots suggested that the diffusion characteristics of the molecules located in the Homer1b area could be better described by the model of the molecules being trapped in small compartments, we fitted the MSD-Δt plot using the equation representing confined diffusion

(Kusumi et al., Biophys. J. 1993, ref#72). The result indicated that these molecules were confined in a compartment with an average size of ~109 nm (for details, see **Fig. 7d** and its caption; the last paragraph on **p. 17** ~ the first paragraph on **p. 18**).

Minor

1.Figure 6: Homer would be not a marker of spines but synapses. This depends of course on the developmental stage etc, but their image does not show clear dendritic spine morphology. Perhaps they should call this "synapse" rather than "spine".

Since the spatial resolution in our TIRF observation of Homer1b is about 200 nm, a synapse with a diameter of ~300 nm, for example, would appear like a region with a diameter of ~500 nm. Therefore, in the original manuscript, we refrained from calling the Homer1b region a synapse. However, we now agree with Reviewer #2 that it would be better to refer to the Homer1b region as a synapse, rather than a spine. So, in the revised manuscript, they are now called "synapse" or "synaptic region".

REVIEWERS' COMMENTS:

Reviewer #1 (Remarks to the Author):

The revised manuscript is satisfactory
I have no further comments

Reviewer #2 (Remarks to the Author):

The authors significantly improved the manuscript. I think that their finding about the presence of monomeric and dimeric AMPARs in neurons is very important and their data sufficiently support their claims in their revised manuscript. I recommend publication.
